# Boronate Covalent and Hybrid Organic Frameworks Featuring P^III^ and P=O Lewis Base Sites

**DOI:** 10.1002/chem.202001960

**Published:** 2020-09-01

**Authors:** Piotr Pacholak, Krzysztof Gontarczyk, Radosław Kamiński, Krzysztof Durka, Sergiusz Luliński

**Affiliations:** ^1^ Faculty of Chemistry Warsaw University of Technology Noakowskiego 3 00-664 Warsaw Poland; ^2^ Department of Chemistry University of Warsaw Żwirki i Wigury 101 02-089 Warsaw Poland

**Keywords:** coordination polymers, covalent organic frameworks, frustrated Lewis pairs, quantum nature of host-guest interactions, structural modeling

## Abstract

Two covalent organic frameworks comprising Lewis basic P^III^ centers and Lewis acidic boron atoms were prepared by poly‐condensation reactions of newly obtained tris(4‐diisopropoxyborylphenyl)phosphine with 2,3,6,7,10,11‐hexahydroxytriphenylene and 2,3,6,7‐tetrahydroxy‐9,10‐dimethylanthracene. Obtained materials exhibit significant sorption of dihydrogen (100 cm^3^ g^−1^ at 1 bar at 77 K), methane (20 cm^3^ g^−1^ at 1 bar at 273 K) and carbon dioxide (50 cm^3^ g^−1^ at 1 bar at 273 K). They were exploited as solid‐state ligands for coordination of Pd^0^ centers. Alternatively, in a *bottom‐up* approach, boronated phosphine was treated with Pd_2_dba_3_ and poly‐condensated, yielding hybrid materials where the polymer networks are formed by means of covalent boronate linkages and coordination P−Pd bonds. In addition, the analogous materials based on phosphine oxide were synthesized. The DFT calculations on framework–guest interactions revealed that the behavior of adjacent boron and phosphorus/phosphine oxide centers is reminiscent of that found in Frustrated Lewis Pairs and may improve sorption of selected molecules.

## Graphical abstract

**The first boron‐phosphine covalent organic framework** (BP‐COF) is reported, which can be considered as a new group of functional porous materials joining characteristics of frustrated Lewis pairs (FLPs) with enhanced sorption selectivity and solid‐state ligands for the coordination of transition‐metal atoms.

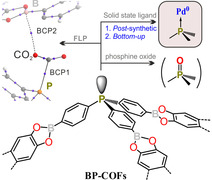

## Introduction

There is a continuous interest in covalent organic frameworks (COFs)—a class of porous organic materials composed of light elements such as carbon, boron, oxygen, nitrogen, silicon, and sulfur connected by strong covalent bonds.^1^, ^2^, ^3^, ^4^, ^5^, ^6^ Since the first report by Yaghi and co‐workers in 2005,^7^ numerous COF architectures were designed. From the structural topology point of view they can be divided in two major groups. Two‐dimensional (2D) COFs are obtained from building blocks possessing planar structures.^8^ In contrast, three‐dimensional (3D) COFs are formed starting with precursors featuring a general 3D (e.g., tetrahedral) topology which imposes a structure of a resulting polymer network. Within this group, classical examples include highly porous materials such as COF‐102 and COF‐103 based on tetraboronic acids C[*p*‐C_6_H_4_B(OH)_2_]_4_ and Si[*p*‐C_6_H_4_B(OH)_2_]_4_, respectively.^9^

The use of organophosphine linkers for the preparation of various multifunctional porous coordination polymers dates back to 2008. Since then, a number of materials were designed, especially by Humphrey and co‐workers (phosphine coordination materials, PCMs).^10^, ^11^, ^12^, ^13^, ^14^, ^15^, ^16^, ^17^, ^18^, ^19^, ^20^, ^21^, ^22^, ^23^, ^24^, ^25^ It should be noted they generally comprise carboxylic groups coordinating metal secondary building units (SBUs). The pseudo‐tetrahedral geometry around phosphorus centers favors the formation of 3D frameworks. The presence of lone electron pair at the P^III^ centers provides a possibility for *pre*‐ or *post*‐synthetic functionalization through alkylation,^16^, ^26^ arylation^27^ or chalcogenation.^28^ Such materials are also considered as solid‐state ligands (SSLs) for coordination of transition metals.^12^, ^29^, ^30^, ^31^, ^32^, ^33^, ^34^, ^35^ Regarding the latter ability, the most common strategy involves the formation of robust coordination polymers with metals (Ca, Zr, Sc) weakly coordinated to phosphorus and hard‐ligand donors (carboxylates, imidazolates, alkoxylates). In such porous networks, the softer phosphorus function allows the facile coordination of precious metal atoms including Pd^0^, Rh^I^, Ir^I^, Au^I^, Ag^I^, as well as low‐valent Cu^I^, Co^II^ metals. Importantly, their catalytic performance is usually retained from corresponding single‐molecule phosphine‐based catalysts. Thus, such materials offer a very attractive way to transfer catalytic processes from homogenous to heterogeneous environments. This concept can also be used to achieve stronger and more selective binding of small‐molecule substances, which can further be exploited for storage and separation processes. In addition to the P^III^ materials, the analogous MOFs constituting phosphine oxides functions are obtained and used as a solid‐state platforms for the lanthanide‐metal coordination (La^III^, Dy^III^).^17^, ^19^

**Scheme 1:**
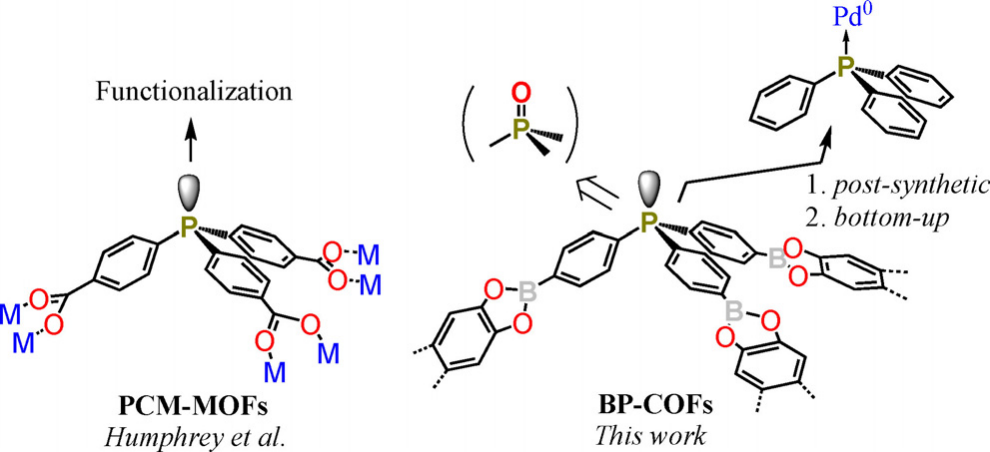
New class of COF materials based on boronated phosphines.

Although the chemistry of PCM‐MOFs is already well developed, the analogous COFs containing phosphine or phosphine oxide fragments have not been considered so far. Limited progress in this field was due to the lack of procedures allowing for the facile synthesis of phosphine‐based COF precursors. In particular, boronated COFs are especially interesting due to the presence of weakly acidic boron centers along with stronger Lewis base phosphorus sites. In our efforts for the synthesis of functional porous materials^36^, ^37^ we have developed a new approach to synthesize boron‐phosphine COFs (BP‐COFs) (Scheme 1). Their non‐planar tripodal topology is unique for all COF precursors. Thus, the prepared materials cannot be regarded as typical 3D COFs, which are based on nodes featuring the 43*m* point‐group symmetry. The presence of Lewis base P^III^ or P=O oxygen atoms and weakly acidic boron centers may significantly enhance the sorption properties of such materials. Furthermore, they can serve as a robust platform for the coordination of transition metals. Hence, in the current contribution we present *pre*‐ and *post*‐synthetic modifications of COFs with palladium(0) using labile Pd_2_dba_3_ complex as a metal source.

## Results and Discussion

**Figure 1:**
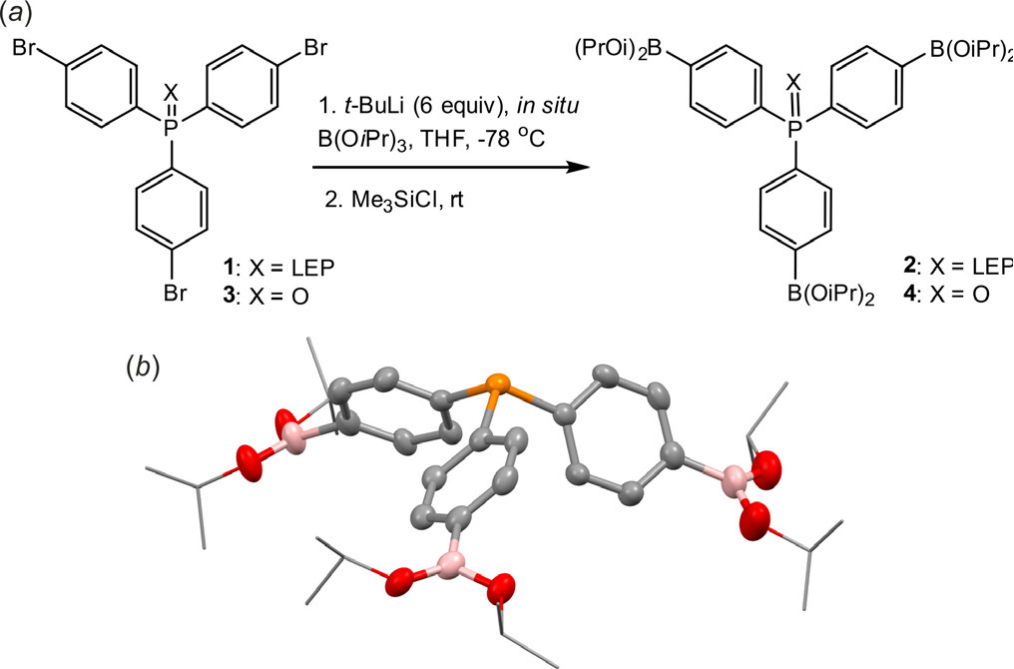
(*a*) Synthesis of triboronated phosphine **2** and phosphine oxide **4**. (*b*) The molecular structure of **2** (ellipsoids drawn at the 50 % probability level, H‐atoms and disordered sites omitted for clarity). LEP=Lone Electron Pair.

Tris(4‐bromophenyl)phosphine **1**, a starting material for the preparation of **2**, was obtained on a large scale (0.5 mol of the starting 1,4‐dibromobenzene) from the metathesis of PCl_3_ with 4‐bromophenyllithium according to the published procedure.^38^ It was subjected to triple bromine–lithium exchange followed by a boronation with trialkyl borate reagent. After careful optimization, the approach based on the in situ addition of *t*BuLi (6 equiv) to the mixture of **1** and B(O*i*Pr)_3_ in THF at −78 °C was found to give the best results (Figure 1 a). In our first attempts the reaction mixture was hydrolyzed in order to isolate tris(4‐dihydroxyborylphenyl)phosphine **2 a** as a precursor of COFs. However, ^1^H and ^31^P NMR analyses revealed that **2 a** exists in equilibrium with its hydrated zwitterionic form (**2 a’**) featuring protonated phosphorus atom and the anionic boronate group (Scheme S**1**, Supporting Information). Furthermore, we have observed its oxidation under air to corresponding phosphine oxide (**4 a**), which is accompanied by a slow partial deboronation. Therefore, we decided to isolate compound **2** by treating its tris(boronic) ate complex precursor with Me_3_SiCl followed by removal of volatiles and extraction with heptane. Compound **2** was obtained in a pure form as colorless crystals in multigram quantities and reasonable yield (50 %). The structure of **2** was confirmed by multinuclear NMR spectroscopy and X‐ray diffraction (Figure 1 b). Notably, the crystal structure lacks intermolecular interactions between Lewis acidic boron atoms and Lewis base phosphorus centers.

The synthesis of boronated triphenylphosphine oxide precursor **4** involved the in situ Br/Li exchange/boronation of brominated precursor **3** 39 using the protocol analogous to that described for **2**. Compound **3** was isolated as a yellow solid and characterized by ^1^H, ^11^B and ^31^P NMR spectroscopy. Unlike **2**, it is only sparingly soluble in CDCl_3_, presumably due to extensive aggregation in solid state through the P=O→B dative bonds.

**Figure 2:**
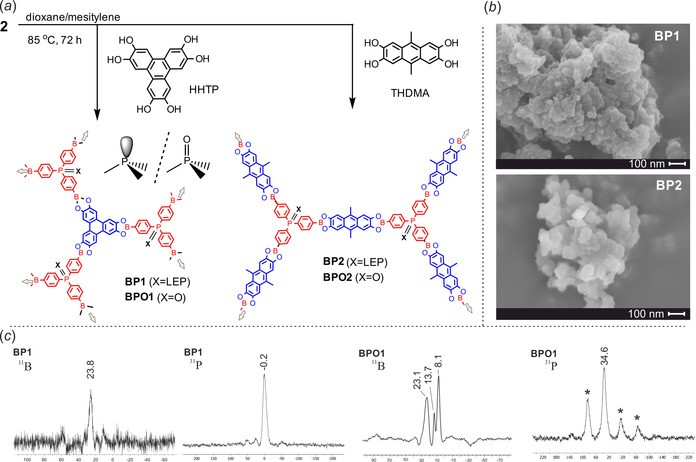
(*a*) Synthesis of **BP1**–**2** and **BPO1**–**2** porous materials. (*b*) SEM images of **BP1**–**2**. (*c*) ^11^B and ^31^P MAS NMR spectra of **BP1** and **BPO1** recorded at the spinning rate of 10 kHz. Asterisks (*) indicate peaks arising from spinning side bands.

As shown in Figure 2 a the syntheses of materials **BP1**–**2** were performed by polycondensation reactions of **2** with 2,3,6,7,10,11‐hexahydroxytriphenylene (HHTP, 1 equiv), or 2,3,6,7‐tetrahydroxy‐9,10‐dimethylanthracene (THDMA, 1.5 equiv), respectively. A general protocol similar to those developed for the preparation of other boronate ester COFs was employed.^1^, ^2^, ^6^, ^7^, ^8^, ^9^ Thus, a stoichiometric mixture of **2** and a polyol was stirred in a 1:1 (v/v) mesitylene −1,4‐dioxane mixture at 85 °C followed by repeated washing of a crude product with anhydrous THF and final drying under high vacuum at 150 °C. The prepared materials are grayish‐green (**BP1**) or olive‐green (**BP2**) powders. Their hydrolytic degradation in wet [D_6_]DMSO (with added D_2_O) and ^1^H NMR analyses of resulting samples showed that their composition was in agreement with the theoretical stoichiometry. Since the precursor **2** is not the boronic acid but the respective ester, its poly‐condensation reactions with HHTP and THDMA can be classified as transesterification‐based processes. We suggest they should proceed more easily and faster than dehydrative poly‐condensation reactions which occur during formation of COFs from free boronic acids and are typically conducted solvothermally although some examples involving mild conditions were reported.^40^ Thus, the synthesis of **BP1**–**2** was repeated at room temperature and both materials were obtained in good yields. Furthermore, their composition and appearance is very similar to the samples obtained with the heating applied. On the other hand, high reaction rates favor amorphization. Following the same protocol the synthesis of phosphine oxide‐based materials (**BPO1**–**2**) was performed by poly‐condensation reactions of **4** with HHTP (1 equiv) or THDMA (1.5 equiv), respectively.

**Figure 3:**
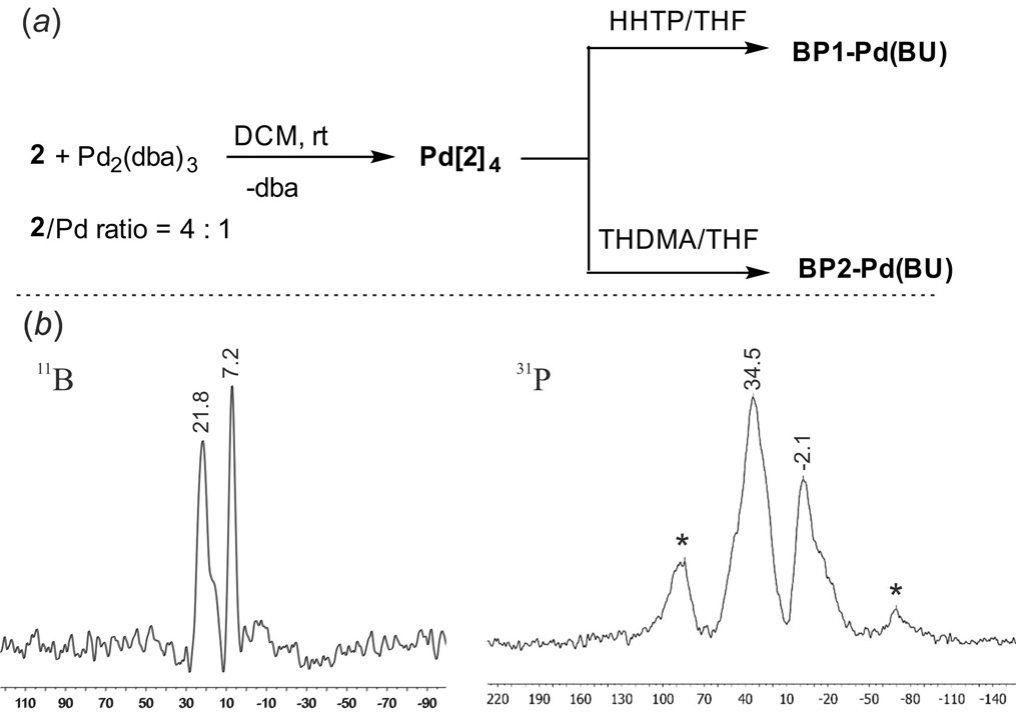
(*a*) Synthesis of Pd‐functionalized COF materials using bottom‐up approach. (*b*) ^11^B and ^31^P MAS NMR spectra of **BP1‐Pd(BU)** recorded at the spinning rate of 10 kHz. Asterisks (*) indicate peaks arising from spinning side bands.

In the next step, hybrid materials containing Pd^0^ metal centers were prepared utilizing two general strategies: (a) *bottom‐up* approach involving Pd^0^ coordination to the precursor **1**, followed by polycondensation with HHTP/THDMA (**BP1‐Pd(BU)**, **BP2‐Pd(BU)**), (b) *post*‐synthetic modification of previously obtained **BP1**–**2** COFs (**BP1‐Pd(PS)**, **BP2‐Pd(PS)**). It should be noted that the modification of organoboron polymers through metal complexation is still rather unexplored.^41^ The synthesis of **BP1‐Pd(BU)** and **BP2‐Pd(BU)** was performed in two steps as shown on Figure 3 a. At first, compound **2** was treated with Pd_2_(dba)_3_ at low temperature (ca. −50 °C) in DCM. The amounts of starting materials were taken to achieve the Pd:P ratio of 1:4.

Initially, the reaction mixture had a purple color due to the presence of dissolved Pd_2_(dba)_3_. However, a solution gradually turned olive‐brown on warming to ambient temperature indicating the progress of ligand exchange at the Pd atom and formation of a species of assumed stoichiometry **Pd[2]_4_** although it may equilibrate with **Pd[2]_2_** and/or **Pd[2]_3_** as well as free ligand **2**. The ^31^P NMR spectrum of this species showed a few resonances in the range of 24–26 ppm which confirms the formation of Pd‐P dative bonds.^42^ In the second step, the obtained complex was treated with a THF solution of a polyhydroxy linker, i.e., HHTP or THDMA, in a stoichiometric proportion with respect to **2**. The gradual precipitation of a voluminous brownish or olive‐green precipitate was observed during several hours. The slurry was stirred for 3 days at room temperature and filtered under argon followed by thorough washing of a solid with several portions of anhydrous DCM. Drying under high vacuum at 80 °C resulted in final materials as free‐flowing dark‐colored powders. Analysis of a yellow filtrate revealed the presence of free ligand (dba) in amounts slightly less (ca. 15 %) than those initially added with the used Pd precursor. It was not significantly contaminated by other compounds including boronated triphenylphosphine derivatives and polyhydroxy linkers, which indicates their quantitative incorporation in the bulk materials.

In the second approach, **BP1**–**2** materials were immersed in Pd_2_dba_3_/THF solution during 3 days. We assumed that the labile character of the complex should result in the attachment of Pd^0^ to P centers^43^, ^44^ on the surface of pores having size sufficient for penetration with Pd_2_dba_3_. In both cases, the purple solution containing the unreacted complex was removed and the resulting materials were washed thoroughly with DCM and dried under vacuum (10^−3^ torr, 50 °C) to give the final products **BP1‐Pd(PS)** and **BP1‐Pd(PS)** as dark powders.

The thermal stability of **BP1**–**2** (dried at 150 °C prior to analysis) was investigated under N_2_ atmosphere by TGA technique (Figure S1). A minor mass loss (up to 5 %) is observed up to 350 °C. It can be associated with the removal of adsorbed gases, and some volatile impurities (e.g., traces of solvents or by‐products resulting from completion of condensation reactions). A gradual decomposition starts as the total mass loss at 600 °C reaches ca. 40 % for both COF materials. To summarize, obtained boronate ester COFs exhibits comparably high thermal resistance consistent with the exclusive presence of strong covalent bonds. The presence of Pd nodes did not affect the thermal stability of **BP2‐Pd(BU)** as the decomposition started also at ca. 350–400 °C (Figure S2). In turn, **BP1‐Pd(BU)** material losses about 15 % of its mass when heating up to 200 °C, which is probably associated with the presence of significant amount of solvent and dba molecules resided in the porous framework.

^11^B MAS NMR spectrum of **BP1** (Figure 2 c) shows the resonance at ca. 24 ppm, i.e., in the range characteristic for trigonal boron atoms in arylboronic acids and esters.^45^ The ^31^P MAS NMR spectrum of **BP1** shows a sharp resonance at ca. 0 ppm, i.e., shifted downfield by ca. 6 ppm with respect to PPh_3_.^46^ Very similar ^11^B and ^31^P MAS NMR spectra were recorded for **BP2** (see Supporting Information, Figures S23, S24). Based on these results one can conclude that the plausible structure stabilization of **BP1**–**2** due to formation of strong dative interactions between boron and phosphorus atoms should be excluded. However, a slight deshielding (relative to PPh_3_) of ^31^P MAS NMR signals for both materials might indicate occurrence of weak communication between boron and phosphorus centers.^47^ The ^11^B MAS NMR spectrum of **BPO1** (Figure 2 c) shows the resonances at 8.1, 13.7, 23.1 ppm. The latter can be assigned to the trigonal boron atom featuring CBO_2_ environment whereas two remaining ones may indicate that some boron atoms are coordinated with oxygen atoms of P=O groups and/or THF guest molecules. The ^31^P MAS NMR spectrum of **BPO1** is characterized by persistence of intense spinning sidebands indicating that the chemical shift anisotropy frequency range is larger than the MAS rate. The spectrum has one isotropic chemical shift at 34.6 ppm consistent with the presence of triphenylphosphine oxide motifs. Very similar ^11^B and ^31^P MAS NMR spectra were recorded for **BPO2** (Figures S25, S26), thus confirming that the change of the linker topology does not significantly influence the ^31^P NMR chemical shift anisotropy characteristics of the studied nuclei. ^11^B MAS NMR spectra of the hybrid **BP1‐Pd(BU)** and **BP2‐Pd(BU)** materials (Figure 3 b) are in general analogous to those recorded for **BPO1**–**2** as they point to dative interactions between some boron atoms and oxygen‐based donors including remaining THF or dba molecules. The ^31^P MAS NMR spectrum of **BP1‐Pd(BU)** features a set of spinning sidebands with isotropic chemical shifts at −2.1 and 34.5 ppm (similar values of −0.7 and 36.3 ppm were recorded for **BP2‐Pd(BU)**). The former one can be assigned to free triphenylphosphine moieties which indicates that P^III^ nodes characteristic for **BP1** are also present in **BP1‐Pd(BU)** to a significant extent. However, the latter more intense (ca. two‐fold) signal can be assigned to triarylphosphine P atoms engaged in Pd−P dative bonds.^48^ It should be noted that the presence of P−Pd coordination is also supported by XPS data which are discussed below.

Scanning electron microscopy (SEM) indicates mesoporous morphology of **BP1**–**2** (Figure 2 b). This material forms sponge‐like agglomerates of irregular shape and dimensions in the range of 0.5–20 μm. The size of particles (presumably nanocrystallites) forming these structures are very small and ranges from 50–200 nm. The impregnation of such a COF structure with palladium centers does not change the general morphology of the structure (Figure S41).

**Figure 4:**
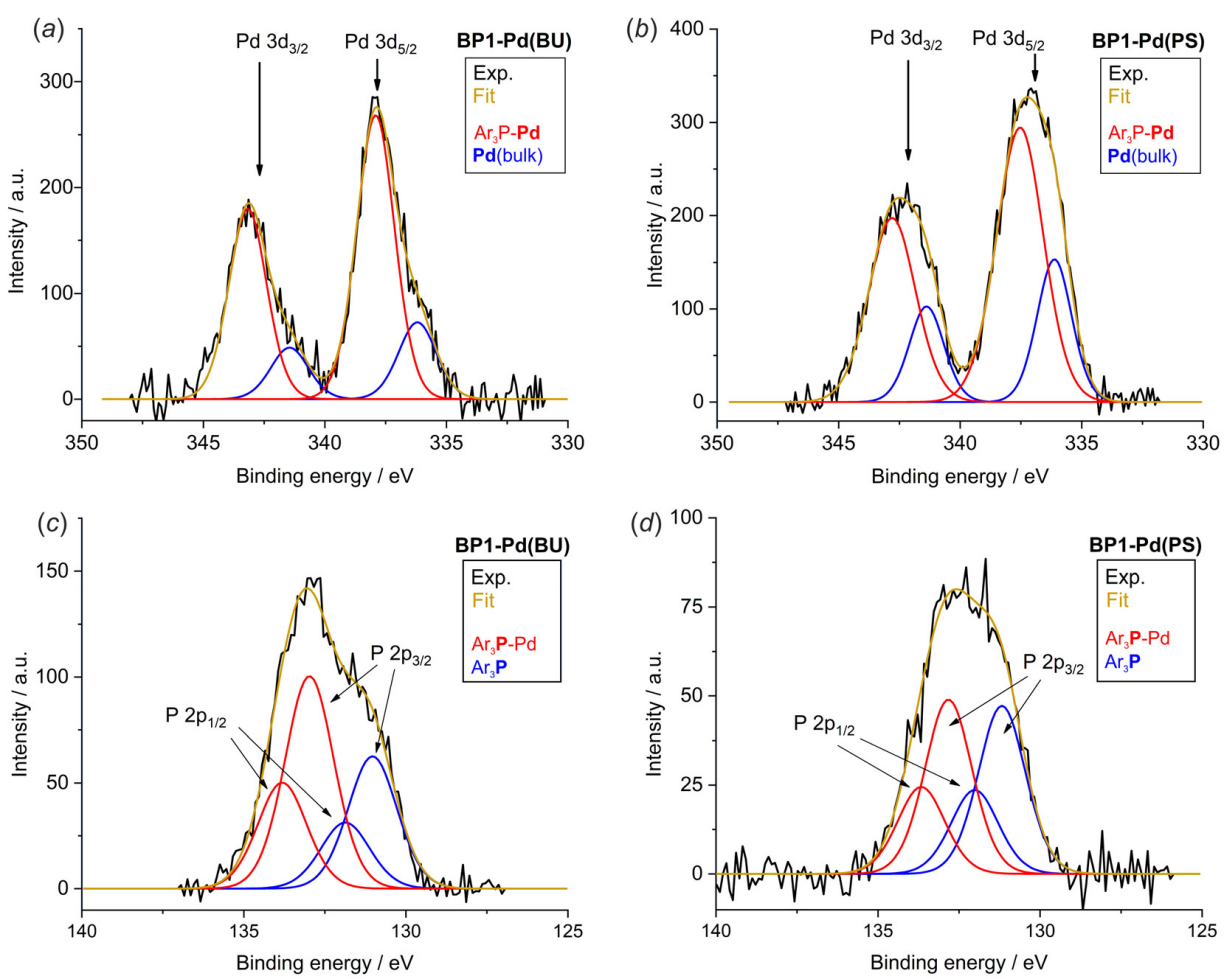
High‐resolution XPS spectra for **BP1‐Pd(BU)** (*a*, *c*) and **BP1‐Pd(PS)** (*b*, *d*): Pd 3d (*a*, *b*) and P 2p (*c*, *d*).

XPS analysis was employed to evaluate the amount of Pd centers adsorbed on the surfaces of COF materials obtained using *bottom‐up* and *post*‐synthetic methodologies. The survey of XPS spectra is shown in Figures S29**–**S32 in Supporting Information. Pd 3d and P 2p high‐resolution XPS spectra of **BP1‐Pd(BU)** and **BP1‐Pd(PS)** COFs are shown in Figure 4, while the C 1s, B 1s, and O 1s HR‐XPS spectra regions and analogous spectra for **BP2‐**derived hybrid materials are placed in the SI (Figures S33**–**S36).^49^ As indicated by the analysis of Pd 3d region, two different Pd species can be distinguished. The doublet at binding energies (B.E) of 341.5 eV (Pd 3d_3/2_) and 336.3 eV (Pd 3d_5/2_) can be assigned to bulk palladium nanoparticles—(**Pd**(bulk), Table 1).^50^ The bigger doublet is shifted towards higher binding energies (B.*E*=343.2 eV, 337.9 eV) and is associated with palladium centers coordinated to phosphorus atoms (Ar_3_P‐**Pd**).^51^ The appearance of metallic palladium on the COF surface results from the relatively low stability of Pd_2_(dba)_3_ precursor and may indicate that its decomposition followed by formation of Pd clusters is catalyzed by the COF surface. The deconvolution of XPS curves clearly shows that total amount of Pd atoms adsorbed in the **BP1‐Pd(BU)** and **BP2‐Pd(BU)** materials is much lower with respect to *post*‐synthetically impregnated COFs, which is consistent with stoichiometry of P/Pd centers in precursors. Furthermore, the Pd‐P/Pd(bulk) molar ratio is much lower for latter systems, specifically in case of **BP2‐Pd(PS)** Pd bulk atoms outnumber those bound to P atoms. The deconvolution of P 2p core level spectra for **BP1‐Pd(BU)** gives two doublets representing two set of P 2p_1/2_ and P 2p_3/2_ peaks. The peaks at 131.0 eV and 131.9 eV correspond to P atoms bound to three aromatic rings of the COF framework (Ar_3_ **P**). The adjacent peaks at higher binding energies (B.*E*=133.0 eV and 133.8 eV) can be attributed to the P centers coordinating palladium atoms (Ar_3_ **P**‐Pd). The comparison of Pd‐containing materials obtained using either *bottom‐up* or *post*‐synthetic approaches shows that proportion of free and bound P atoms is different in both material classes. In the former materials, the molar ratio of palladated vs. free phosphine (Ar_3_ **P**‐Pd/Ar_3_ **P**) is close to 1.5 indicating that more than half of P atoms is bound to Pd, while in the case of *post*‐synthetically modified COFs, this ratio is close to 1. Finally, as suggested by the molar ratio of coordinated P and Pd sites (Ar_3_ **P**‐Pd/**Pd**‐P), in the case of **BP1‐Pd(BU)** and **BP2‐Pd(BU)** each Pd center is bound to 3 or 4 P atoms. Thus, these COFs preserve the stoichiometry of its precursor **Pd[2]_4_** and can be treated as mixed‐hybrid networks composed of covalently linked organoboron network interconnected by tetra‐coordinated palladium centers. In turn, for **BP1‐Pd(PS)** and **BP2‐Pd(PS)** the molar ratio Ar_3_ **P**‐Pd/Ar_3_ **P** is close to 1, which indicates that the structure of these materials is mostly preserved from initial **BP1**–**2** COFs. Overall, it can be concluded that the *post*‐synthetically Pd‐functionalized COFs are more preferred for catalytic applications, where the density of the catalytic centers plays the major role, while the palladium centers would be more dispersed and electronically active in materials obtained from a bottom‐up approach.

**Table 1:** XPS‐derived phosphorus and palladium atom amounts located on the surface of corresponding COF materials. **Pd**(bulk) and **Pd**‐P denote bulk and bonded palladium atoms, Ar_3_ **P** and Ar_3_ **P**‐Pd correspond to three‐coordinate and Pd‐bonded P atoms, respectively. Values are provided in atomic percent. The respective molar ratios are given below.

	**BP1‐Pd(BU)**	**BP2‐Pd(BU)**	**BP1‐Pd(PS)**	**BP2‐Pd(PS)**
**Pd**(bulk)	0.05	0.08	0.23	0.75
**Pd**‐P	0.32	0.25	0.62	0.63
Ar_3_ **P**	0.64	0.66	0.72	0.83
Ar_3_ **P**‐Pd	1.03	0.98	0.75	0.71
**Pd**‐P/**Pd**(bulk)	6.40	3.13	2.70	0.84
Ar_3_ **P**‐Pd/Ar_3_ **P**	1.61	1.48	1.04	0.85
Ar_3_ **P**‐Pd/**Pd**‐P	3.22	3.9	1.21	1.13

**Figure 5:**
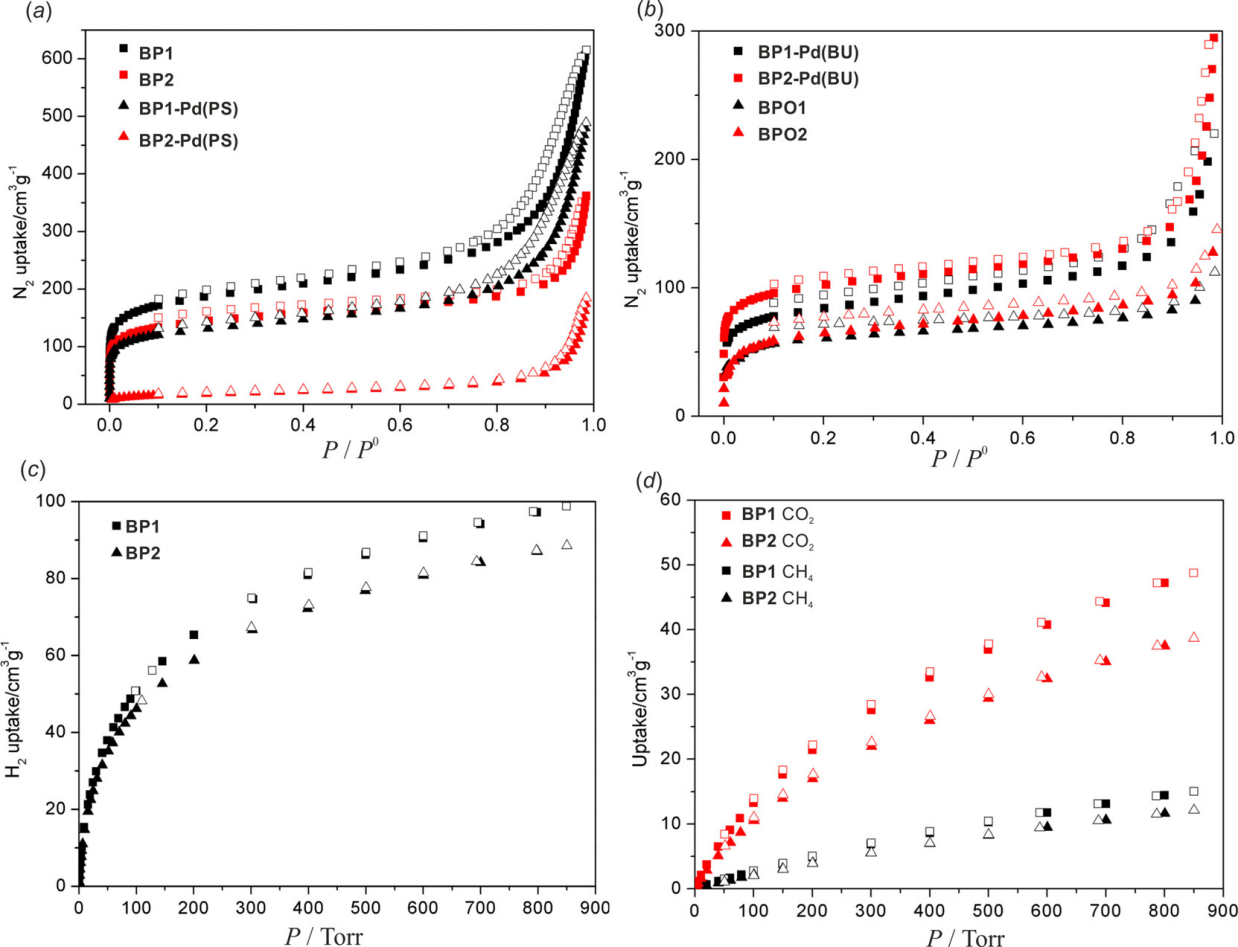
(*a*, *b*) N_2_ sorption isotherms (@77 K) for studied porous materials. (*c*) H_2_ (@77 K), (*d*) CO_2_ and CH_4_ (@273 K) sorption isotherms for **BP1**–**2**.

The porosity of all obtained materials was initially evaluated using N_2_ gas adsorption at 77 K. Prior to the measurements, samples were activated by heating at 150 °C under high vacuum (10^−3^ torr) for 24 hours to remove any possible guest molecules. All recorded isotherms show a sharp increase of N_2_ uptake at low relative pressures (below 0.02 *P/P*_0_) which is common for microporous materials (Figure 5 a, b) including classical boron COFs. All studied materials exhibit similar type‐II sorption isotherms with relatively slow and almost constant increase of sorption in the 0.05–0.8 *P/P*_0_ pressure range. **BP1** shows the highest N_2_ uptake reaching ca. 650 cm^3^ g^−1^ STP. The N_2_ sorption is significantly decreased (to ca. 200 cm^3^ g^−1^ STP at *P=P*_0_) for the **BP1‐Pd(BU)** hybrid material. On the other hand, both materials based on THDMA have similar N_2_ sorption properties (up to ca. 300 cm^3^ g^−1^ at *P=P*_0_) which suggests that the porosity is not significantly affected by introduction of Pd coordination centers. In addition, desorption measurements revealed that the isotherms are almost reversible for these two materials. For **BP1** and **BP1‐Pd(BU)**, a more distinctive hysteresis loop was observed which may indicate the occurrence of capillary condensation within mesopores, due to the strength of adsorbate‐adsorbent and adsorbate‐adsorbate interactions.^52^

The Brunauer–Emmett–Teller model was used to calculate the relevant parameters including apparent surface area *S*_BET_ based on the Rouquerol's consistency criteria,^53^ and the pore volume *V*_p_ at *P/P*_0_=0.98 (Table 2). The *S*_BET_ value for **BP1** was significantly higher (669 m^2^ g^−1^) than the remaining materials (72–532 m^2^ g^−1^). Obtained results are comparable with those reported for related boron 2D COFs such as COF‐1, COF‐5,^6^ and BTP‐COF.^54^

**Table 2:** Summary of surface areas, *S*_BET_, gas uptakes and total pore volumes, *V*_p_ for all studied COF materials.

	**BP1** ^[a]^	**BP2** ^[a]^	**BPO1**	**BPO2**	**BP1‐Pd(PS)**	**BP2‐Pd(PS)**	**BP1‐Pd(BU)**	**BP2‐Pd(BU)**
*S*_BET_ [m^2^ g^−1^]	669(703)	532(480)	235	245	485	71	310	383
*V*_p_ [cm^3^ g^−1^]	0.95(0.48)	0.56(0.55)	0.17	0.22	0.76	0.30	0.34	0.49
N_2_ @77 K, *P*/*P*_0_=0.98	615.1(310.6)	362.0(358.1)	112.2	145.4	489.2	184.2	220.0	316.5
H_2_@77 K, *P*=850 torr	98.7(111.5)	88.5(68.6)	64.3	77.9	86.3	57.5	68.6	77.9
CO_2_@273 K	48.7(54.7)	38.7(33.5)	32.0	33.2	36.9	21.6	32.6	33.7
CH_4_@273 K	15.0(16.7)	12.1(9.8)	9.8	10.5	14.7	7.8	10.1	10.9

[a] data for materials prepared at room temperature are given in parentheses.

We have also studied H_2_, CO_2_ and CH_4_ sorption for **BP1**–**2** (Figure 5 c,d) as we hypothesized that the presence of Lewis acidic (B atoms) and Lewis basic (P atom) centers may be beneficial for selectivity of obtained materials due to synergistic effects. Specifically, highly inhomogeneous electric field could enhance interactions with molecules featuring polar bonds such as CO_2_, but may also induce bond polarization in homoatomic molecules such H_2_. Both materials exhibit type‐I sorption of H_2_@77 K which is not saturated at *P=P*_0_. The H_2_ uptake for **BP1** is only slightly higher than for **BP2** (98.5 vs. 88.5 cm^3^ g^−1^ STP, *P*=850 torr) which implies a much higher H_2_ versus N_2_ sorption selectivity in favor of the latter material (Table 2). In case of **BP1‐Pd(PS)** and **BP2‐Pd(PS)** the N_2_ uptake was decreased. This effect was stronger for the latter material (485 cm^3^ g^−1^ STP for **BP1‐Pd(PS)** vs. 71 cm^3^ g^−1^ STP for **BP2‐Pd(PS)**). The sorption of H_2_ was also lowered (86.3 and 57.5 cm^3^ g^−1^ STP for **BP1‐Pd(PS)** and **BP2‐Pd(PS)**, respectively, *P*=850 torr) but to a lesser extent. This suggests the microporous structure was not strongly deteriorated by an introduction of Pd^0^ centers on the surface of original materials **BP1**–**2**.

Nitrogen sorption of hybrid materials **BP1‐Pd(BU)** and **BP2‐Pd(BU)** was much lower (ca. two‐fold) compared to their non‐palladium counterparts **BP1**–**2** (Table 2). However, H_2_ uptakes for **BP2** and **BP2‐Pd(BU)** are in fact comparable (88.5 vs. 77.9 cm^3^ g^−1^ STP, respectively, *P*=850 torr) which shows that introduction of Pd centers seems to be beneficial in terms of H_2_/N_2_ sorption selectivity.

Investigation of the sorption properties of **BPO1**‐**2** revealed that their N_2_ uptake at 77 K amounts to ca. 100 cm^3^ g^−1^ STP at *P*/*P*_0_=1, i.e., it was significantly lower compared to their analogues with P^III^ centers. The sorption of H_2_@77 K was also lower relative to **BP1**–**2** but the difference was much smaller by the factors of ca. 1.5 and 1.1 for **BPO1**–**2**, respectively (Figure S51). This is consistent with an increased H_2_/N_2_ uptake ratio for **BPO1**–**2** relative to **BP1**–**2**. This can be attributed to structural peculiarity due to the presence (and perhaps cooperation) of donor (P=O motif) and acceptor (B atom) sites.

The sorption of CO_2_ and CH_4_ was investigated at 273 and 293 K. For **BP1**–**2**, the sorption of CO_2_ is at a moderate level (ca. 50 and 30 cm^3^ g^−1^ STP @273 K at *P*=*P*_0_) and is much higher than for CH_4_ and N_2_ (Figure 5 d). The isosteric heats of adsorption of CO_2_ and CH_4_ for higher degrees (>0.3) of surface coverage equal to ca. 27 and 20 kJ mol^−1^, respectively, and thus they are comparable to values found for most COFs. The sorption of CO_2_ and CH_4_ were also studied with Pd‐functionalized COFs as well as **BPO1**–**2**. In all cases gas uptake was lower which can be ascribed to reduced porosity. Furthermore, coordination of Pd^0^ centers or oxidation of phosphorus centers did not result in a significant change of CO_2_/CH_4_ selectivity relative to **BP1**–**2**.

**Figure 6:**
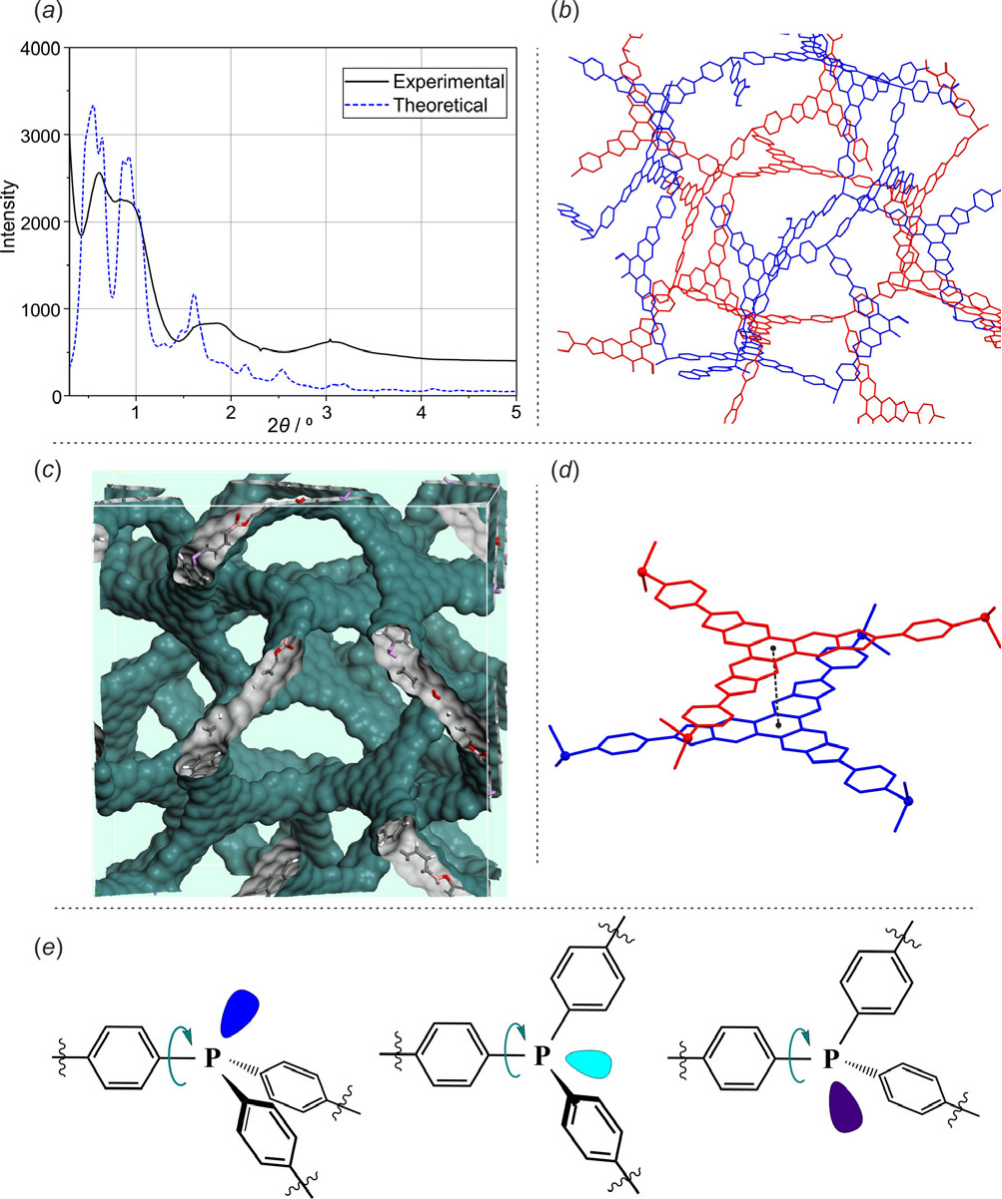
(*a*) Synchrotron‐measured (black solid line) and simulated (blue dashed line) PXRD pattern of **BP1**. (*b*) Proposed structural model for the **BP1** material. (*c*) Connolly surface generated in the Mercury program; (*d*) two neighbored polymeric fragments are related by the symmetry center; (*e*) rotation around C−P bond as a source of structural disorder.

Laboratory PXRD analyses of all obtained materials indicate their crystallinity is rather low. Nevertheless, we succeeded in performing such analyses for **BP1** COF using the synchrotron radiation (*λ*=0.178 Å). The PXRD pattern of **BP1** displays several broad peaks at lower 2θ angle (Figure 6). We proposed several structural models and compared the generated PXRD patterns to the experimental one. The best fit was obtained with a 3D structure resembling the topology of COF‐105 and COF‐108 obtained from condensation of tetrahedral precursors M(4‐B(OH)_2_Ph)_4_ (M=C, Si) and HHTP as a trigonal linker.^9^ However, in **BP1** 1/4 of connections are replaced by the lone electron pair of the phosphorus atom. This partially resembles the situation observed in COF materials reported by Dichtel and Bunck by co‐condensation of tetrahedral C(4‐B(OH)_2_Ph)_4_ and truncated CR(4‐B(OH)_2_Ph)_3_ (R=*n*‐C_12_H_26_, allyl).^55^ In our case, the structure is even more labile and supposedly vulnerable for catenation. In our model we proposed a two‐fold interpenetration level, where two neighboring networks are related by the inversion center. However, a higher degree of catenation can also be considered. The PXRD pattern of **BP2** also shows some degree of structural ordering (Figure S37) which indicates that both materials can be categorized as COFs. We note, however, the proposed model should properly describe only the short‐range order in this material. It seems the structures **BP1** and **BP2** are not strictly defined due to variable orientation of phosphorus centers leading to significant disorder and lack of well‐defined long‐range order.

In order to study the specificity of host‐guest interactions in obtained materials, we have performed series of quantum chemical calculations at M062X/cc‐pVTZ level of theory.^56^, ^57^ To simplify the calculations, the networks of **BP1** and **BPO1** were reduced to two closest fragments comprising organoboron and organophosphorus monomers with the initial distance between boron and P or P=O oxygen atoms set to 2.4 Å (**BP1’** and **BPO1’**). In the case of **BP1’** the geometry optimization led to the increase of the B…P distance to about 3.5 Å, meaning that moderate compatibility of phosphorus LP and boron free *p* orbital is insufficient to overcome the steric hindrance. In turn, in **BPO1’** the P=O…B distance was shortened to 2.11 Å resulting in appearance of dative P=O→B bond and slight pyramidalization of geometry around boron atom. The calculated interaction energy of 15.5 kJ mol^−1^ suggests that BPOs would exhibit tendency for additional interconnections through the dative P=O→B interactions, but this effect seems to be too weak to organize the material into the well‐defined crystal samples. Conversely, such a labile bond would rather decrease the ordering level.

Since polymer networks of **BP1** and **BPO1** are considered labile, it can be expected that Lewis base and acid centers would appear in the appropriate distance to invoke the local electric field effect, which could then enhance the interactions with guest molecules. The electrostatic potential maps plotted on electron density isosurfaces show strong electronegative regions around the phosphorus (**BP1’**) or P=O oxygen (**BPO1’**) atoms, while the slightly positive regions appear above the planes of boronate groups. (Figure S86). Due to rather low Lewis acidity of the boron atom, the expected binding effect would be rather weak and unsymmetrical with respect to classical frustrated Lewis pairs (FLPs).^58^ It would be mostly dominated by the interaction with P^III^/P=O donor, and only supported by the interaction with boron atom. Indeed, the performed calculations on the host‐guest systems confirms that the presence of boron and phosphorus centers supports binding of H_2_, CH_4_ and CO_2_ molecules by the framework, but this effect is insufficient to promote heterolytic bond splitting. The computed interaction energies clearly show that **BPO1’** displays higher affinity toward H_2_ and CH_4_ molecules, while **BP1’** strongly interacts (*ΔE*=−33.4 kJ mol^−1^) with CO_2_ molecule. In line with these observations, the P/O⋅⋅⋅H (H_2_, CH_4_) distances are shorter in **BPO1’** and P=O⋅⋅⋅CO_2_ distance is longer with respect to P⋅⋅⋅CO_2_ in **BP1’** (Table 3). This can be simply understand by the higher basicity of oxygen atom, and stronger nucleophilic character of phosphorus atom.

**Table 3:** Characterization of host‐guest interaction energies. Δ*E* denotes the interaction energy between host and guest molecule, *d* is interatomic distance between P/O, B and guest molecule XY (X,Y=H for H_2_, CH_4_; X=C and Y=O for CO_2_), *ρ* and $\nabla^{2}$ *ρ* are electron density and its Laplacian at P/O…X (BPC1) and Y…B (BCP2) bond critical points.

Host	Guest	Δ*E* [kJ mol^−1^]	*d*_P/O⋅⋅⋅X_ [Å]	*d*_B⋅⋅⋅Y_ [Å]	*d*_P/O⋅⋅⋅B_ [Å]	*ρ*(BCP1) [eÅ^−3^]	▿^2^ *ρ*(BCP1) [eÅ^−5^]	*ρ*(BCP2) [eÅ^−3^]	▿^2^ *ρ*(BCP2) [eÅ^−5^]
**BP1**	H_2_	−1.1	2.503	2.273	4.850	0.12	1.02	0.09	0.94
	CO_2_	−33.4	2.393	3.155	6.014	0.41	0.80	0.03	0.46
	CH_4_	−0.8	2.835	2.426	6.483	0.07	0.57	0.07	0.69
**BPO1**	H_2_	−5.7	1.963	2.101	4.458	0.16	2.34	0.11	1.09
	CO_2_	−6.3	2.437	2.526	5.186	0.14	2.23	0.11	1.19
	CH_4_	−4.2	2.556	2.933	6.306	0.05	0.56	0.03^[a]^	0.33^[a]^

[a] BCP2 was found between C−H hydrogen and boronate ester oxygen atom.

**Figure 7:**
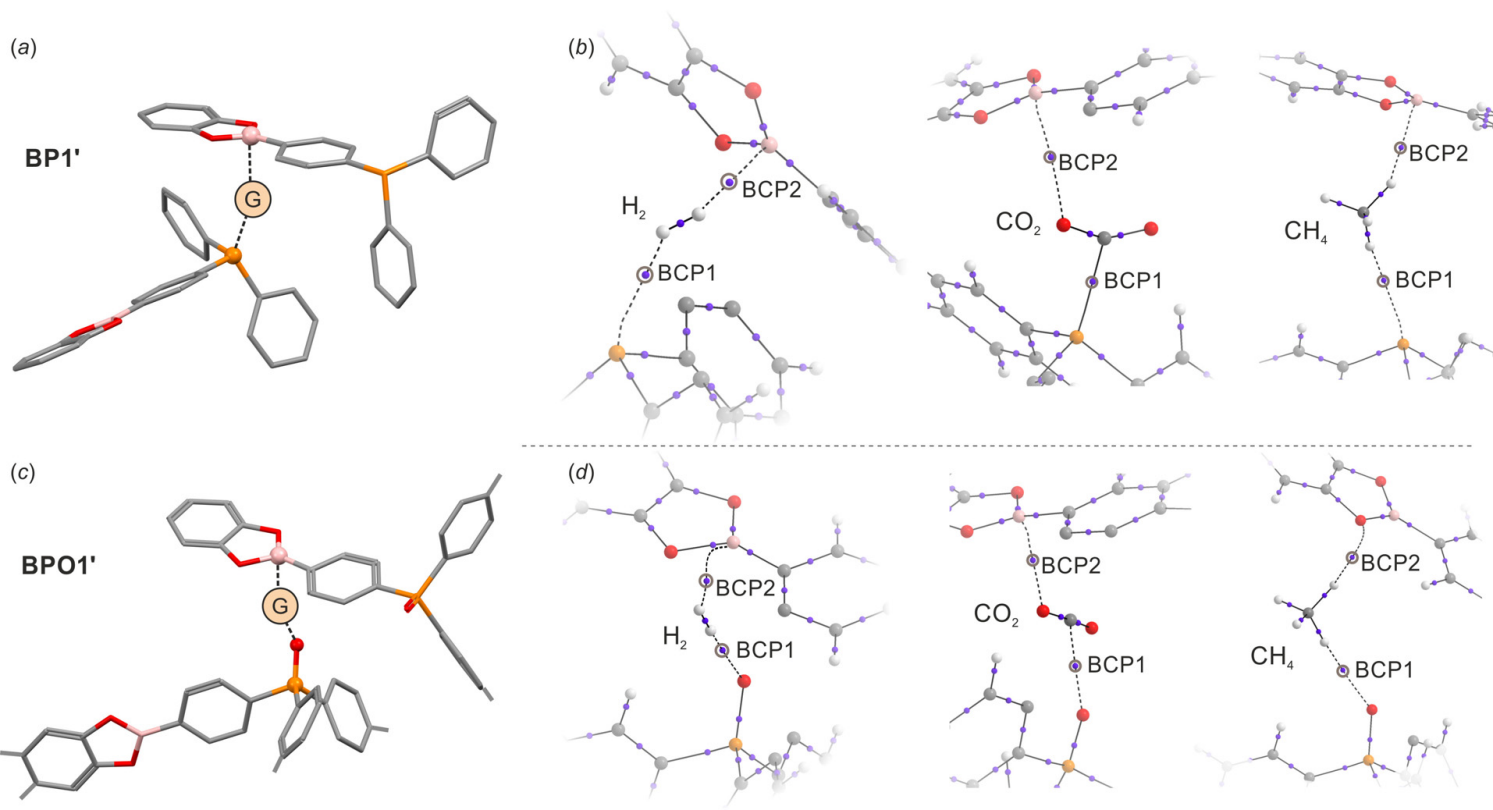
(*a*,*c*) Modelling of host‐guest interactions in **BP1** and **BPO1** materials. (*b*,*d*) Fragment of molecular graphs showing bond paths (black dashed lines) and bond critical points (small blue spheres) in host‐guest interaction region.

The deeper insight into the host‐guest binding mechanism is provided by the topological analysis of electron density in the framework of Bader's quantum theory of atoms in molecules (QTAIM).^59^ Table 3 gathers the most important geometrical and electron density topological features of host‐guest interactions. Molecular graphs showing the formation of bond paths and bond critical points (BCP) in the binding pocket are presented on Figure 7, full molecular graphs are given in SI (Figures S87**–**S92). The topological analyses of electron density recognized bond paths and bond critical points (BCPs) between all donor centers and H (H_2_, CH_4_) or C (CO_2_) atoms from guest molecules. The electron density values follows the general trend observed for interatomic distances. Specifically, for **BPO1’**‐H_2_ adduct, where donor…H distance is the shortest from studied series (*d*_O….H_=1.963 Å), the electron density at P⋅⋅⋅H bond critical point reaches the value of 0.16 eÅ^−3^ with negative Laplacian of 2.34 eÅ^−5^ and host‐guest interaction energy equals to −5.7 kJ mol^−1^ indicating that this interaction can be classified as weak hydrogen bond. As indicated by longer P⋅⋅⋅H distance (2.503 Å) and lower *ρ*(BCP1) value of 0.12 eÅ^−3^, the binding of dihydrogen by **BP1’** is weaker (*ΔE*=−1.1 kJ mol^−1^), but still satisfies the criteria of very weak intermolecular hydrogen bond. In both cases, the donation from donor to H_2_ molecule results in elongation of H−H bond from 0.744 Å (equilibrium distance in free H_2_ calculated at the same level of theory) to 0.761 Å in **BP1’** and 0.780 Å in **BPO1’**, which is accompanied by the reduction of electron density at H‐H BCP from 1.84 eÅ^−3^ to 1.81 eÅ^−3^ (**BP1’**) and 1.68 eÅ^−3^ (**BPO1’**). Our observations are somewhat consistent with theoretical studies conducted by Pinter et al.^60^ on FLPs featuring low‐energy dihydrogen activation transition states termed “early” (such as *t*Bu_3_P⋅B(C_6_F_5_)_3_). In contrast to so‐called “late” FLPs, such “early” complexes are characterized by relatively short H‐H distances (0.79–0.80 Å), slightly decreased electron density at H_2_ BCP (1.6 eÅ^−3^), long P⋅⋅⋅H distances and electron density values at BCPs of P⋅⋅⋅H bond in the range of 0.2–0.3 eÅ^−3^. Nonetheless, “early” FLPs systems are active hydrogenation catalysts. The comparison between FLPs and our systems leads to the conclusion that in the latter the donor binding effect is weaker presumably due to the lack of strong support from acceptor side. Although, topological analysis of electron density revealed the formation of H…B bond path in both models **BP1’** and **BPO1’**, the value of electron density at BCP2 oscillates near 0.1 eÅ^−3^ and it is more than two times smaller with respect to “early” *t*Bu_3_P⋅H_2_⋅B(C_6_F_5_)_3_ complex. On the other hand, the lower activity of **BP1’** and **BPO1’** is compensated by the higher stability of formed host‐guest adducts.

An interesting observation can be made by the comparison of interatomic distances and electron density features of B⋅⋅⋅H versus donor P^III^/P=O⋅⋅⋅H interactions with dihydrogen and methane. Despite the C−H bond polarization, the donor⋅⋅⋅H interactions are weaker with methane than dihydrogen. It should be also noted that in case of the **BPO1’**‐CH_4_ bond path was recognized between C−H hydrogen atom and oxygen atom from boronate ester group with electron density of only 0.03 eÅ^−3^ at BCP2. Thus the contribution from boron atom is questionable. Regardless of binding mechanism, the C−H bonds from donor and acceptor sites are elongated and electron density depleted at BCP with respect to free CH_4_ molecule. Furthermore, the calculations show that both H_2_ and CH_4_ adducts are unstable in the absence of boron counterparts.

The **BP1’**‐CO_2_ is the most distinct adduct from the studied series. The exceptionally high value of electron density at P⋅⋅⋅CO_2_ BCP of 0.46 eÅ^−3^ confirms the strong character of this interaction and points to the chemical nature of CO_2_ adsorption. This also leads to the significant bending of CO_2_ molecule (α_O‐C‐O_=153°), elongation of C−O bond (from 1.155 Å to 1.178 Å), and reduction of electron density at corresponding BCP (from to 3.158 eÅ^−3^ to 3.038 eÅ^−3^) with respect to free CO_2_ molecule. On the other hand, the boron atom is barely involved. Turning to less nucleophilic phosphine oxide, the interaction energy with CO_2_ (*ΔE*=−6.3 kJ mol^−1^) and the amount of electron density localized at BCP1 (0.14 eÅ^−3^) are comparable to **BPO1’**‐H_2_ adduct. Accordingly, the geometry of CO_2_ molecule approach to linear with the O‐C‐O angle of 171°. Interestingly, the formation of moderate P=O⋅⋅⋅CO_2_ interaction is accompanied by the increased contribution from boron atom reflected by relatively short B⋅⋅⋅O contact (2.526 Å) and electron density value of 0.11 eÅ^−3^ at BCP2.

The analysis of Hirshfeld^61^ and Bader^59^ atomic charges revealed that the charge is transferred from donor to guest molecule, however, it is only partially transferred further to acceptor unit (Tables S2 and S3). For most systems, the population at the donor atom (P^III^/P=O) drops by 0.02–0.04 e (Hirshfeld charges), while the magnitude of charge donation from the guest molecule to the boron atom is of about 0.005–0.025 e. In line with previous findings, the magnitude of charge transfer from **BP1’** donor to CO_2_ molecule is larger. The charge at P atom is depleted by ca. 0.1 e, while guest molecule and boron atom increase electron population by 0.162 e and 0.025 e, respectively. It is also noticeable that charge is distributed unsymmetrically within the guest molecule, wherein it is mostly shifted toward the acceptor hydrogen (H_2_, CH_4_) or carbon (CO_2_) atoms. This again emphasizes the dominant character of P^III^/P=O donor site in the binding processes.

## Conclusions

We have demonstrated that the general approach to phosphine coordination materials mostly developed for MOF family can be successfully extended to covalent organic frameworks. The transesterification of triboronated triphenylphosphine **2** and its oxide **4** esters with polyhydroxy HHTP and THDMA linkers is a facile route toward the formation of boron‐phosphine COFs that are characterized by higher selectivity toward H_2_, CH_4_ and CO_2_. The tripodal topology of the employed boronic linkers stems from the presence of the phosphorus(III) center. Obtained materials display lower crystallinity with respect to related boron COF materials resulting from fast transesterification rates and statistically random orientation of phosphorus center with respect to three aryl substituents and lone electron pair. However, synchrotron radiation experiments revealed some short‐range structure ordering. The proposed **BP1** structural model assumes two‐fold interpenetration level and general topology preserved from COF‐105, but it is characterized by higher flexibility and ordering discontinuity.

The BET surfaces derived from nitrogen sorption isotherm are moderate. However, obtained materials exhibit uptakes of hydrogen, CO_2_ and methane at a level similar to those found for boron COFs featuring higher N_2_‐based *S*_BET_ values. In other words, the obtained porous materials have much higher relative affinity with respect to H_2_, CO_2_ and CH_4_ than standard boron COFs. We suppose that this effect can be ascribed to the presence of boron Lewis acidic and phosphorus Lewis basic centers which is beneficial for binding of more polarizable molecules due to generation of local electric field gradients. Theses suppositions has been confirmed by theoretical calculations. The interaction with gaseous molecules has some common features with “early” FLP systems reflected by slightly decreased distance and electron density at BCP of H−H bond, elongated donor⋅⋅⋅guest distances with electron density localized at corresponding BPC ranging from 0.2–0.3 eÅ^−3^ and 0.02–0.04 e charge transferred from basic center to guest molecule. On the other hand the contribution of Lewis acidic boron atom is less pronounced. Thus, an overall effect is beneficial for sorption effectiveness but it is insufficient to promote stronger host‐guest interactions or bond splitting. In contrast, theoretical calculations suggest that CO_2_ is bound by **BP1** more effectively due to stronger nucleophilic character of P^III^ atom and intrinsic polarity of C=O bonds.

Post‐synthetic modification of the materials was performed by impregnation with Pd_2_(dba)_3_ in DCM. XPS analyses demonstrated that the presence of phosphorus donor centers results in a strong affinity to Pd^0^ leading to the high Pd/P ratio of ca. 0.9. Incorporation of the metal resulted in significant decrease of the BET surface. However, there is still space left available for uptake of guest molecules. In a different bottom‐up approach, the precursor **1** was first reacted with Pd_2_(dba)_3_ followed by polycondensation with HHTP or THDMA. Thus the resulting materials possess the hybrid character due to presence of Pd−P dative bonds as well as boronate ester moieties typical of boron COFs. The sorption properties of these amorphous networks are slightly worse than those found for **BP1**–**2**. According to XPS analyses, the obtained materials exhibit much lower surface Pd/P ratio of 0.26–0.31 consistent with the assumed stoichiometry of the Pd complex used as a precursor. All obtained Pd‐containing porous materials could potentially serve as heterogeneous catalysts, e.g., for hydrogenation and cross‐coupling reactions. We will test such applications in our future approaches.

## Conflict of interest

The authors declare no conflict of interest.

## Supporting information

As a service to our authors and readers, this journal provides supporting information supplied by the authors. Such materials are peer reviewed and may be re‐organized for online delivery, but are not copy‐edited or typeset. Technical support issues arising from supporting information (other than missing files) should be addressed to the authors.

Supplementary
