# Supplementary material for: Boronate Covalent and Hybrid Organic Frameworks Featuring PIII and P=O Lewis Base Sites
Source: Chemistry. 2020 Sep 1;26(56):12758–68. doi: 10.1002/chem.202001960 (PMC7589431; doi:10.1002/chem.202001960)
Supplement: Supplementary file 1 — Supplementary [file CHEM-26-12758-s001.pdf]

Chemistry–A European Journal

Supporting Information

Boronate Covalent and Hybrid Organic Frameworks Featuring P^{III} and P=O Lewis Base Sites

Piotr Pacholak,[a] Krzysztof Gontarczyk,[a] Radosław Kamiński,[b] Krzysztof Durka,*[a] and Sergiusz Luliński*[a]

Table of content

1. **Synthesis** S2
2. **TGA analysis** S6
3. **NMR spectra** S7
4. **MAS NMR spectra** S18
6. **PXRD** S27
7. **SEM** S27
8. **Single-crystal X-ray diffraction analysis of 2** S29
9. **Sorption measurements** S29
10. **Computational studies** S53
11. **References for Supporting Information** S64

1. Synthesis

General comments. THF, 1,4-dioxane, mesitylene used for reactions were distilled from sodium/benzophenone ketyl under argon and stored over 4Å molecular sieves. Anhydrous dichloromethane, pentane, hexane and heptane were obtained from a commercial source. Starting materials including 1,4–dibromobenzene, PCl_3, trialkyl borates, Me_3SiCl, HHTP, and Pd_2dba_3 were used as received without further purification. THDMA was prepared as described previously.[1–3]

Synthesis of boronated precursors.

Tris(4–bromophenyl)phosphine (1).[4] A solution of 1,4–dibromobenzene (117.9 g, 0.5 mol, 3.0 equiv) in a mixture of Et_2O (540 mL) and THF (180 mL) was cooled to –78 °C under argon atmosphere, and then 10 M *n*-BuLi (50 mL, 0.5 mol, 3.0 equiv) diluted with hexane (50 mL) was added dropwise for 1 h. The resulting white suspension was stirred for 1 hr at –78 °C. Then PCl_3 (14.8 mL, 0.17 mol, 1.0 equiv) diluted with hexane (20 mL) was added dropwise for 1 hr at –78 °C. The mixture was stirred for 16 h, while it was allowed to warm to room temperature. A resulting white suspension was hydrolyzed with brine (200 mL). The organic phase was separated, extracted with Et_2O (2 × 200 mL). The combined organic solutions were dried over $MgSO_4$, filtered and evaporated to dryness. Obtained solid was recrystallized under argon atmosphere in MeOH (1.9 L) and PhMe (230 mL) to give a white solid, m.p. 117-118 °C. Yield: 53.4 g (63%). ^{1}H NMR (400 MHz, acetone) δ 7.61 (td, 6H), 7.24 (td, J = 7.3, 0.7 Hz, 6H) ppm. ^{31}P NMR (162 MHz, acetone) δ –8.7 ppm.

Tris(4–diisopropoxyborylphenyl)phosphine (2). A solution of **1** (17.4 g, 0.035 mol, 1.0 equiv), B(O*i*Pr)$_3$ (22.0 mL, 0.126 mol, 3.6 equiv) in anhydrous THF (350 mL) was cooled to –78 °C under argon atmosphere, and then *t*-BuLi (124 mL, 1.7 M, 0.21 mol, 6.0 equiv) was added dropwise for 40 min. The mixture was stirred for 18 h at –78 °C. Then Me_3SiCl (28.0 mL, 0.224 mol, 6.4 equiv) was added during 5 min and the mixture was allowed to warm to an ambient temperature. Solvents were evaporated to dryness under reduce pressure and obtained solid was triturated with anhydrous heptane (50 mL). A clear colorless organic phase containing the product was carefully removed with a syringe and left to stand for 2 days at –20 °C. The solution was decanted from over precipitated white crystalline solid and concentrated to ca. one third of initial volume followed by cooling at –20 °C for 24 hrs. This resulted in the second batch which was dried under high vacuum to give 1.8 g of the product. The collected materials were combined and dried under high vacuum to give the product. Yield: 11.3 g (50%). *Caution: all operations should be carried out under argon atmosphere as the product is readily hydrolyzed in moist air.* ^{1}H NMR (300 MHz, $CDCl_3$) δ 7.57-7.50 (m, 6H), 7.37-7.29 (m, 6H), 4.65-4.56 (m, 6H), 1.24 (d, J = 6.1 Hz, 36H) ppm. ^{11}B NMR (96 MHz, $CDCl_3$) δ 27.5 ppm. ^{13}C NMR (75 MHz, $CDCl_3$) δ 138.1 (d, J = 10.8 Hz), 133.2, 133.1-132.8 (m), 66.3, 24.7 ppm. ^{31}P{^{1}H} NMR (122 MHz, $CDCl_3$) δ –5.4 ppm.

Tris(4–bromophenyl)phosphine oxide (3).[4,5] To a solution of **1** (9.88 g, 0.02 mol) in CH_2Cl_2 (50 mL), aq. H_2O_2 30wt%, 20 mL was added. The obtained mixture was stirred overnight and then organic layer was separated, washed with brine and evaporated under reduced pressure. To obtained residue hexane (10 mL) was added and product was filtered. Yield: 10.13 g (99%). ^{1}H NMR (300 MHz, $CDCl_3$) δ 7.74-7.61 (m, 6H), 7.60-7.43 (m, 6H). ^{31}P NMR (122 MHz, $CDCl_3$) δ 27.6.

Tris(4–diisopropoxyborylphenyl)phosphine oxide (4). This compound was obtained using the protocol described for **2** using tris(4-bromophenyl)phosphine oxide **3** (5.00 g, 9.71 mmol, 1 equiv), *t*-BuLi (36.00 mL, 61.2 mmol, 6.3 equiv), B(O*i*Pr)$_3$ (6.00 mL, 35.0 mmol, 3.6 equiv) and Me_3SiCl (8.00 mL, 62.1 mmol, 6.4 equiv) as the starting materials. It was obtained as a yellow powder. Yield (2.23 g, 35%). ^{1}H NMR (300 MHz, $CDCl_3$) δ 7.67-7.50 (m, 12H), 4.65-4.56 (m, 6H), 1.24 (d, J = 6.1 Hz, 36H) ppm. ^{13}C NMR (101 MHz, $CDCl_3$) δ 134.5, 132.5 (d, J = 12.0 Hz), 131.4 (d, J = 10.0 Hz), 130.9, 66.5, 24.6 ppm. ^{11}B NMR (96 MHz, $CDCl_3$) δ 28.5 ppm. ^{31}P{^{1}H} NMR (122 MHz, $CDCl_3$) δ 30.5 ppm.

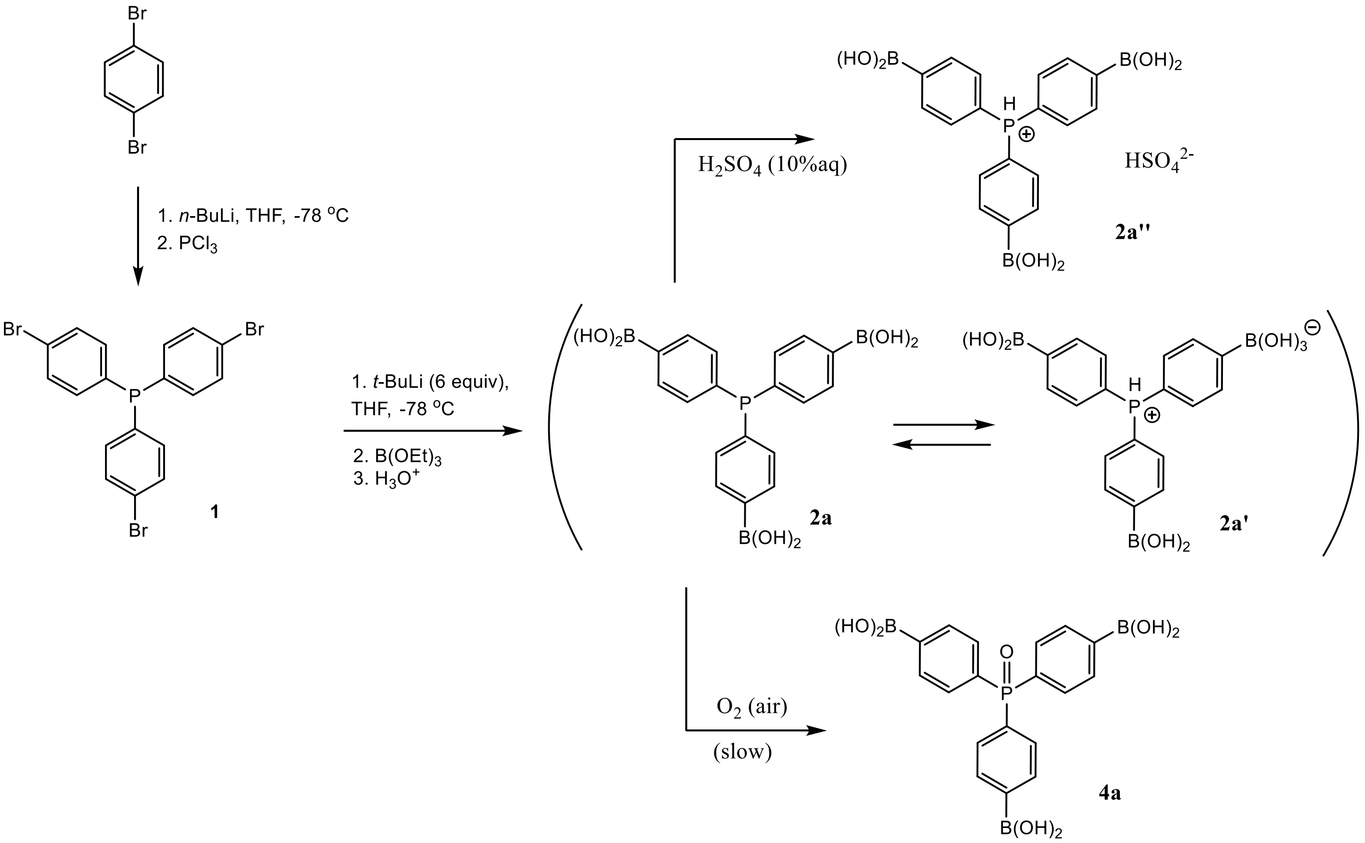

Scheme S1. Initial attempts for the synthesis of boronated precursor **2a**. Obtained boronic acid equilibrates with its zwitterionic form **2a'**. Boronic acid can be isolated in the form sulfate salt **2a''**, however, the solubility of this compound is very low. **2a** is prone to oxidation when stored under air - the formation of corresponding phosphine oxide was observed (**4a**).

Synthesis of COFs.

BP1. Compound **2** (0.99 g, 1.53 mmol, 1 equiv) and HHTP (0.50 g, 1.53 mmol, 1 equiv) were placed into a 150 mL Schlenk flask. Mesitylene (38 mL) and 1,4-dioxane (38 mL) were added and the mixture was degassed under reduced pressure. Then the mixture was heated at 85 °C with stirring for 72 hours. The obtained suspension was filtered under argon atmosphere. THF (50 mL) was added and the mixture was stirred overnight. The washing with THF was repeated twice in the same manner. The obtained slurry was dried *in vacuo* at 85 °C for 24 hours to afford the product as a grey powder. Final removal of remaining guest molecules was accomplished by heating at 150 °C under high vacuum (< $5\cdot10^{-4}$ Torr) for 24 hours. Yield 0.67 g (71%). ^{1}H NMR spectrum of a hydrolyzed sample (300 MHz, DMSO) δ 8.06 (dd, *J* = 7.9, 2.9 Hz, 2H), 7.92 (d, *J* = 6.7 Hz, 2H), 7.78 (s, 1H, HHTP), 7.56 (d, *J* = 3.6 Hz, 2H), 7.35 (t, *J* = 7.8 Hz, 2H), 3.65 (s, 1H) ppm. ^{31}P NMR (122 MHz, DMSO) δ = –6.3 ppm. ^{11}B MAS NMR (160.5 MHz, spinning rate 10 kHz) δ 23.8 ppm. ^{31}P MAS NMR (202.5 MHz, spinning rate 10 kHz) δ –0.2 ppm.

BP2. It was obtained as a dark green powder using the method described for **BP1** from **2** (1.055 g, 1.63 mmol, 1 equiv) and THDMA (0.66 g, 2.45 mmol, 1.5 equiv). Yield (0.91 g, 80%). ^{1}H NMR of a hydrolyzed sample (300 MHz, DMSO) δ 7.92 (dd, *J* = 7.8, 1.3 Hz, 2H), 7.50 (s, 1H), 7.36 (t, *J* = 7.9 Hz, 2H), 3.59 (s, 1H) ppm. ^{31}P NMR (122 MHz, DMSO) δ –7.2 ppm. ^{11}B MAS NMR (160.5 MHz, spinning rate 10 kHz) δ 24.4 ppm. ^{31}P MAS NMR (202.5 MHz, spinning rate 10 kHz) δ 0.3 ppm.

BPO1. It was obtained as a grey powder using the method described for **BP1** from **4** (0.51 g, 0.77 mmol, 1 equiv) and HHTP (0.25 g, 0.77 mmol, 1 equiv). Yield 0.400 g (83%). ^{11}B MAS NMR (160.5 MHz, spinning rate 10 kHz) δ 23.1, 13.7, 8.1 ppm. ^{31}P MAS NMR (202.5 MHz, spinning rate 10 kHz) δ 34.6 ppm.

BPO2. It was obtained as a dark green powder using the method described for **BP1** from **4** (0.60 g, 0.91 mmol, 1 equiv) and THDMA (0.37 g, 1.36 mmol, 1.5 equiv). Yield 0.520 g (81%).^{11}B MAS NMR (160.5 MHz, spinning rate 10 kHz) δ 21.8, 13.7, 8.4 ppm. ^{31}P MAS NMR (202.5 MHz, spinning rate 10 kHz) δ 34.6 ppm.

BP1-Pd(BU). Compound **2** (0.9 g) was dissolved in THF (10 mL). It was cooled to –78°C with stirring and a solution of Pd_2dba_3 (60 mg) in DCM (40 mL) was added with a syringe. The cooling bath was removed and the intense purple-violet solution was allowed to warm to an ambient temperature which was accompanied by gradual change in color to olive-brown. The solution was stirred for another 30 min and then cooled again to –78 °C and a solution of HHTP (0.5 g) in THF (10 mL) was added. The resulting solution was allowed to warm to an ambient temperature. The precipitation of a solid was observed after ca. 30 min whereas after next 3-4 hrs a very thick slurry was formed. It was stirred for 2 days and then it was filtered under argon atmosphere and washed thoroughly with DCM (4 × 30 mL). The pale yellow filtrate was evaporated to dryness and the remaining solid was found to be essentially pure dba ligand. The remaining solvent was removed from the filter cake under reduced pressure

and the product was dried under high vacuum at 50°C. Yield: 0.9 g. ^{11}B MAS NMR (160.5 MHz, spinning rate 10 kHz) δ 21.8, 7.2 ppm. ^{31}P MAS NMR (202.5 MHz, spinning rate 10 kHz) δ 34.5, –2.1 ppm.

BP2-Pd(BU): A protocol described for **BP1-Pd(BU)** was applied for **2** (0.52 g), Pd_2dba_3 (40 mg), and THDMA (0.3 g). The material was isolated as an olive-green powder. Yield: 0.60 g. ^{11}B MAS NMR (160.5 MHz, spinning rate 10 kHz) δ 22.0, 13.4, 6.8 ppm. ^{31}P MAS NMR (202.5 MHz, spinning rate 10 kHz) δ 36.2, –0.7 ppm.

BP1-Pd(PS). A sample of **BP1** (225 mg) was suspended in THF (10 mL). Then a solution of Pd_2dba_3 (60 mg) in THF (40 mL) was added. The mixture was stirred for 24 hrs and then it was left to stand for 8 h. The purple-violet solution containing unreacted Pd_2dba_3 was removed with a syringe and the remaining precipitate was repeatedly washed with THF until the supernatant was almost colorless (3 × 30 mL). Then, the rest of solvent was removed under reduced pressure followed by drying of the product under high vacuum (< $5 \cdot 10^{-4}$ Torr) at 50 °C for 12 hrs. It was obtained as greyish-green powder. Yield: 205 mg.

BP2-Pd(PS). A protocol described for **BP1-Pd(PS)** was applied for **BP2** (210 mg) and Pd_2dba_3 (40 mg). The material was isolated as an olive-green powder. Yield: 190 mg.

2. TGA analysis

Thermogravimetric analysis were performed on a TGA/DSC1 (Mettler-Toledo) system under continuous flow of argon at the ramp rate of 10 K min^{-1} from 30 °C to 600 °C. The samples were prepared in covered ceramic crucibles. An empty crucible was used as a reference. α-Al_2O_3 was used for instrument calibration. Samples were measured after preliminary drying at 85 °C under 0.1 Torr for 24 hours.

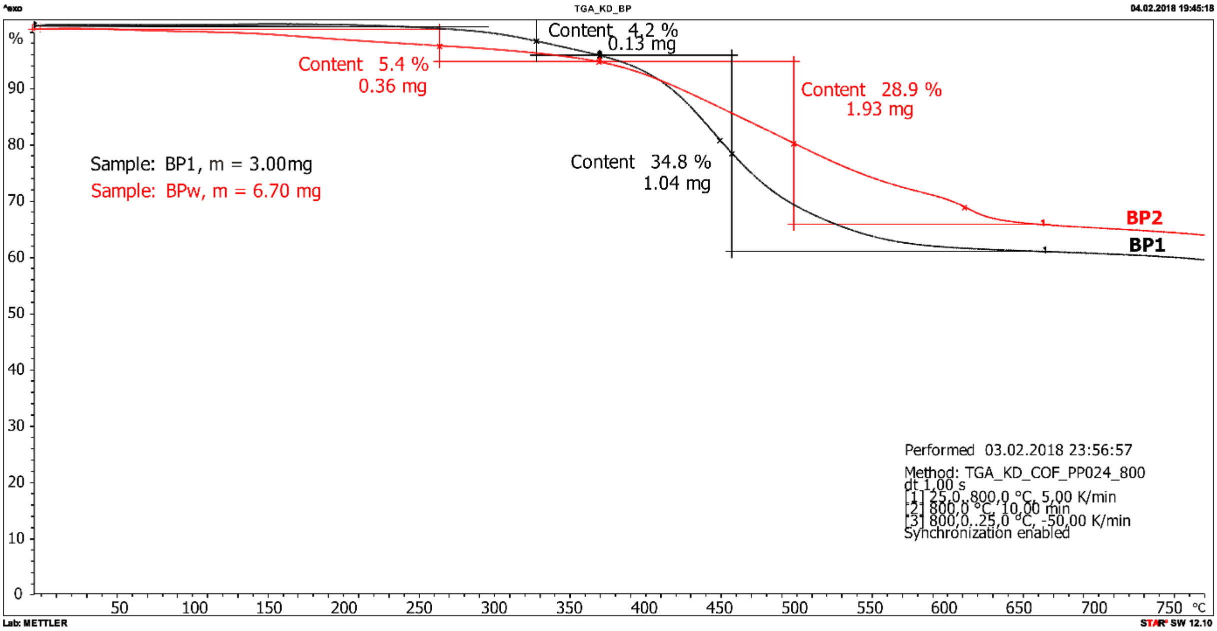

Figure S1. TGA analysis of **BP1** and **BP2**.

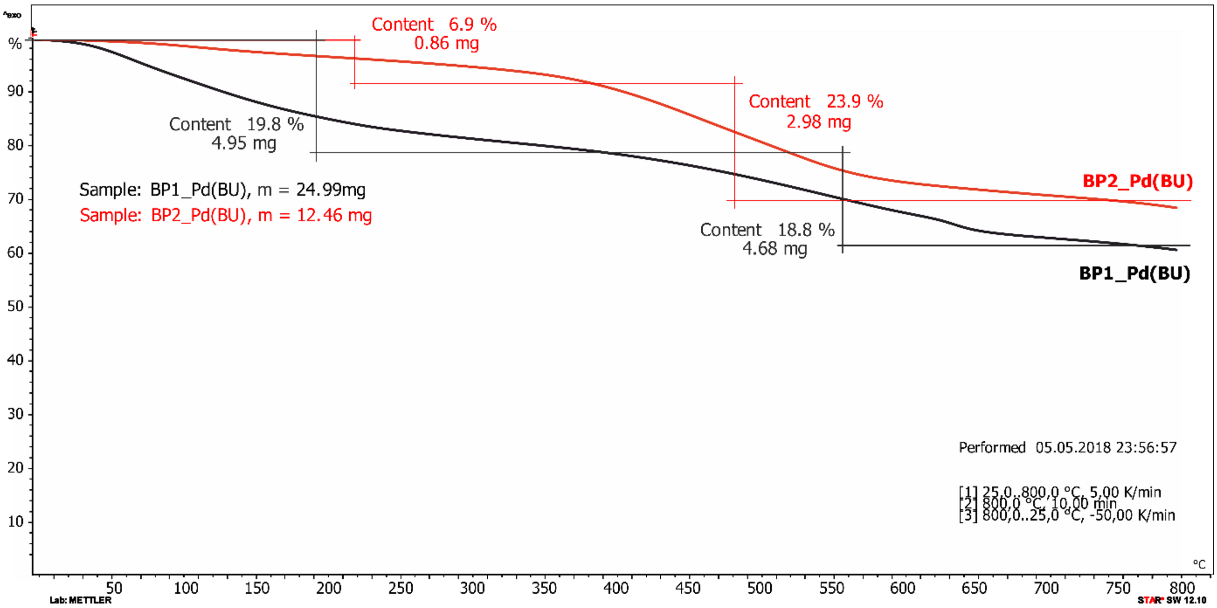

Figure S2. TGA analysis of **BP1-Pd(BU)** and **BP2-Pd(BU)**.

3. NMR spectra

^{1}H, ^{11}B, ^{13}C and ^{31}P NMR spectra were recorded on an Agilent NMR 400 MHz DDR2 spectrometer. In the ^{13}C NMR spectra the resonances of boron-bound carbon atoms were not observed in most cases as a result of their broadening by a quadrupolar boron nucleus. ^{1}H and ^{13}C chemical shifts are reported in ppm from TMS with the residual solvent resonances as internal standards. ^{11}B and ^{31}P NMR chemical shifts are given relative to $BF_3{\cdot}Et_2O$ and 85% phosphoric acid solution in D_2O, respectively.

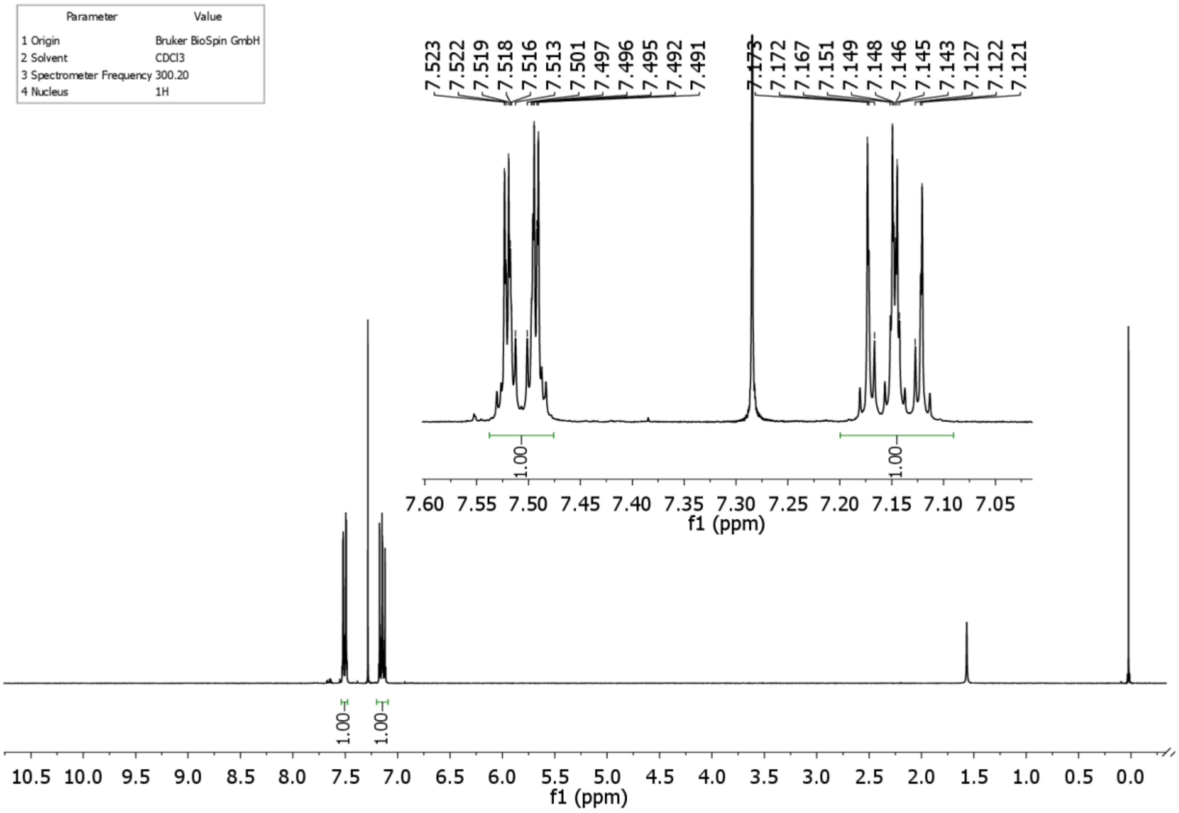

Figure S3. ^{1}H NMR spectrum of **1** in $CDCl_3$.

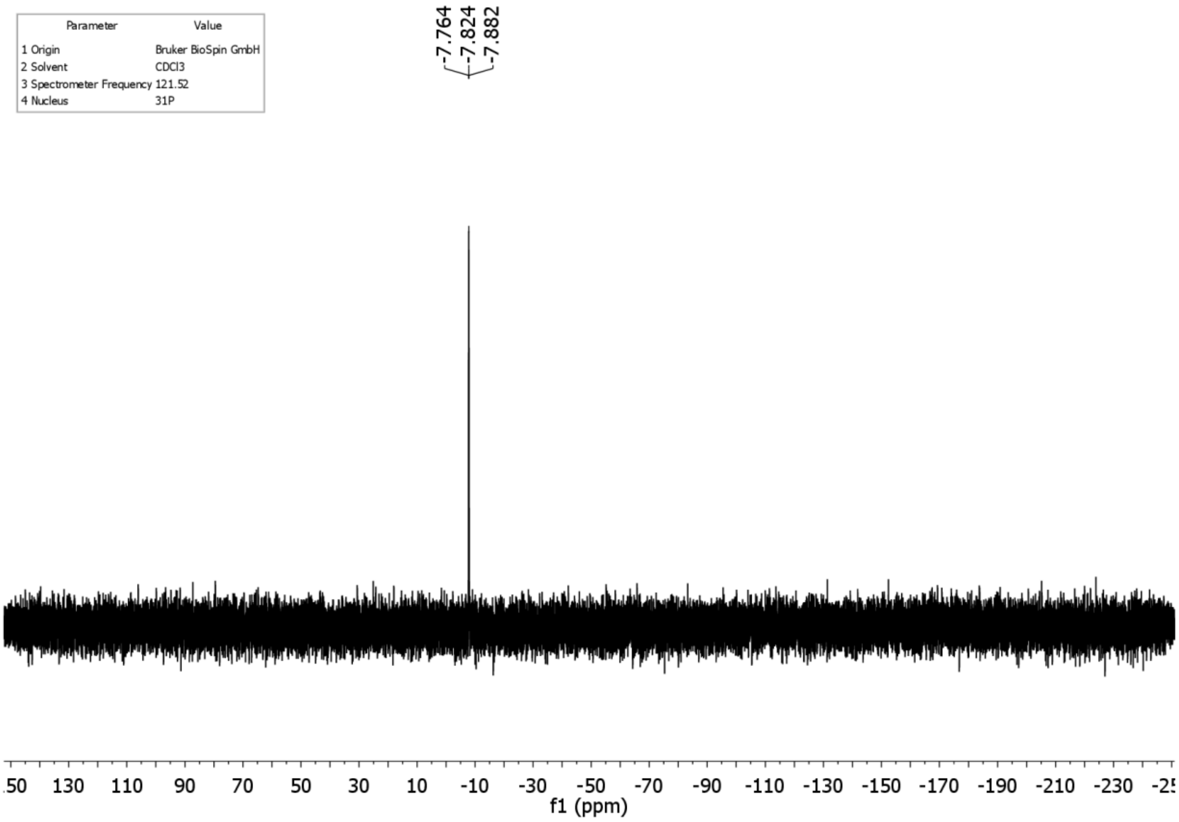

Figure S4. ^{31}P NMR spectrum of **1** in $CDCl_3$.

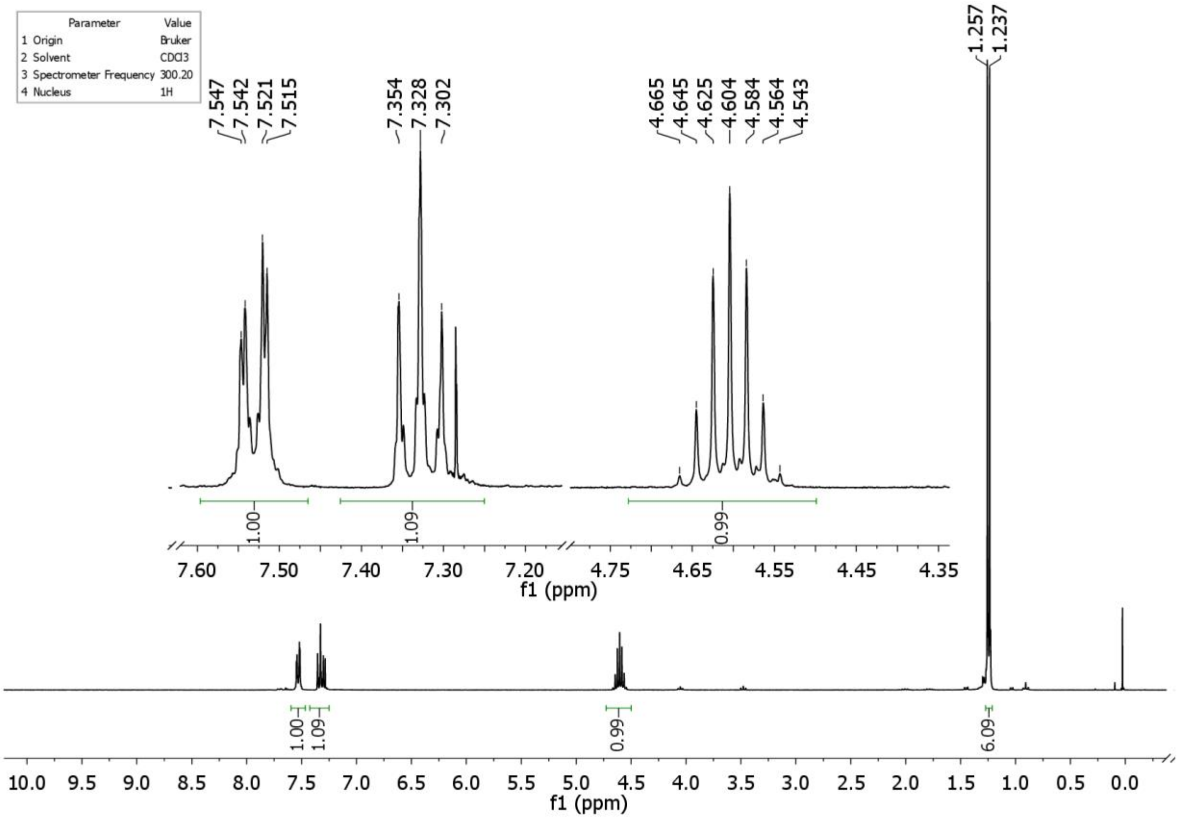

Figure S5. ^{1}H NMR spectrum of **2** in $CDCl_3$.

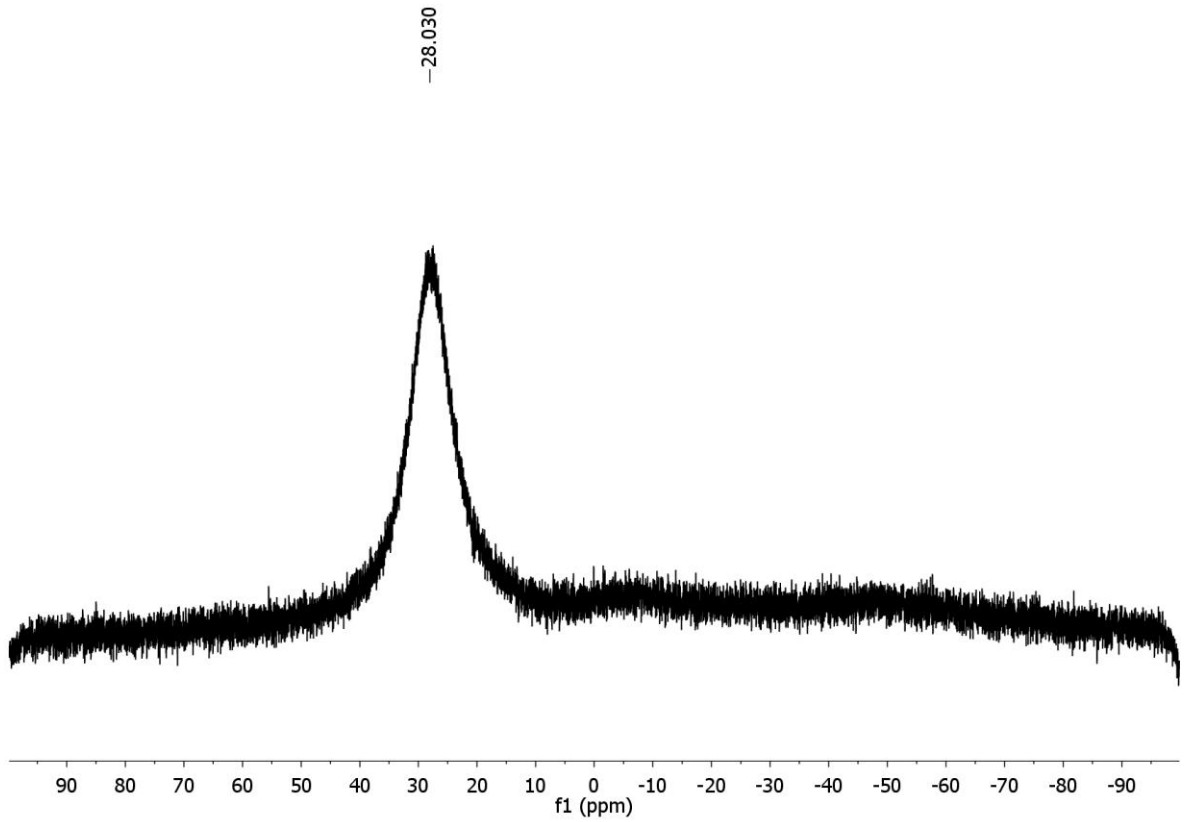

Figure S6. ^{11}B NMR spectrum of **2** in $CDCl_3$.

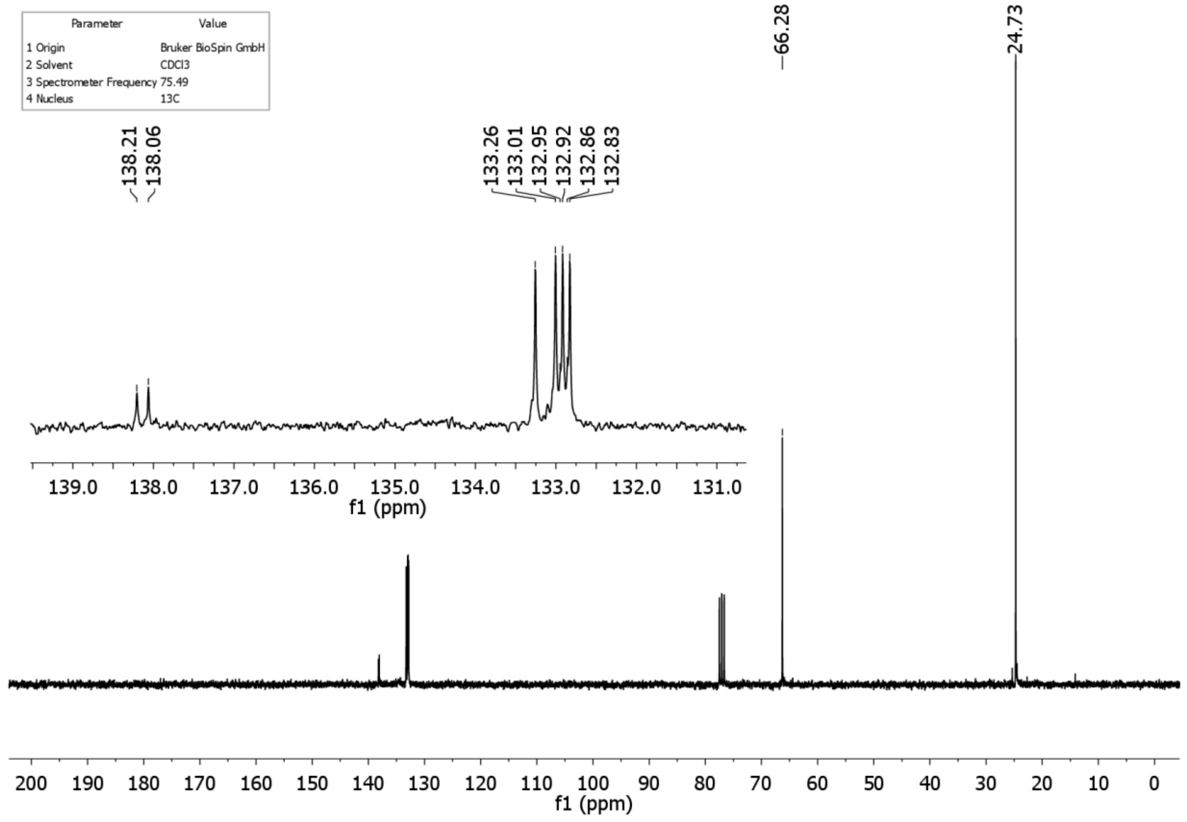

Figure S7. ^{13}C NMR spectrum of **2** in $CDCl_3$.

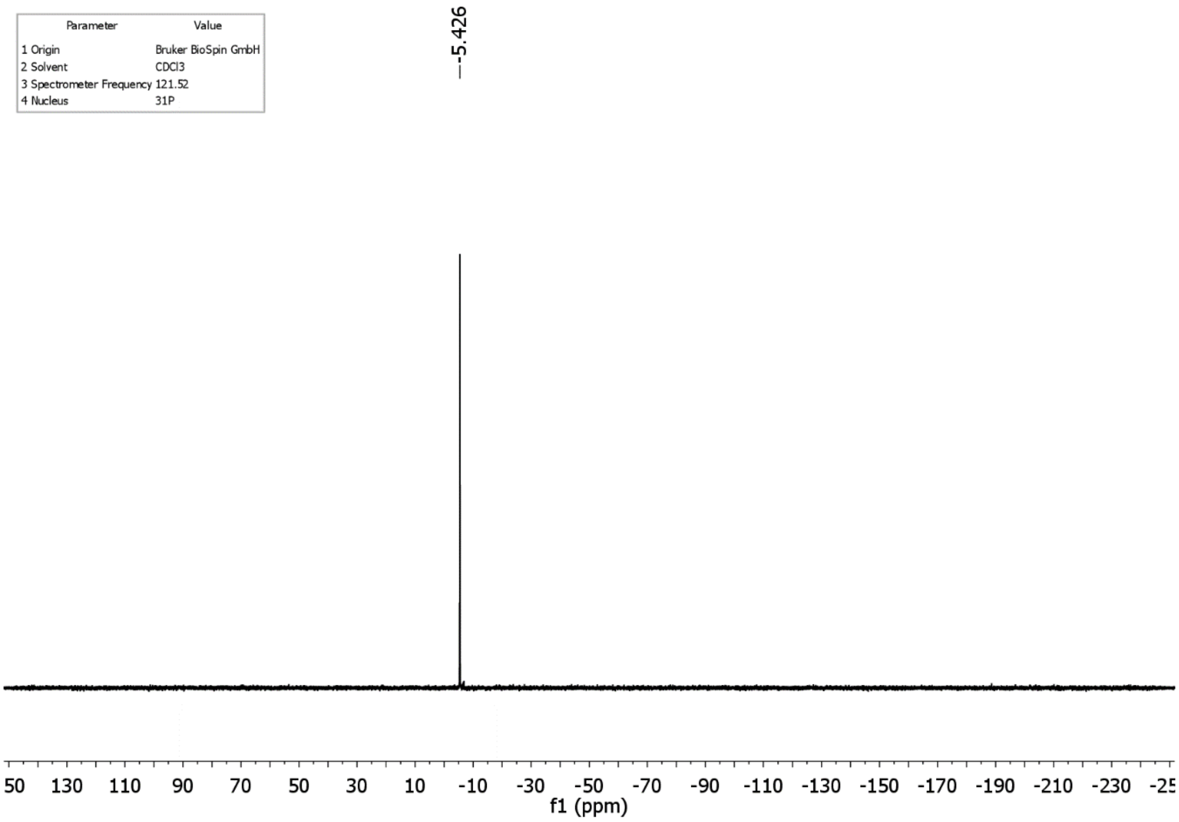

Figure S8. ^{31}P NMR spectrum of **2** in $CDCl_3$.

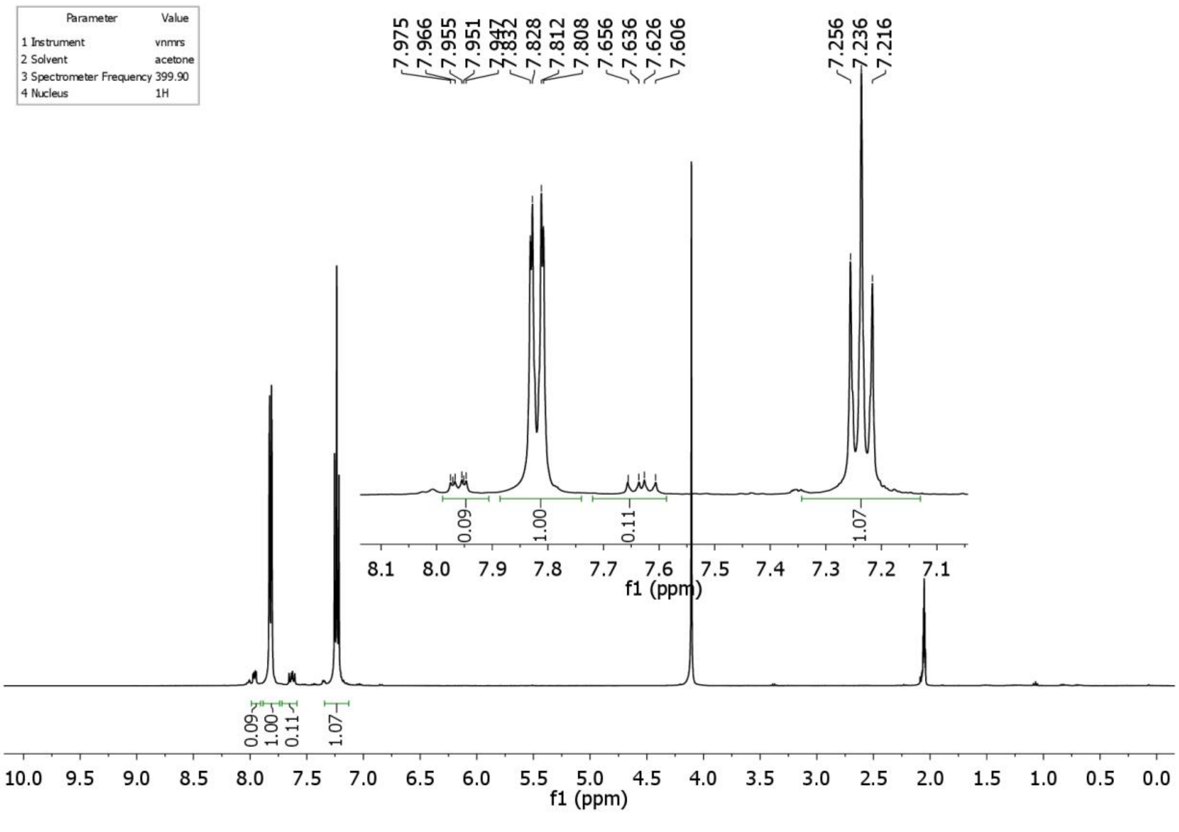

Figure S9. ^{1}H NMR spectrum of **2a** equilibrating with **2a'** (minor form) in acetone-d_6 + D_2O.

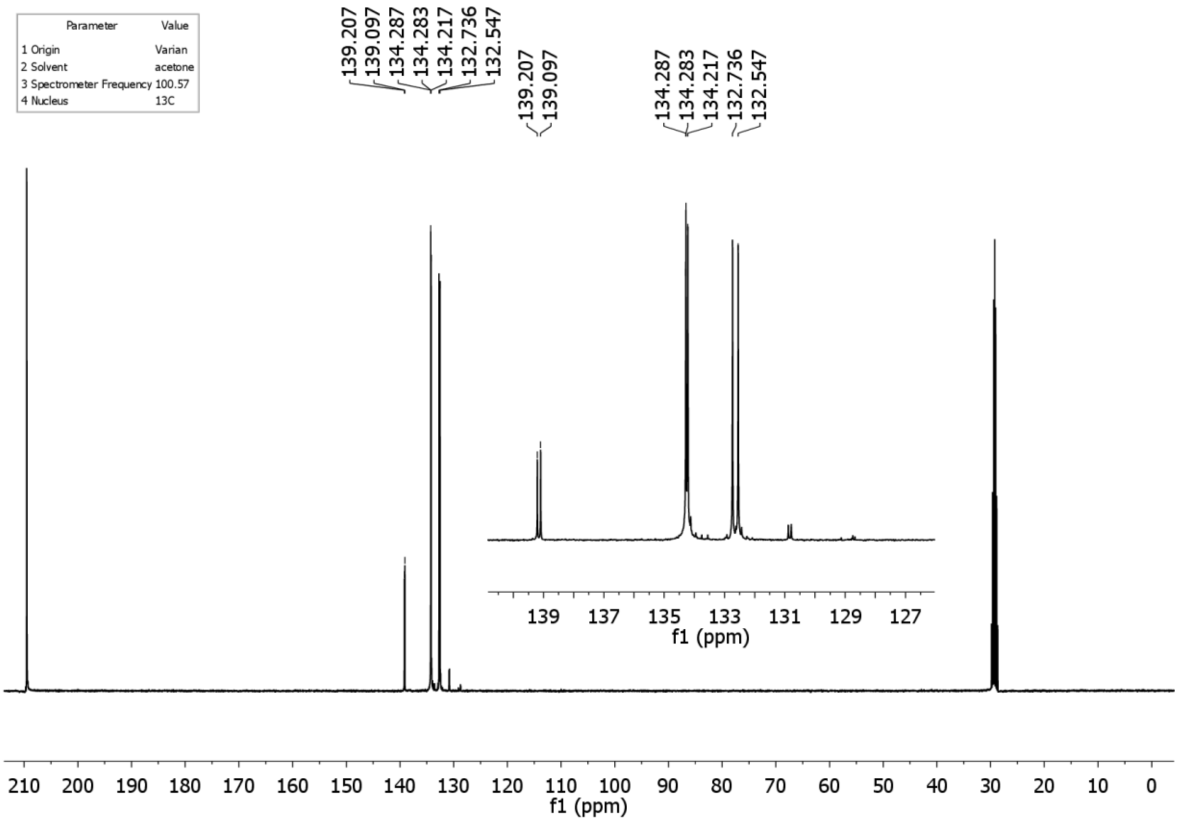

Figure S10. ^{13}C NMR spectrum of **2a** equilibrating with **2a’** (minor form) in acetone-d_6+ D_2O.

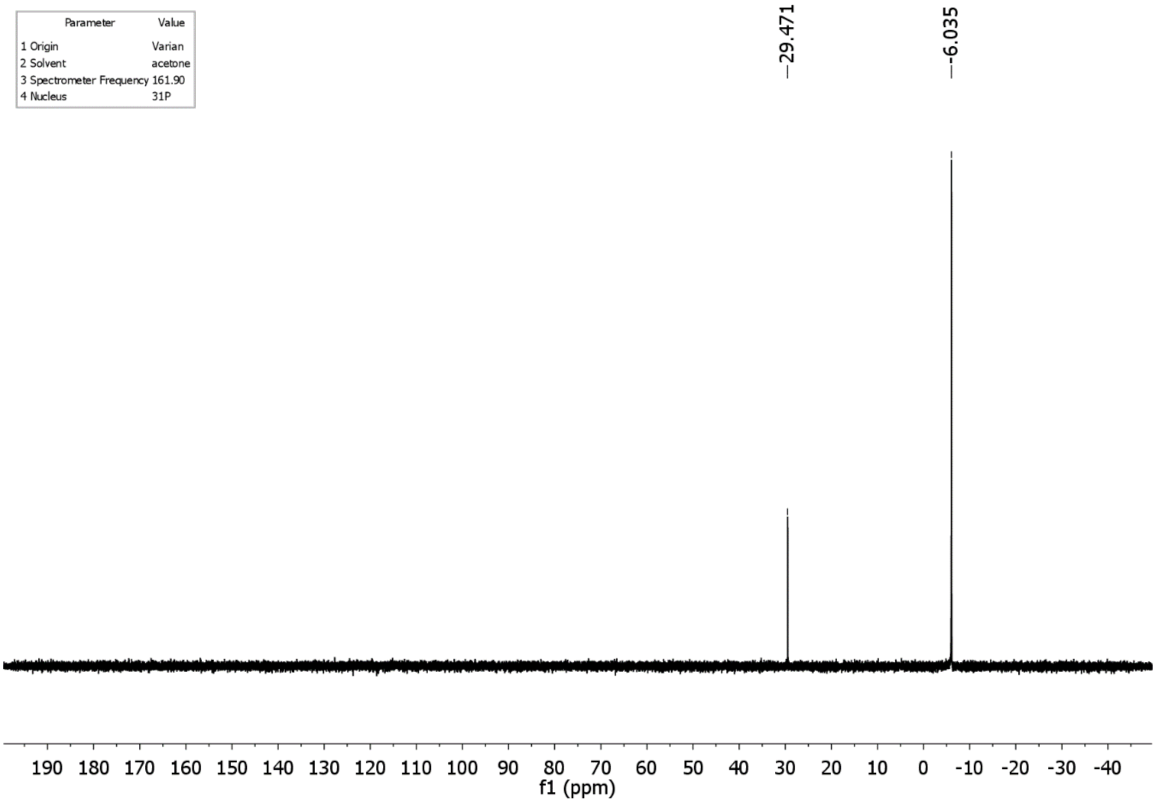

Figure S11. ^{31}P NMR spectrum of **2a** equilibrating with **2a’** (minor form) in acetone-d_6+ D_2O.

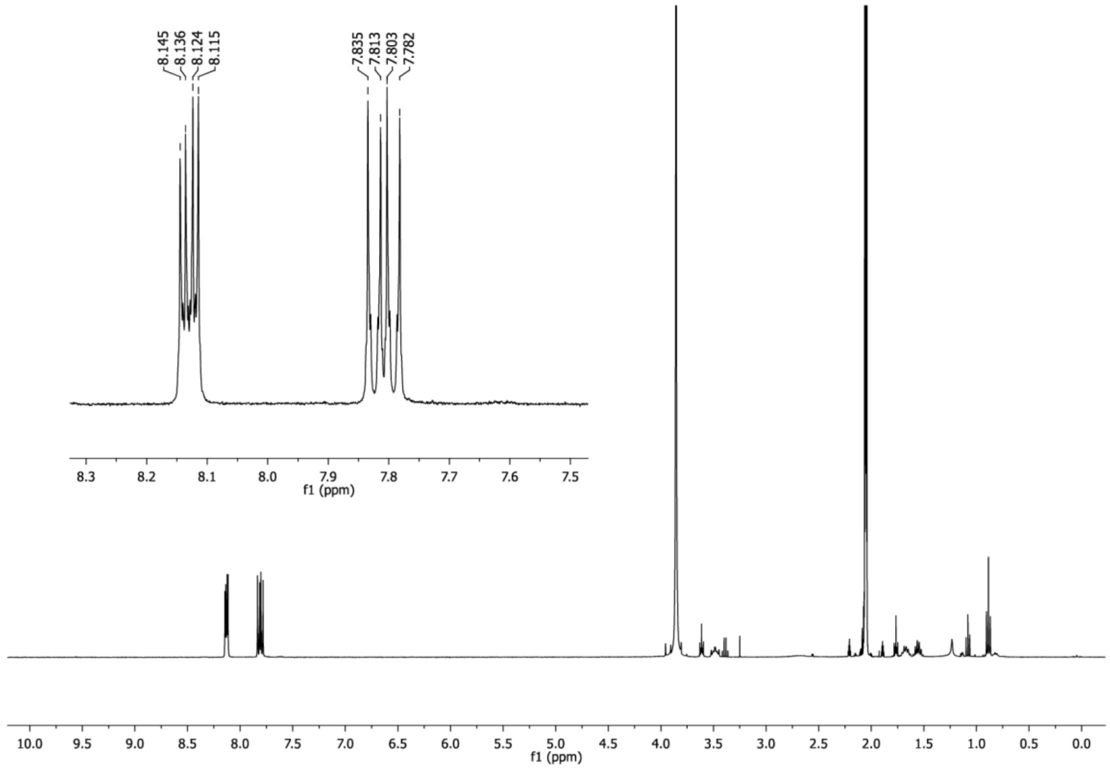

Figure S12. ^{31}P NMR spectrum of **2a’’** in acetone-d_6 + D_2O.

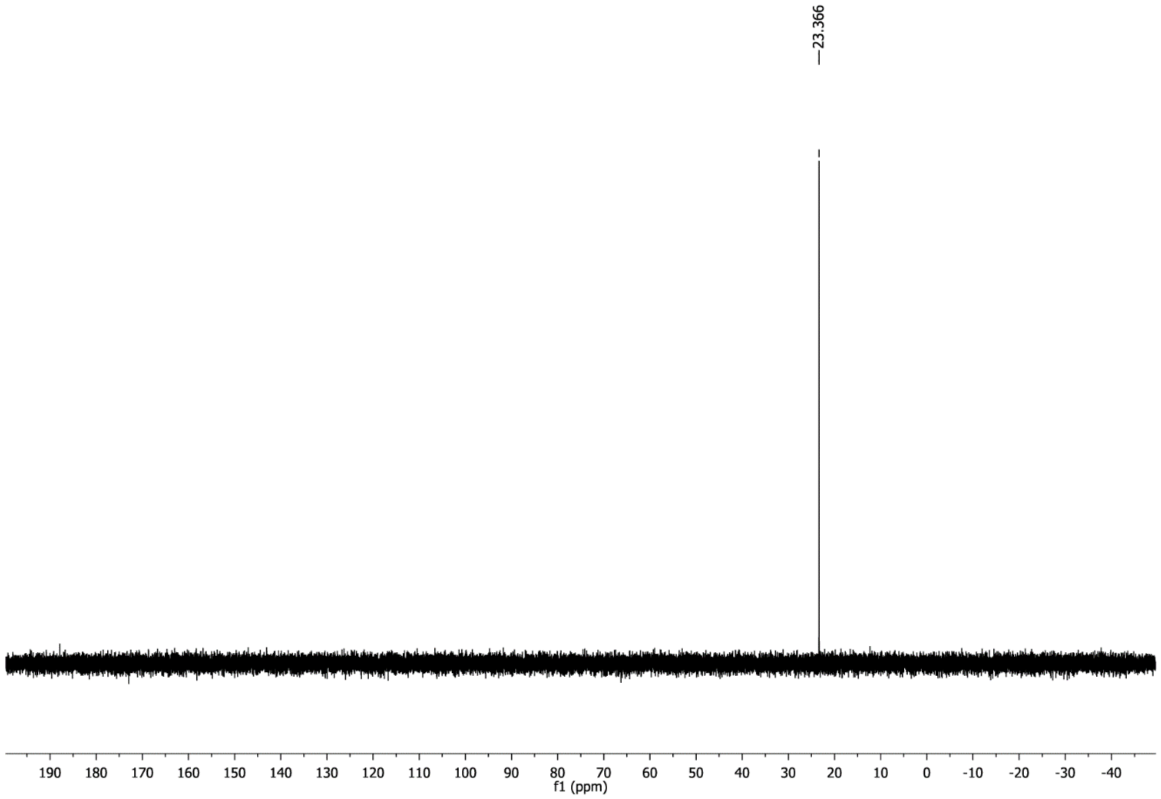

Figure S13. ^{31}P NMR spectrum of **2a’’** in acetone-d_6 + D_2O.

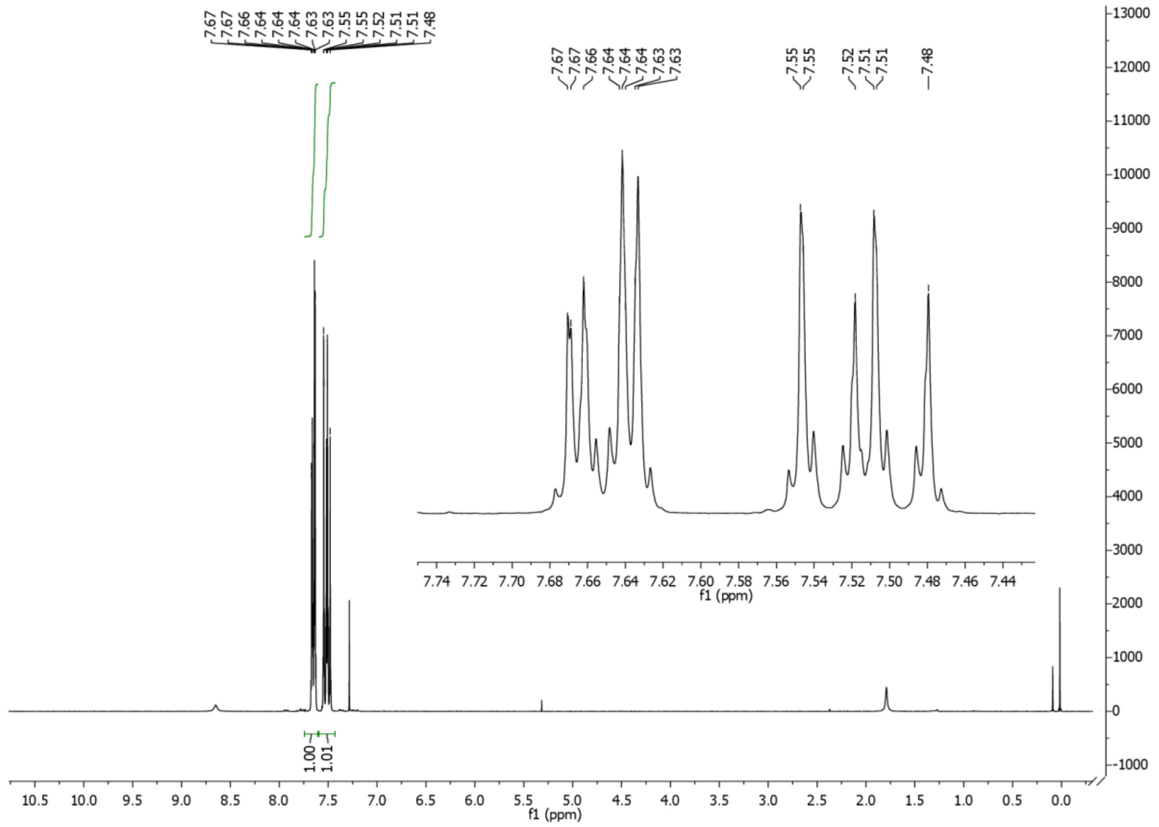

Figure S14. ^{1}H NMR spectrum of **3** in $CDCl_3$.

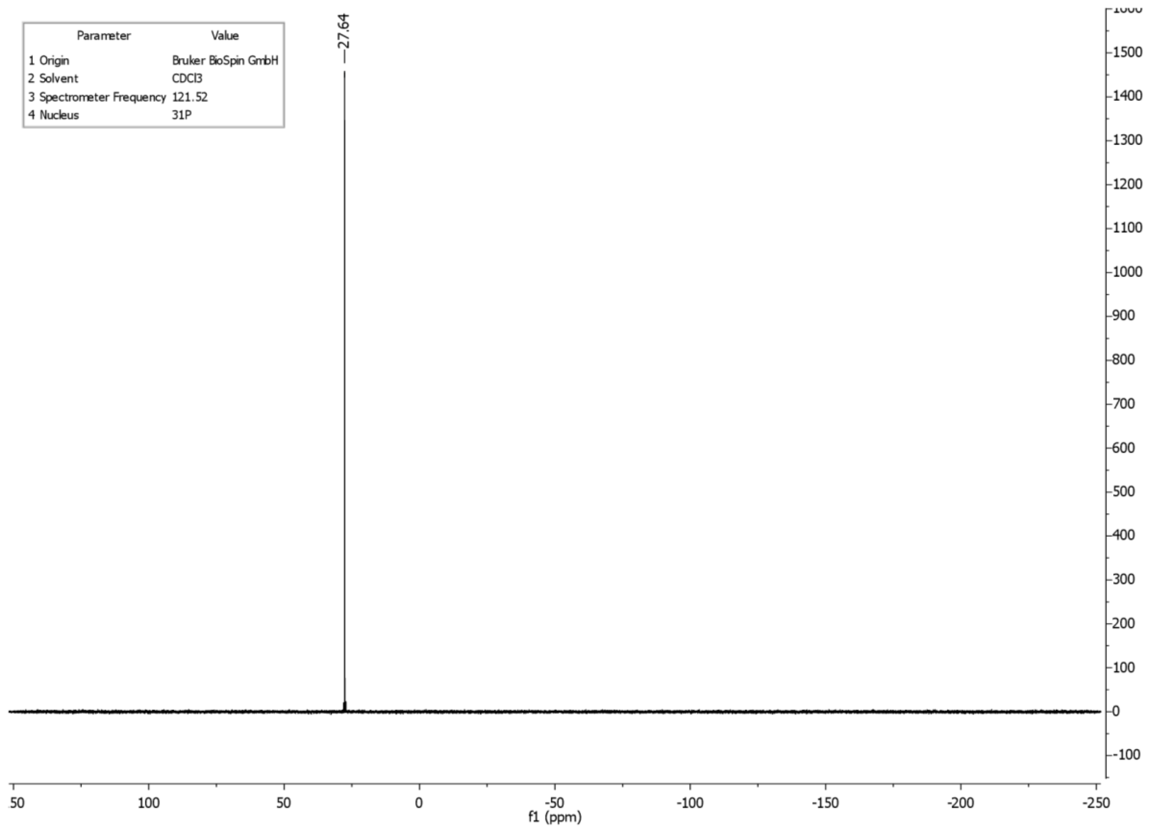

Figure S15. ^{31}P NMR spectrum of **3** in $CDCl_3$.

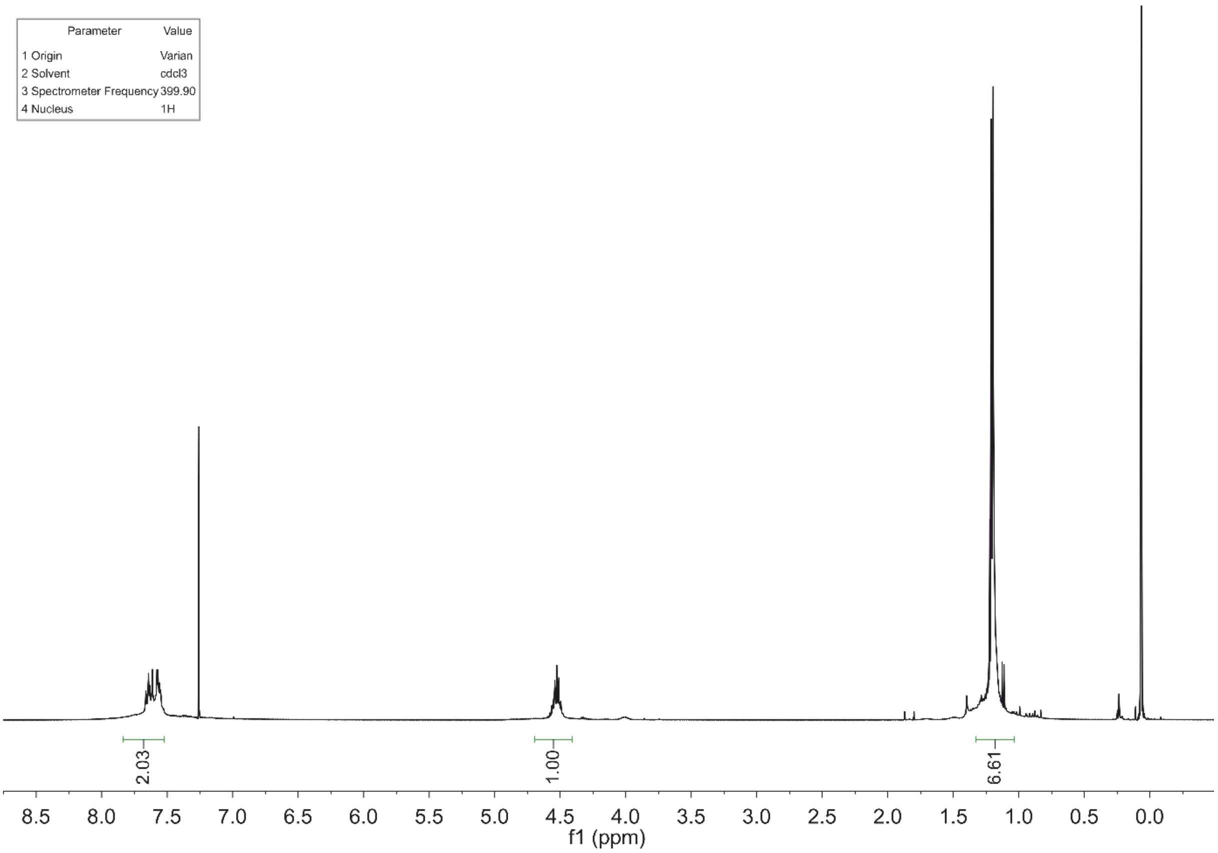

Figure S16. ^{1}H NMR spectrum of **4** in $CDCl_3$.

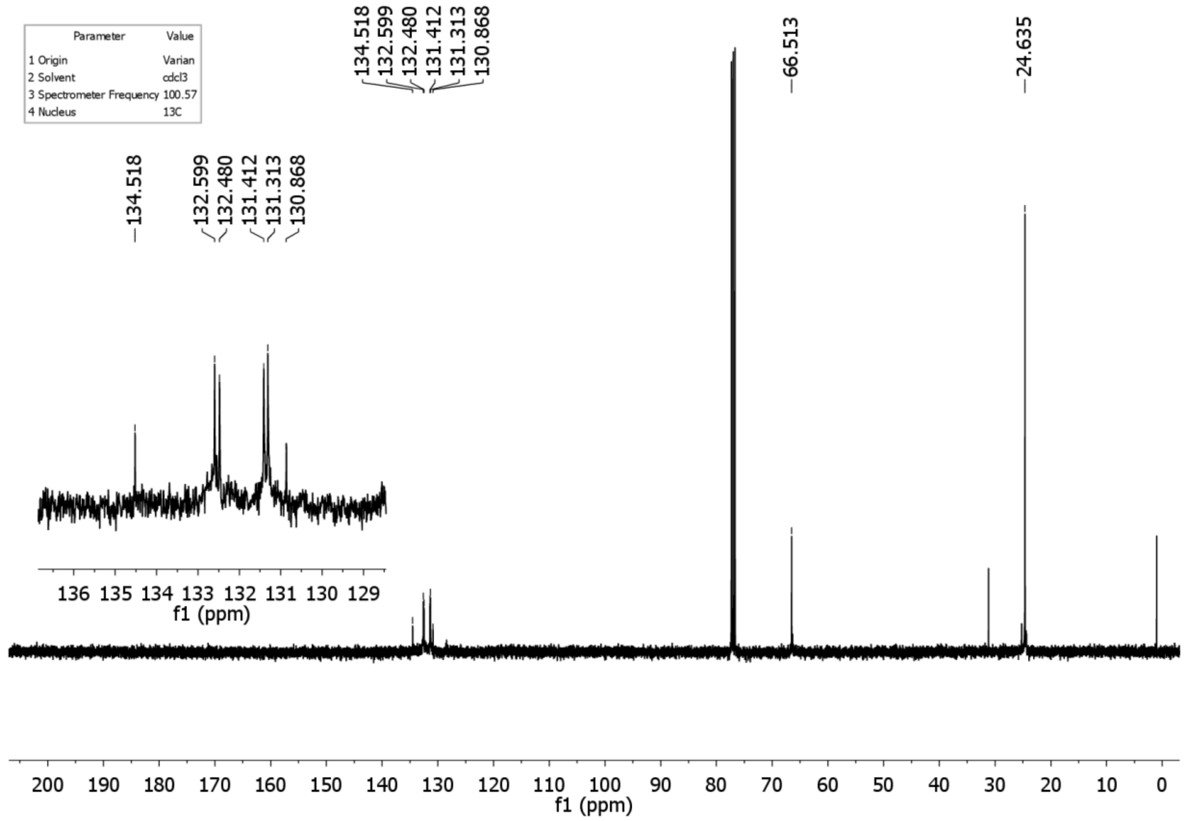

Figure S17. ^{13}C NMR spectrum of **4** in $CDCl_3$.

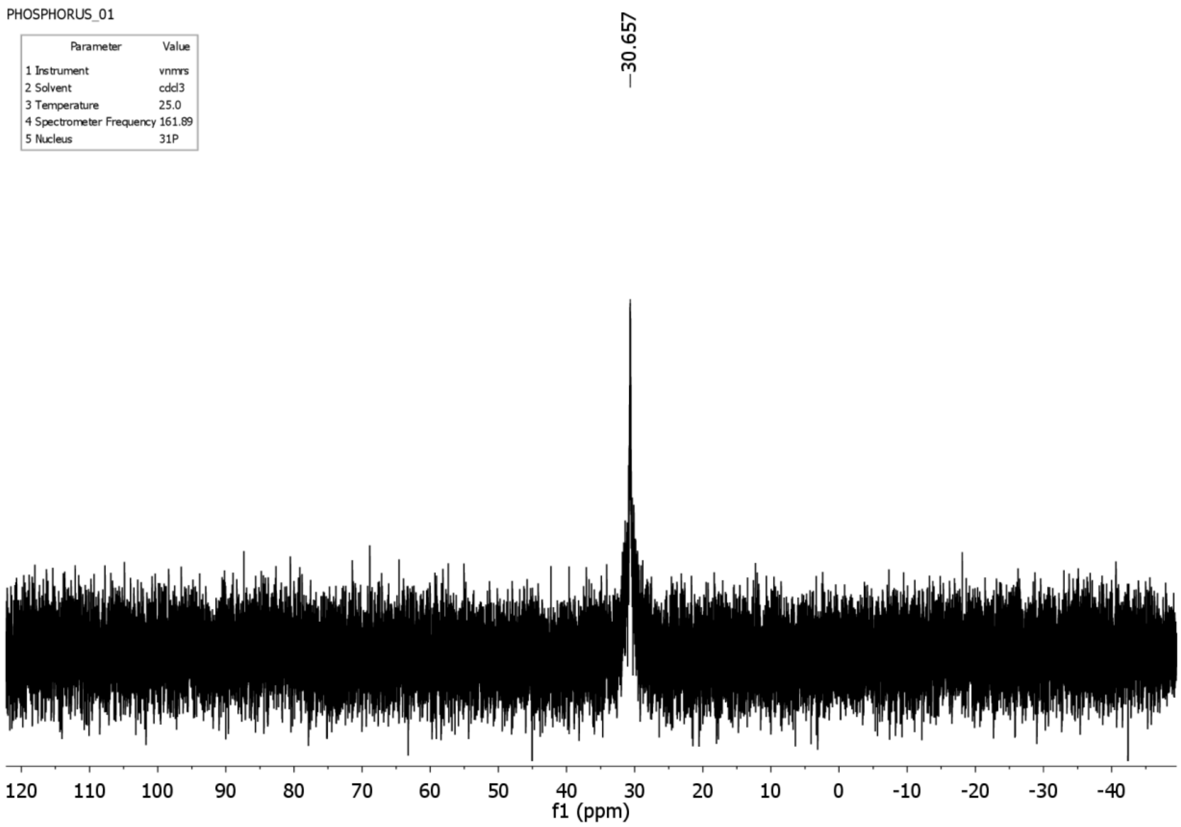

Figure S18. ^{31}P NMR spectrum of **4** in $CDCl_3$.

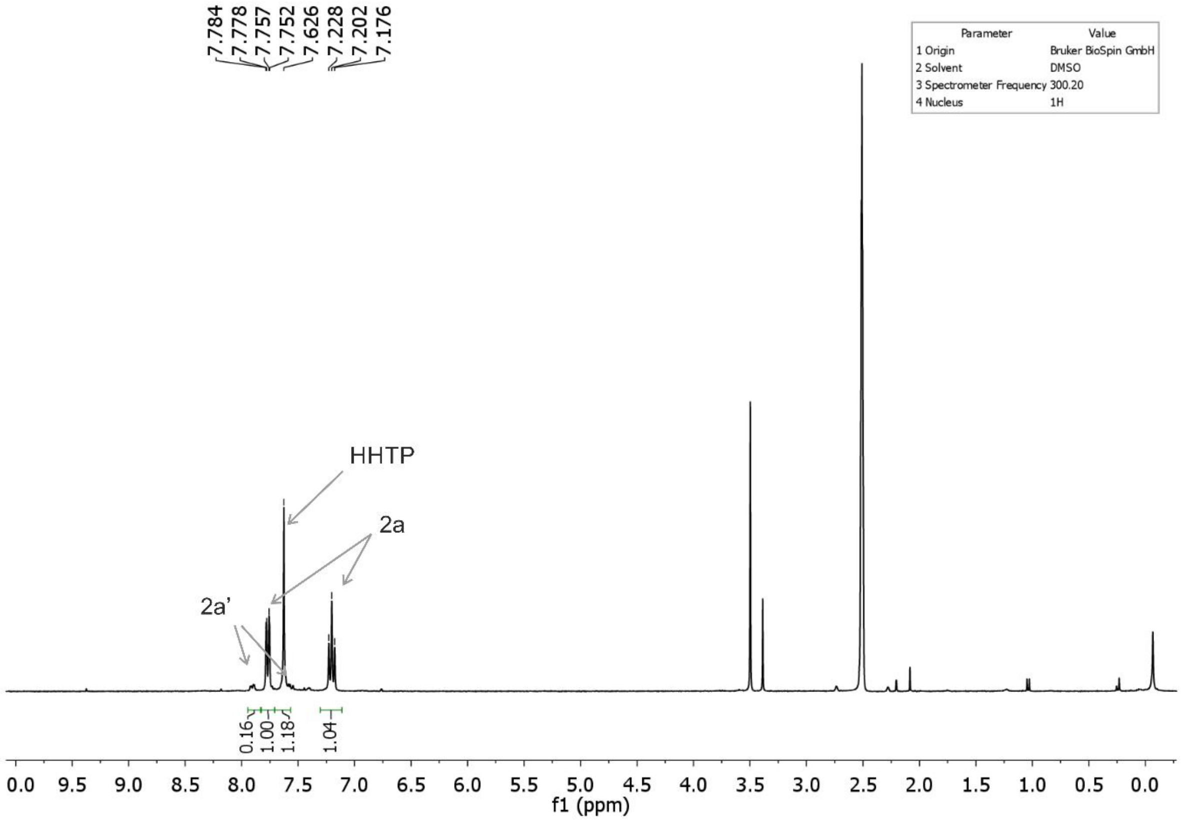

Figure S19. ^{1}H NMR spectrum of hydrolyzed (DMSO-d_6/D_2O) sample **BP1**.

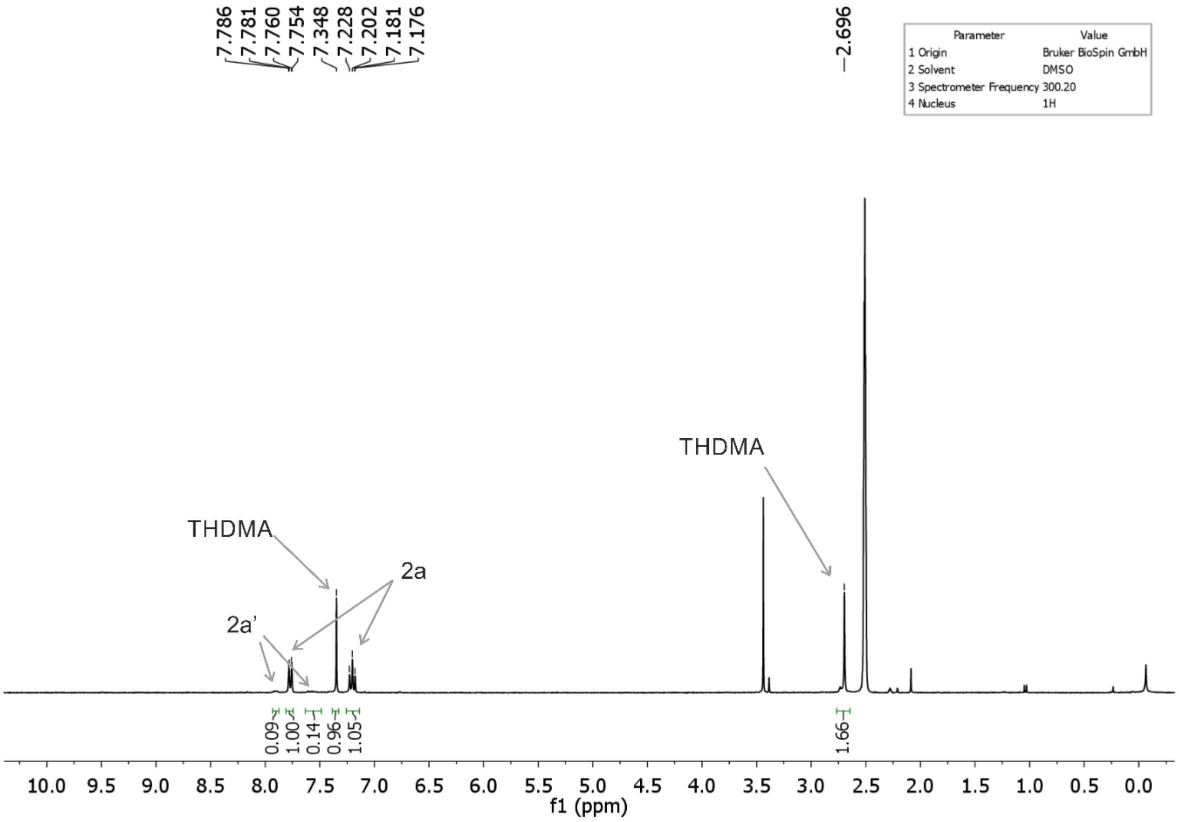

Figure S20. ^{1}H NMR spectrum of hydrolyzed (DMSO-d_6/D_2O) sample of **BP2**.

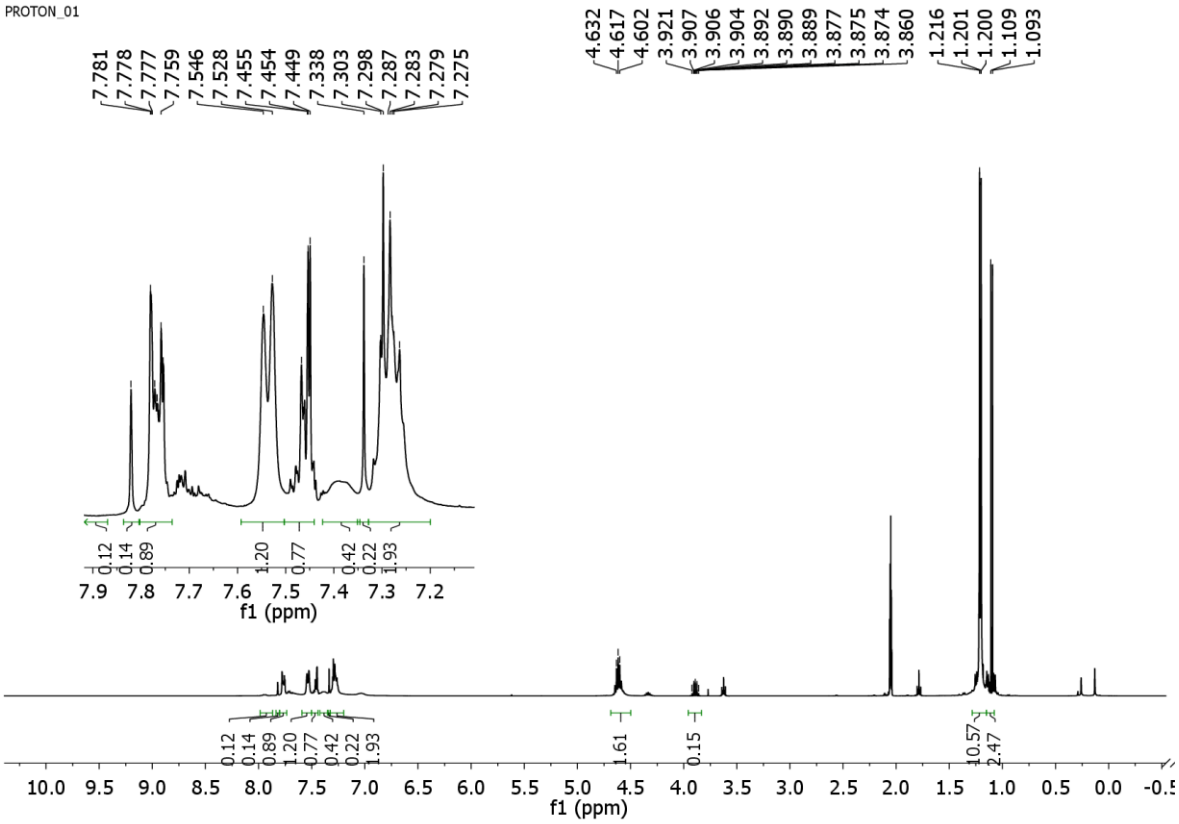

Figure S21. ^{1}H NMR spectrum of **[2]₄Pd** in $CDCl_3$. At least two different forms can be distinguished.

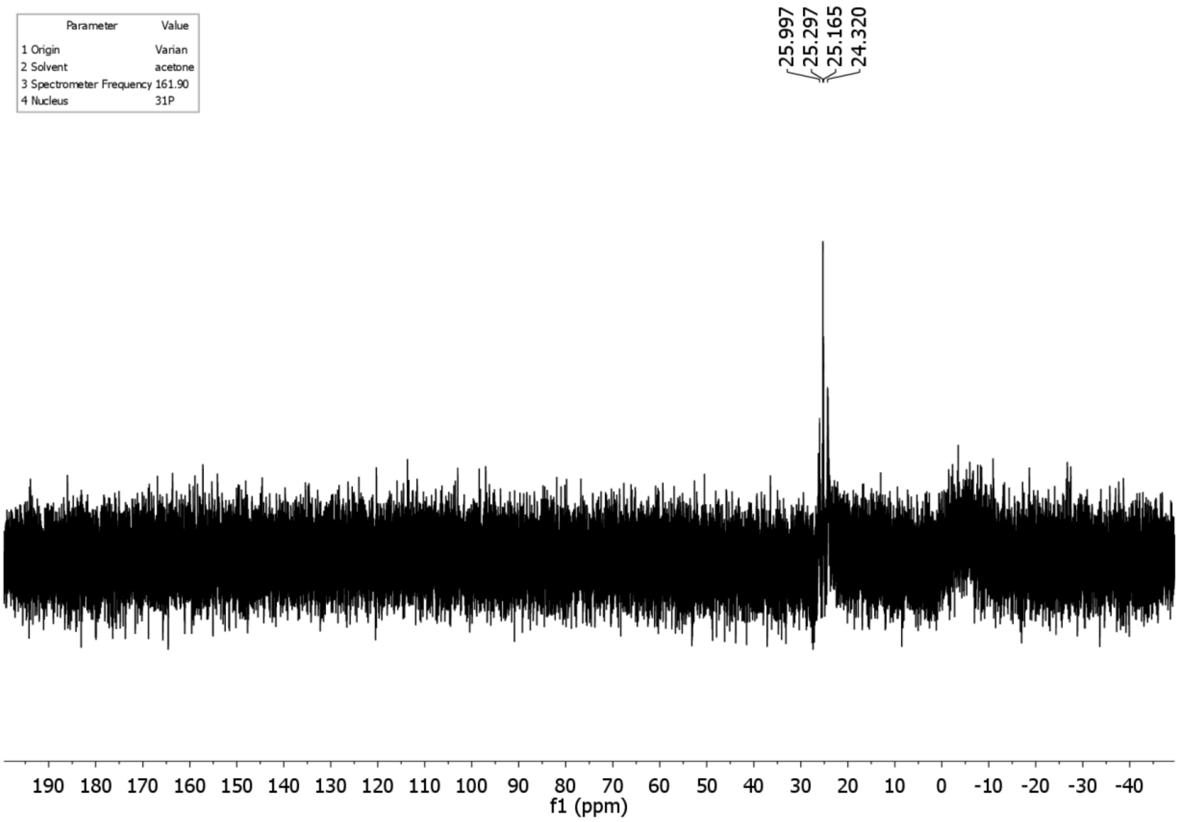

Figure S22. ^{31}P NMR spectrum of **[2]$_4$Pd** in $CDCl_3$.

4. MAS NMR spectra

Solid state ^{11}B and ^{31}P MAS NMR spectra were collected on a Bruker Avance II 500 MHz. Samples were packed in triple-resonance zirconia probes (Bruker MAS DVT 4 mm) and spun at 10 kHz during data collection. ^{1}H decoupling was applied during data acquisition. A recycle delay of 5 and 10 s was used for ^{11}B and ^{31}P MAS NMR experiments, respectively. The ^{11}B MAS NMR chemical shifts are given relative to BF_3 etherate and calibrated using solid $NaBF_4$ assigned to –1.7 ppm as a secondary reference. The ^{31}P MAS NMR chemical shifts are given relative to 85wt% aq. H_3PO_4 and calibrated using solid PPh_3 assigned to –6.0 ppm as a secondary reference. The recorded ^{11}B MAS NMR spectra were dominated by very intense broad background signal of asymmetric shape with maximum at ca. 8 ppm arising from boron nitride used for construction of the MAS NMR stator. Therefore, the final spectra were obtained in a differential mode by substracting the spectrum of the reference probe (filled with PPh_3) from the original spectrum containing the analyzed material.

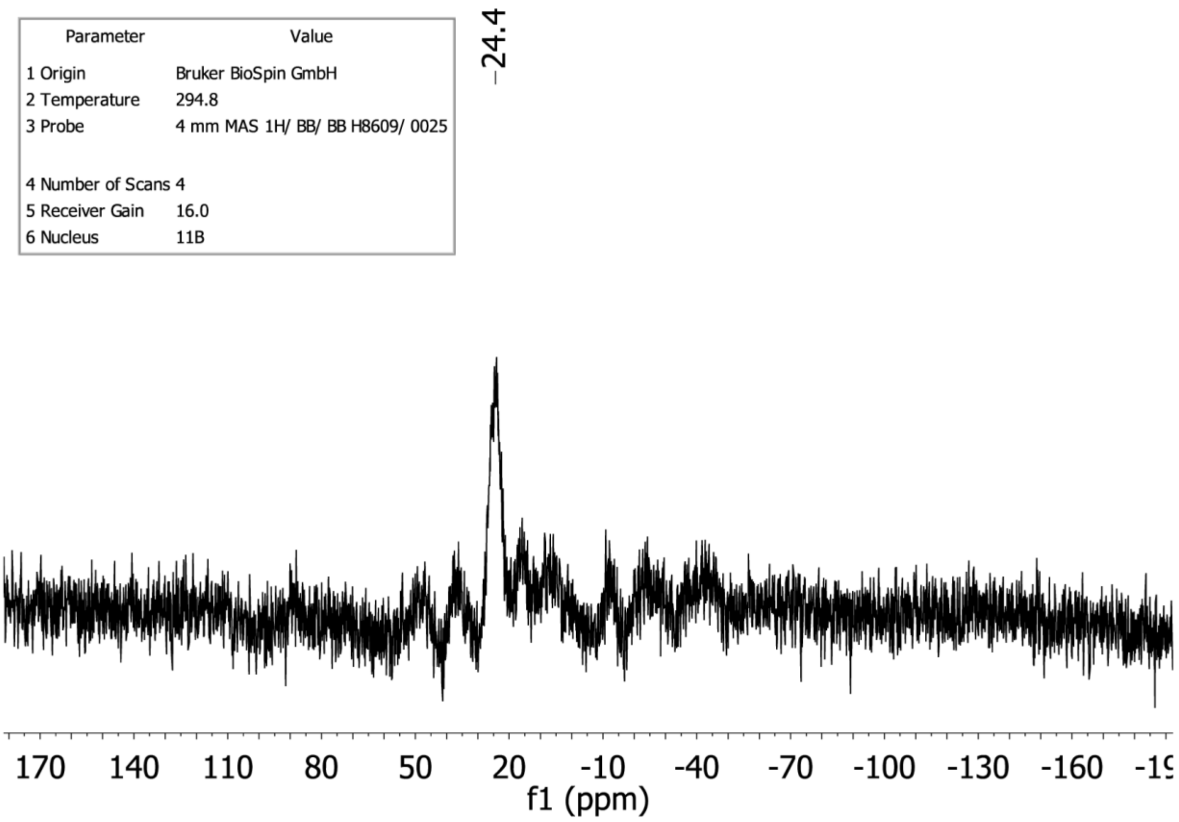

Figure S23. ^{11}B MAS NMR spectrum of **BP2.**

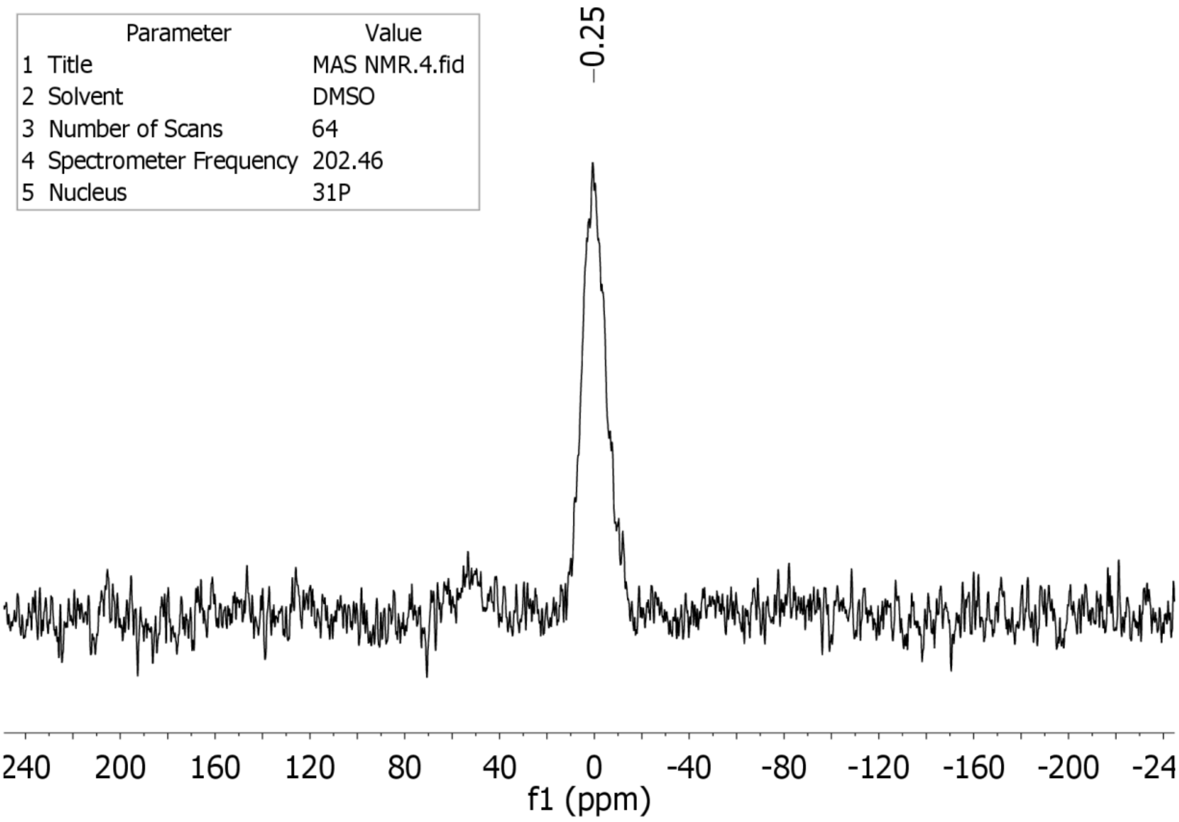

Figure S24. ^{31}P MAS NMR spectrum of **BP2.**

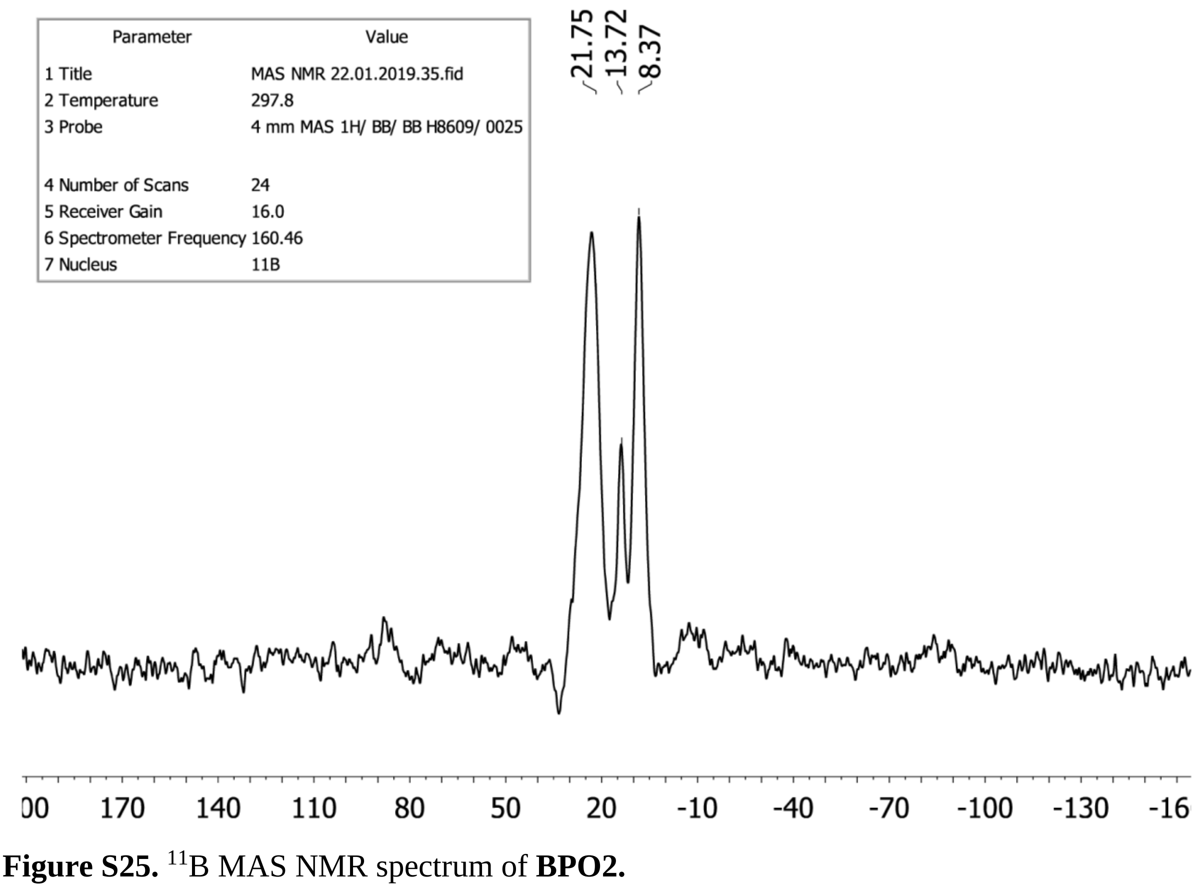

Figure S25. ^{11}B MAS NMR spectrum of **BPO2.**

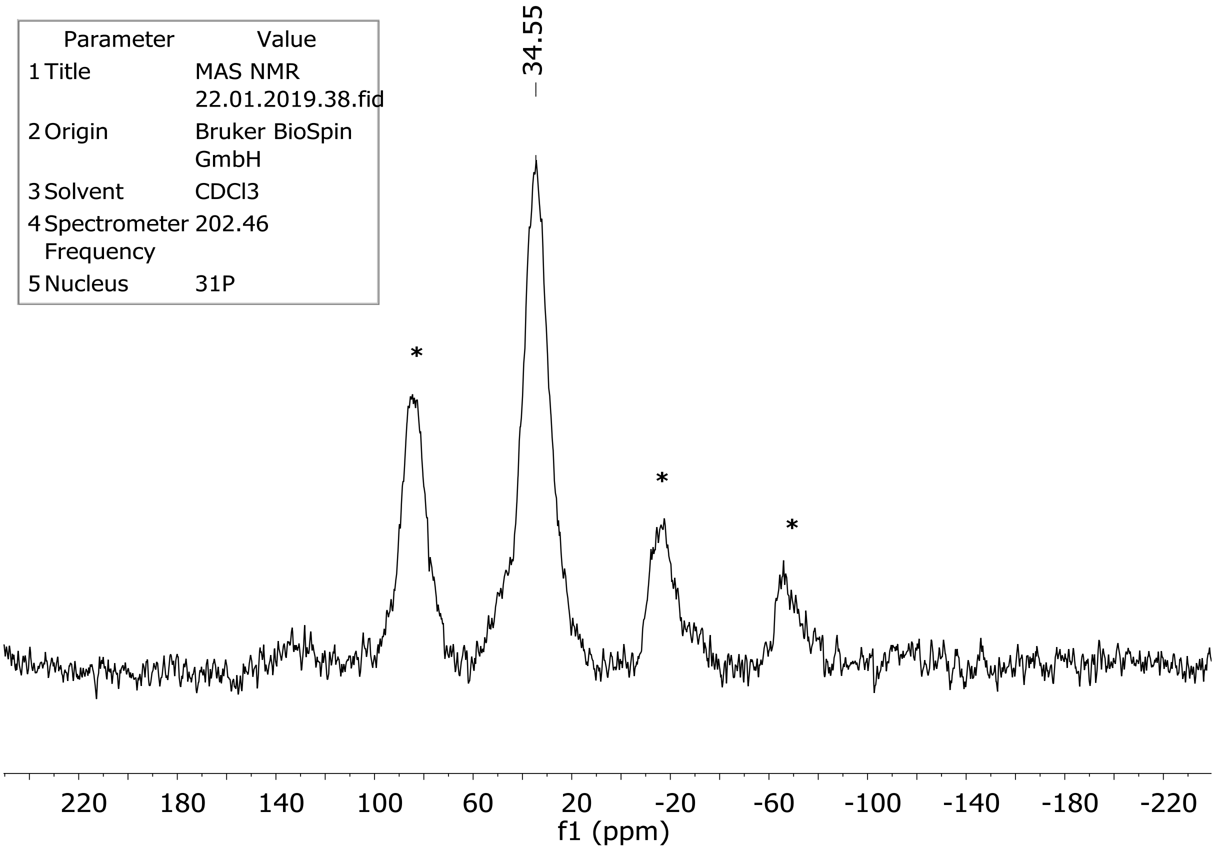

Figure S26. ^{31}P MAS NMR spectrum of **BPO2**. Asterisks (*) indicate peaks arising from spinning side bands.

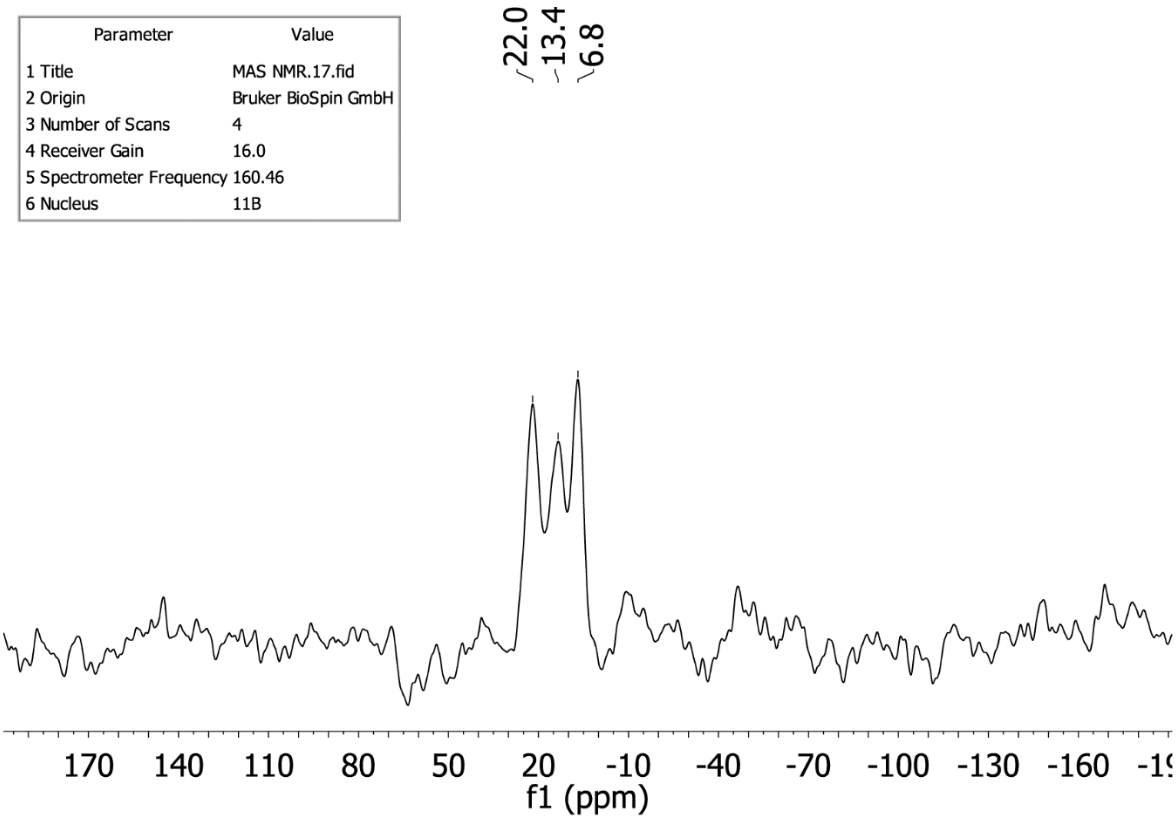

Figure S27. ^{11}B MAS NMR spectrum of **BP2-Pd(BU).**

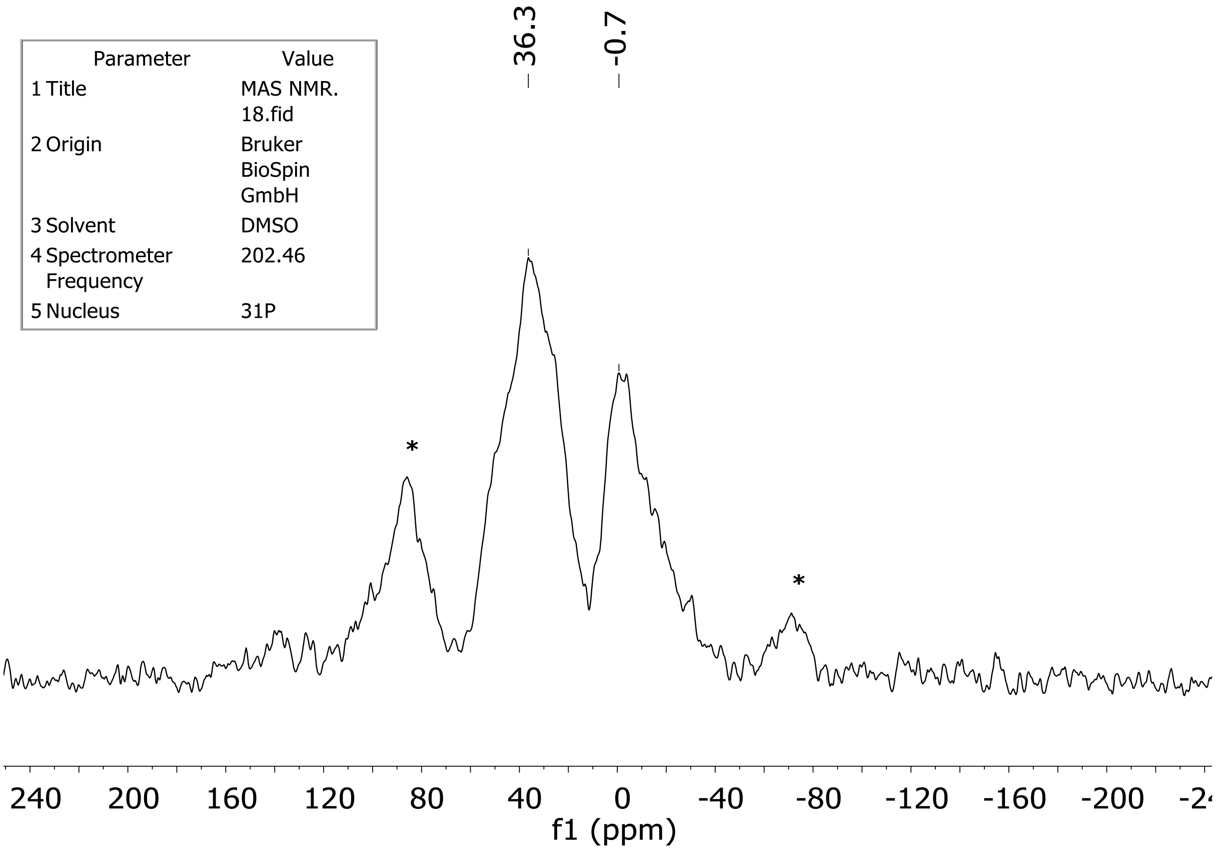

Figure S28. ^{31}P MAS NMR spectrum of **BP2-Pd(BU).** Asterisks (*) indicate peaks arising from spinning side bands.

5. XPS spectra

X-ray photoelectron spectroscopy (XPS) measurements were recorded by a PHI 500 VersaProbe (ULVAC − PHI) spectrometer with monochromatic Al-*Kα* radiation (h*ν* = 1486.6 eV).

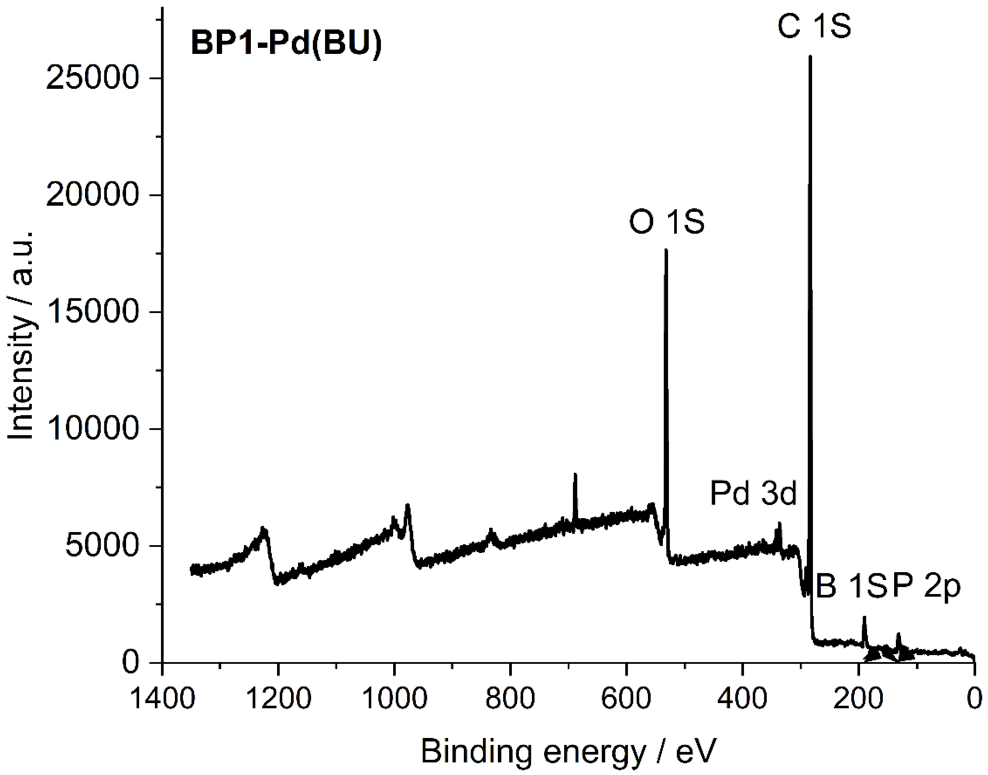

Figure S29. Wide-scan of survey XPS spectrum of **BP1-Pd(BU)**.

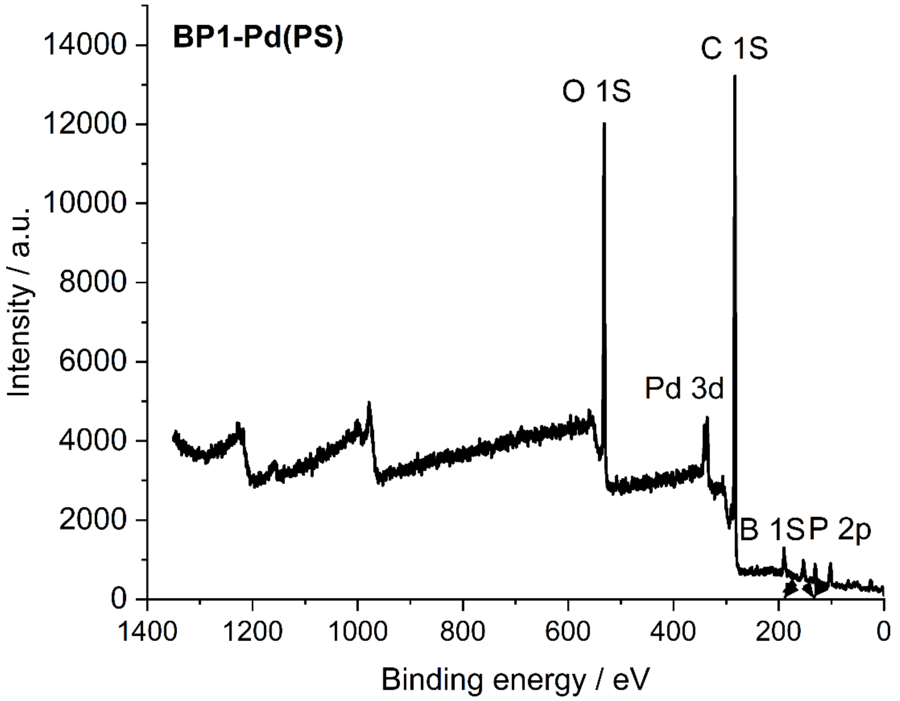

Figure S30. Wide-scan of survey XPS spectrum of **BP1-Pd(PS)**.

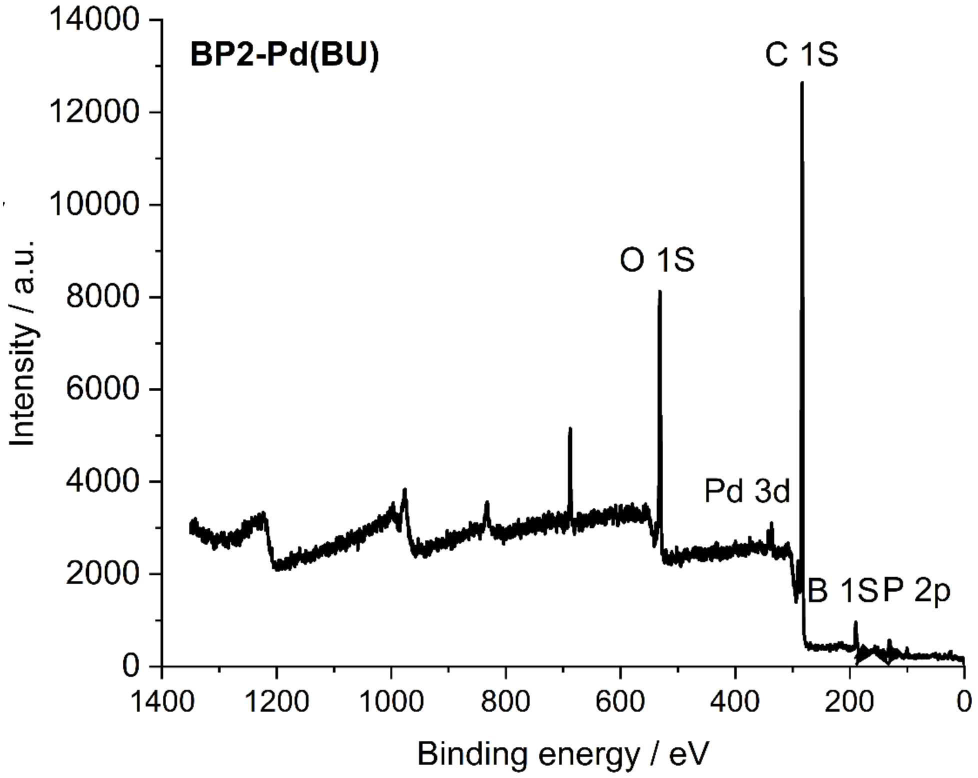

Figure S31. Wide-scan of survey XPS spectrum of **BP2-Pd(BU)**.

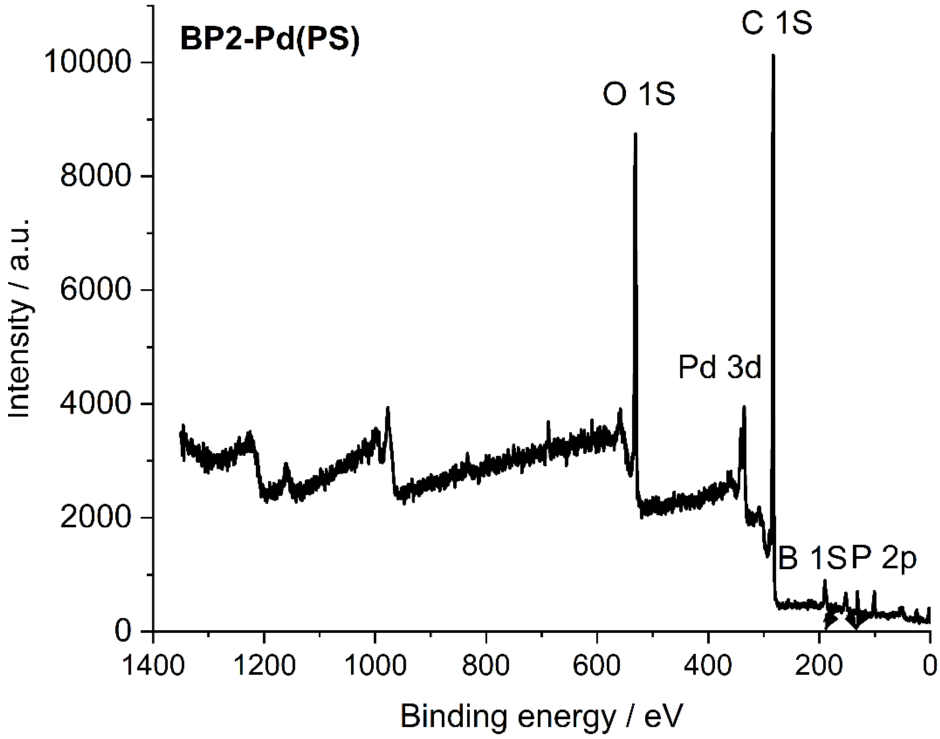

Figure S32. Wide-scan of survey XPS spectrum of **BP2-Pd(PS)**.

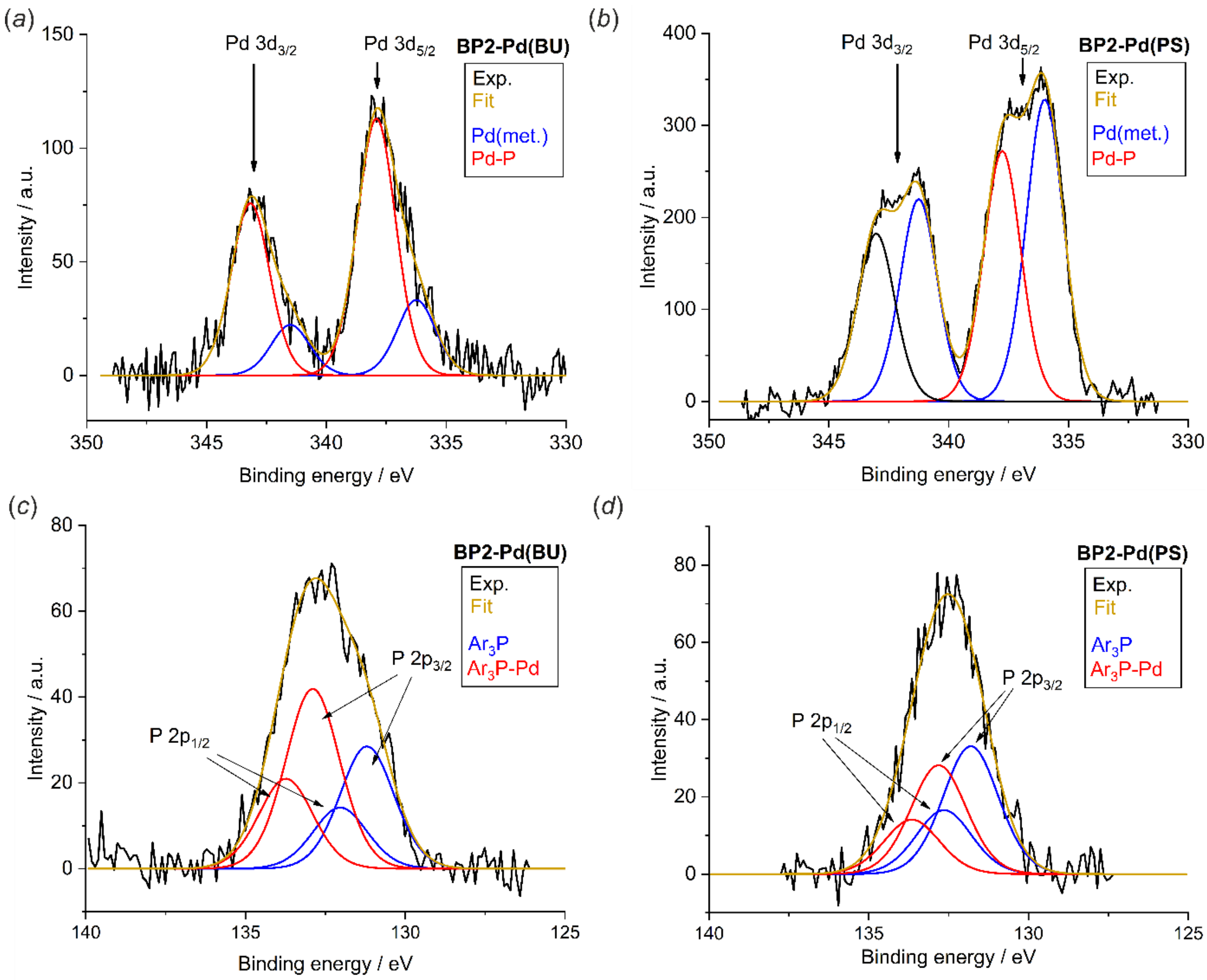

Figure S33. (*a*, *b*) Pd 3d and (*c*, *d*) P 2p high-resolution XPS spectra investigation of (*a*, *c*) **BP2-Pd(BU)** and (*b*, *d*) **BP2-Pd(PS)**.

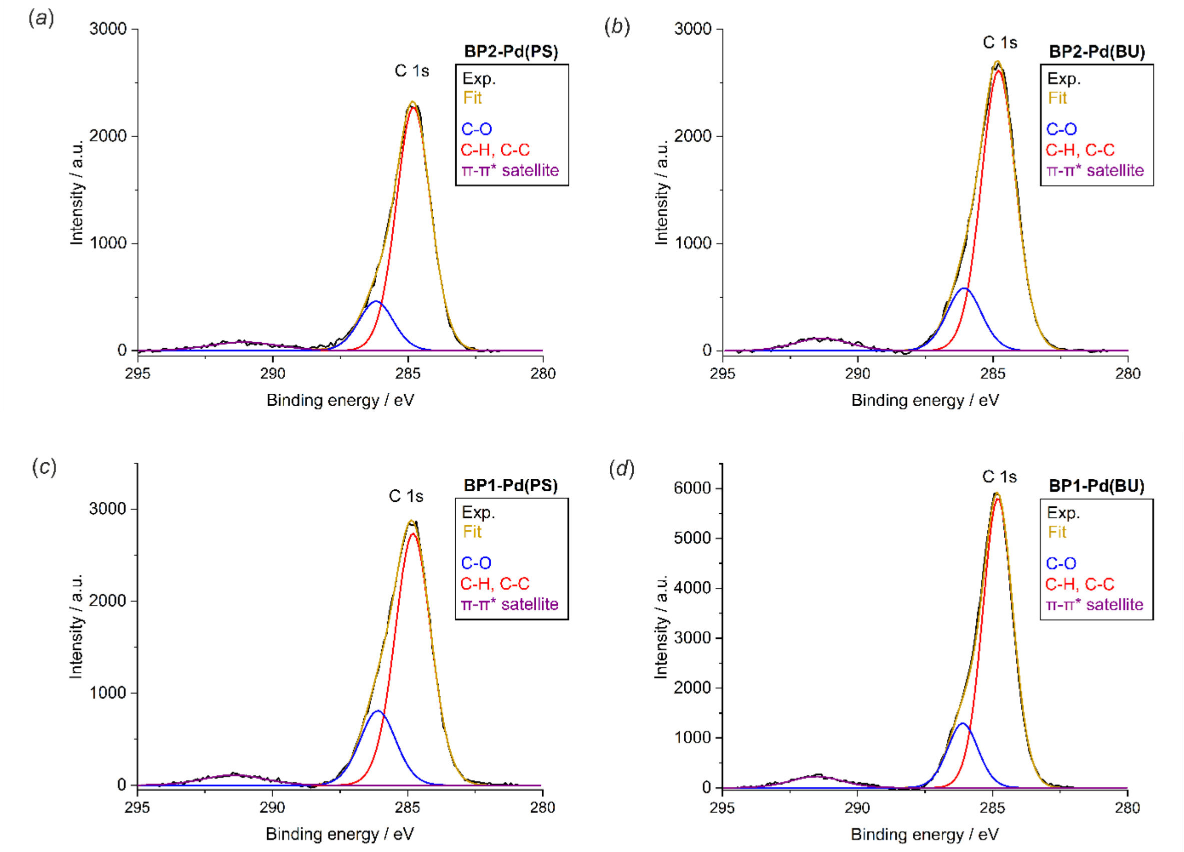

Figure S34. C 1s high-resolution XPS spectra investigation Pd-functionalized COFs.

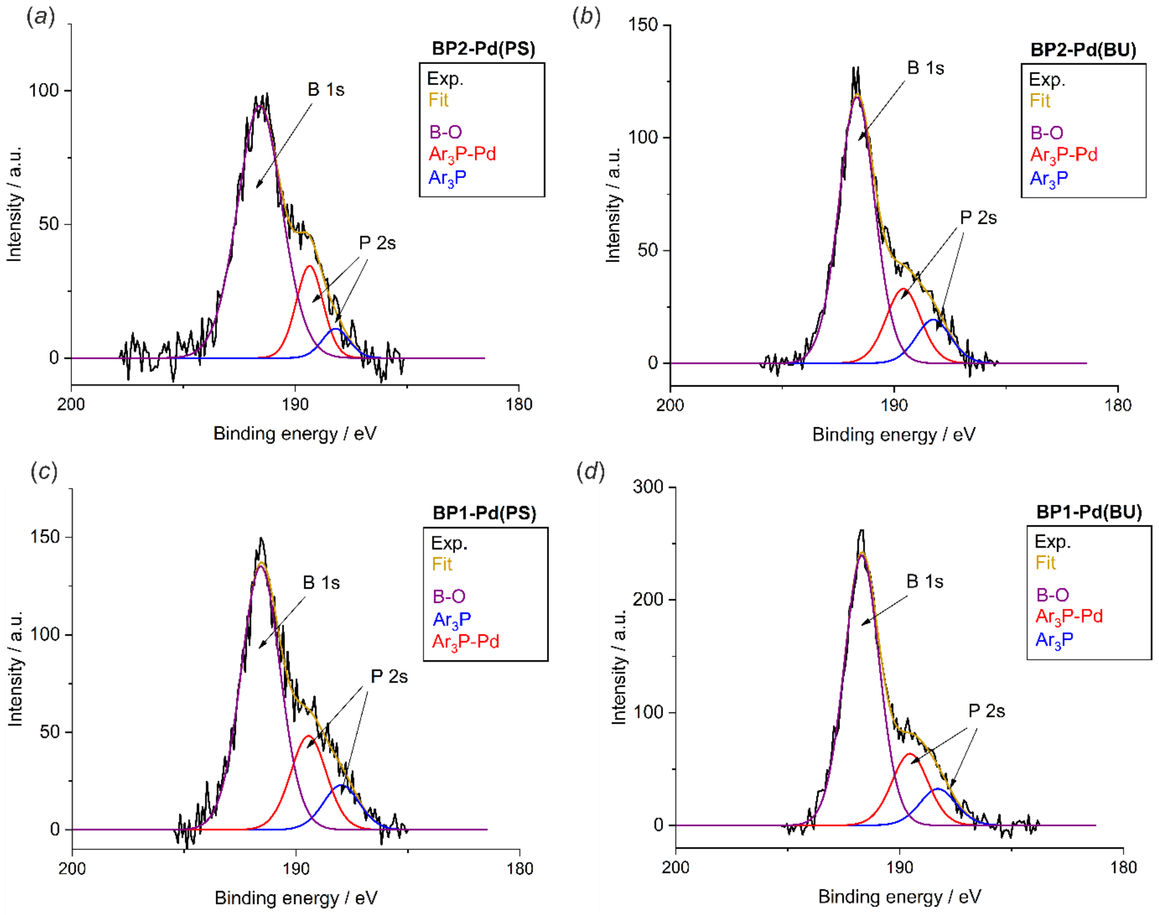

Figure S35. B 1s high-resolution XPS spectra investigation Pd-functionalized COFs.

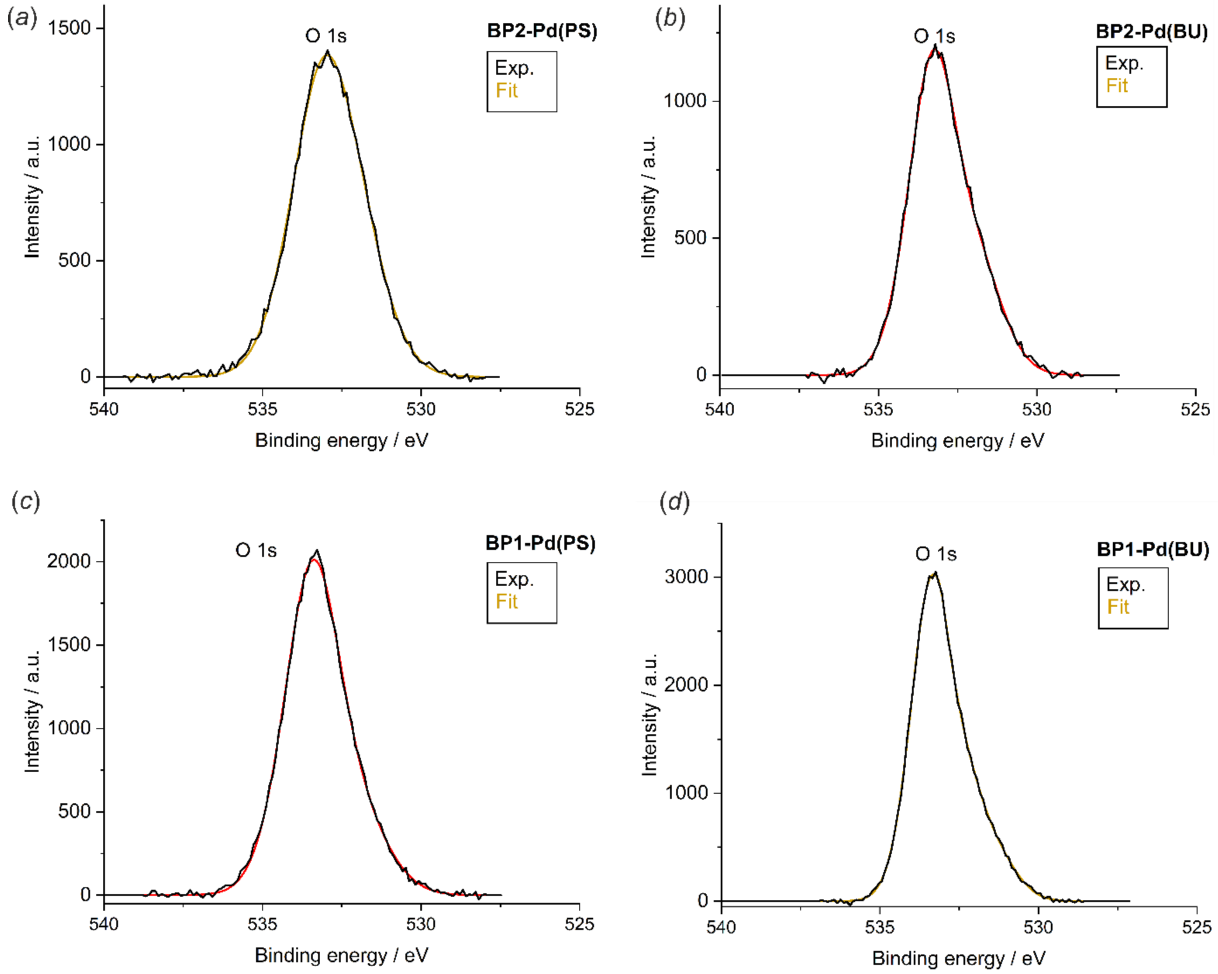

Figure S36. O 1s high-resolution XPS spectra investigation Pd-functionalized COFs.

6. PXRD

PXRD measurements were performed at ID11 Materials science beamline at the European Synchrotron Radiation Facility (ESRF), Grenoble, France. Energy selection (E = 69.6 keV, λ = 0.1781 Å) was achieved by using a double bent Si(111) monochromator. Data were collected using FReLoN 4m detector (50 μm x 50 μm pixel size) up to Q = 20 $Å^{-1}$ (Q = $4\pi\sin(\theta)/\lambda$). The sample was placed in 1.5 mm diameter Kapton capillary (sample to detector distance 20 cm) and exposed to X-ray beam (total exposure time 10min). Calibration of the detector was done using CeO_2 NIST SRM 674b material, and azimuthal data integration was performed in pyFAI.[6]

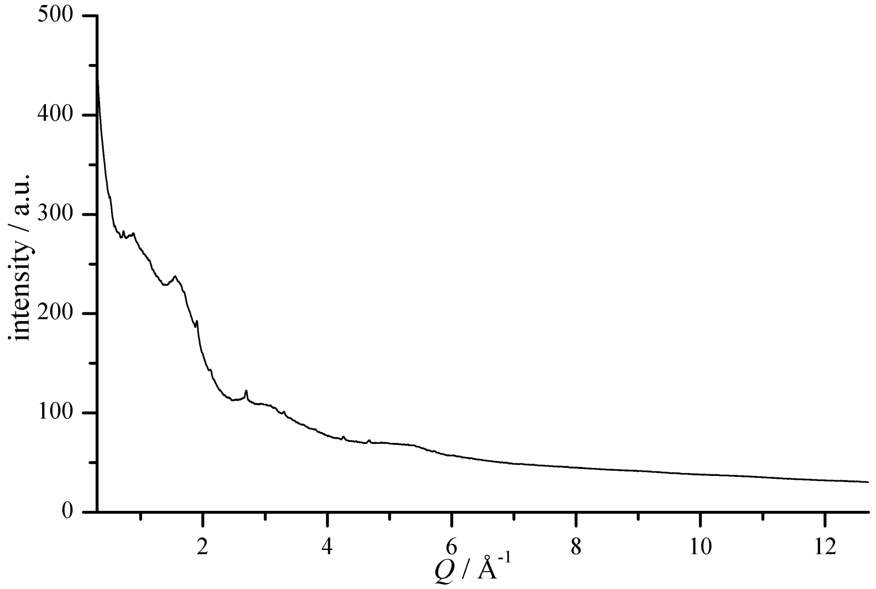

Figure S37. PXRD pattern of **BP2** obtained from synchrotron measurement.

7. SEM

The samples surface were observed using SEM/STEM Hitachi s 5500 with maximal resolution of 0.4 nm at acceleration voltage of 30 keV and below 2 nm at 1 keV. The observation was performed using SE (secondary electron) signal. The microscope was equipped with Cold Field Emission gun. This type of electron source in the microscope ensures high resolution at relatively low beam current, which is important in case of observation of sensitive materials like COFs. To minimize the effect of surface charging as well as to confine the structure damage by electrons, low beam current and low acceleration voltage (3 keV) were applied. Such observation conditions ensured enough brightness and resolution but were safe for the COFs samples. Additionally, low accelerating voltage allows to observe surface morphology in detail, since the electrons penetrate lower thickness of the sample. The samples were mounted on aluminum stage with the use of carbon adhesive tape.

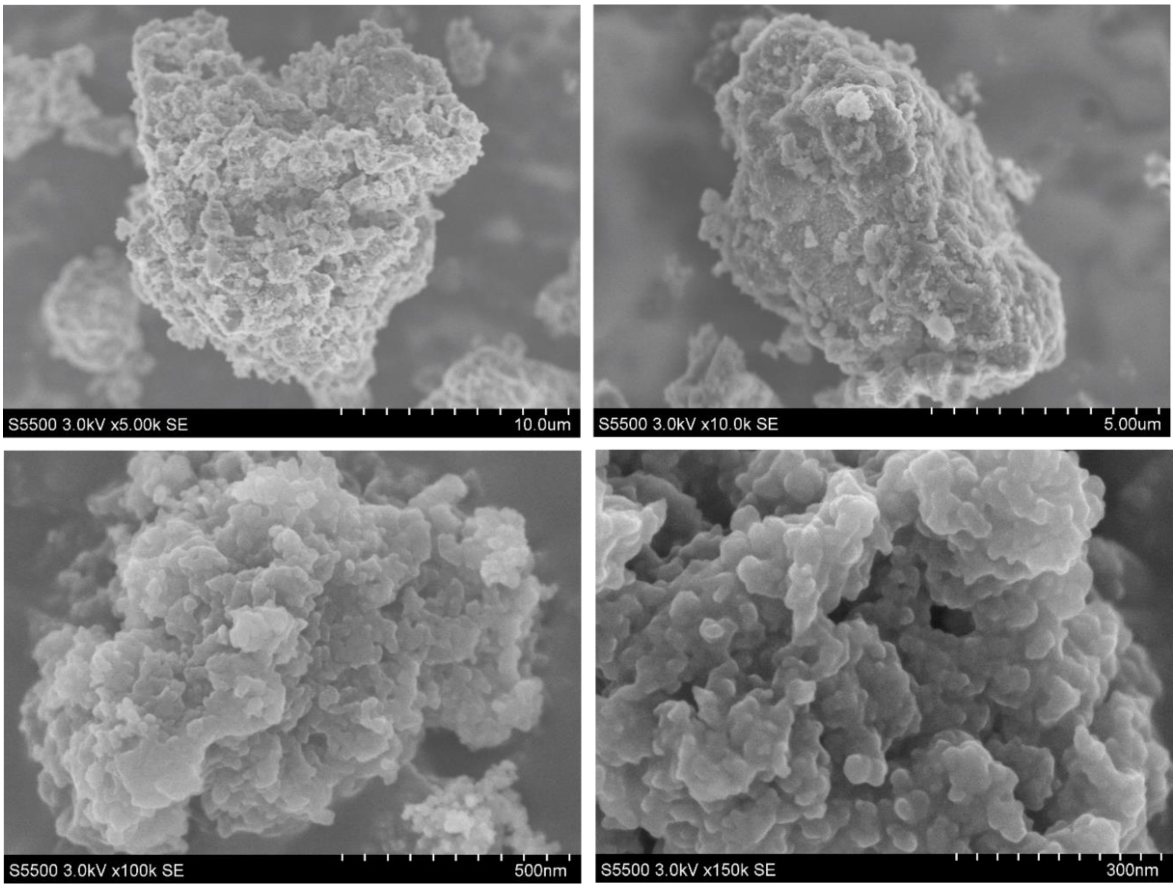

Figure S38. SEM images of **BP1**.

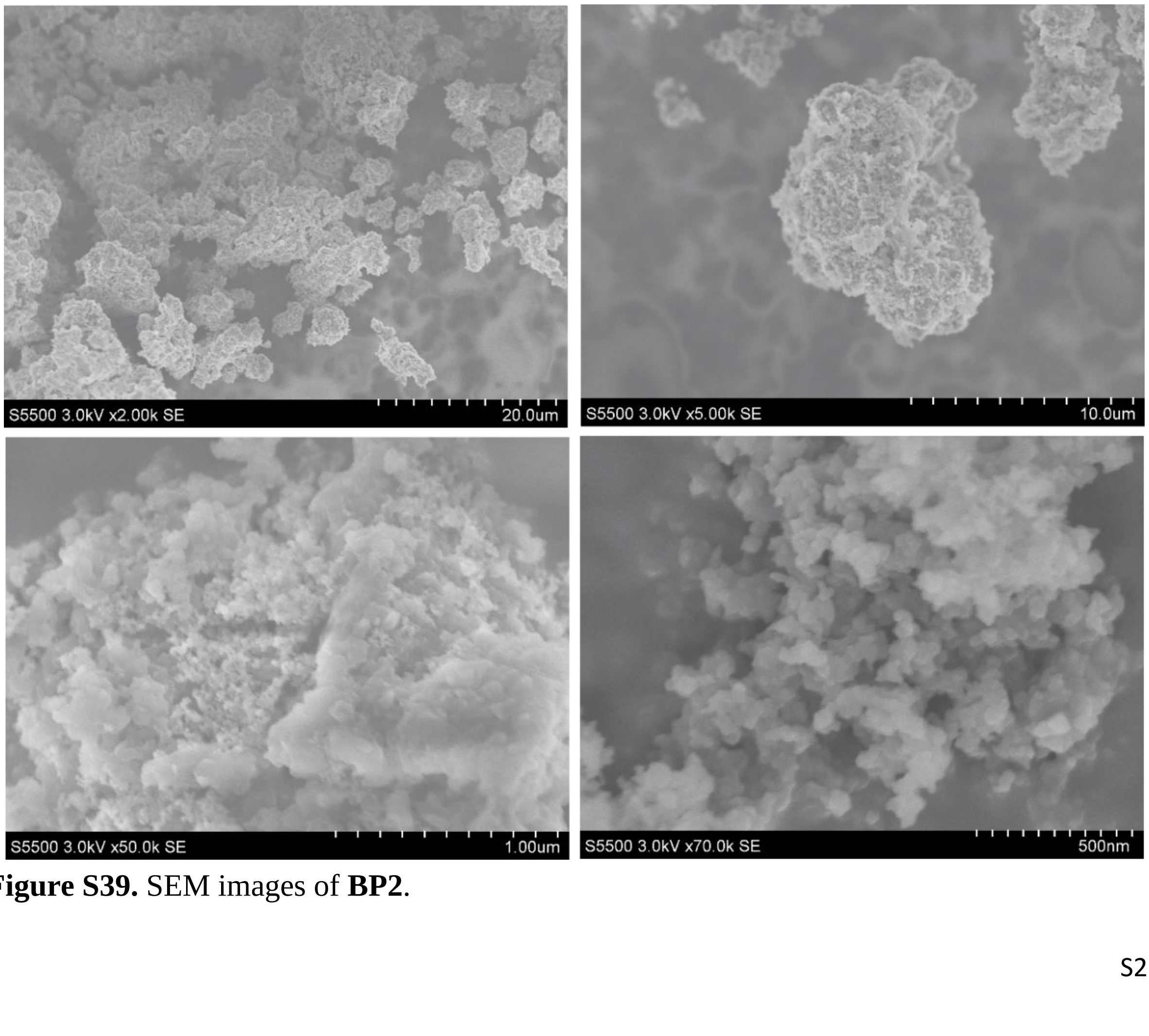

Figure S39. SEM images of **BP2**.

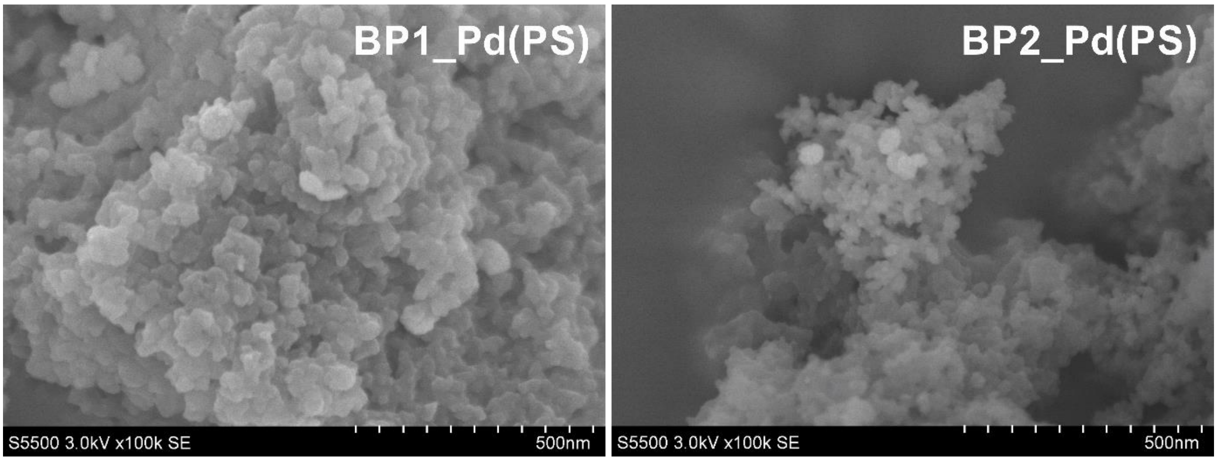

Figure S40. SEM images of **BP1-Pd(PS)** and **BP2-Pd(PS)**.

8. Single-crystal X-ray diffraction analysis of 2

The single crystal of **2** was measured at 100 K on SuperNova diffractometer equipped with Atlas detector (Cu-K_α radiation, λ = 1.54184 Å). Data reduction and analysis were carried out with the *CrysAlisPro* program.[7] The structure was solved by direct methods using *SHELXS-97*[8] and refined using *SHELXL-2014*.[9] All non-hydrogen atoms were refined anisotropically. The structure is of moderate quality, which is due to the fact that sample crystalizes in a form of very tiny plates. The high values of R and wR factors result also from the presence of unrefinement residual electron density, which is located in the central part of channels. It can be attributed to the highly disordered solvent molecule. Crystallographic Information File (CIF) has been deposited with the Cambridge Crystallographic Data Centre as supplementary publications no. 1994974.

9. Sorption measurements

Micromeritics ASAP 2020 Surface Area and Porosity Analyzer was used to measure the nitrogen adsorption isotherms. Oven-dried samples were placed in tared tubes with/equipped with filler rod, capped with SealFrit stopper. Samples were activated at maximum temperature of 150 °C in vacuo (0.002 Torr) for 24 h. N_2 isotherms were measured using liquid nitrogen baths (77 K). High purity grade N_2, H_2, CO_2, CH_4 and He, oil-free valves, and gas regulators were used for the free space correction and measurement. Relative pressure (P/P_0) range for BET analysis was selected based on Rouquerol's criteria.[10]

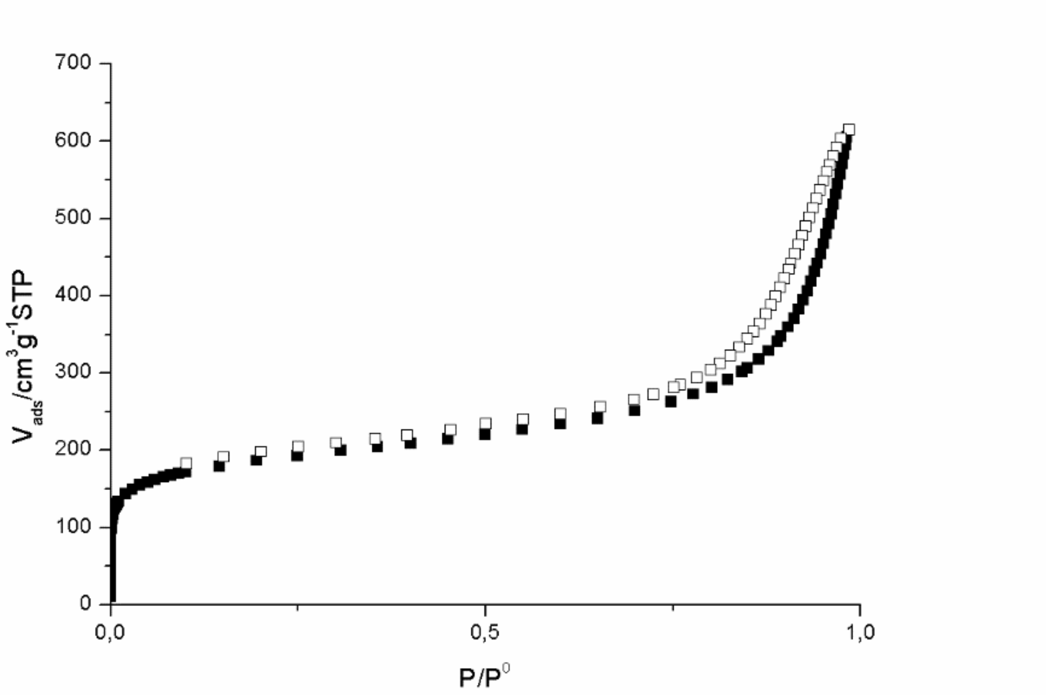

Figure S41. N_2@77K sorption isotherm for **BP1**

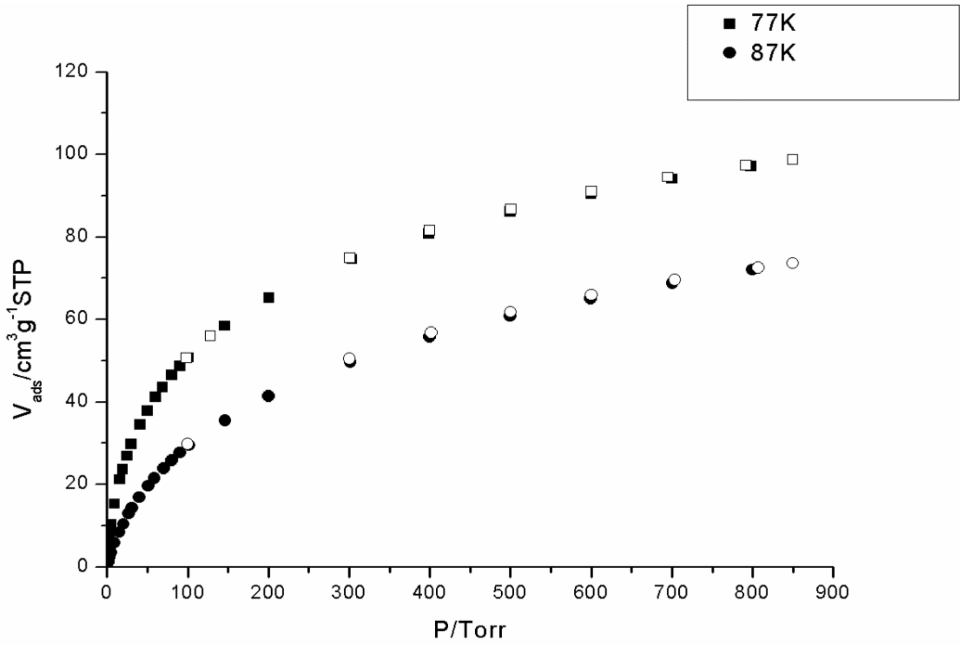

Figure S42. H_2 sorption isotherms for **BP1**

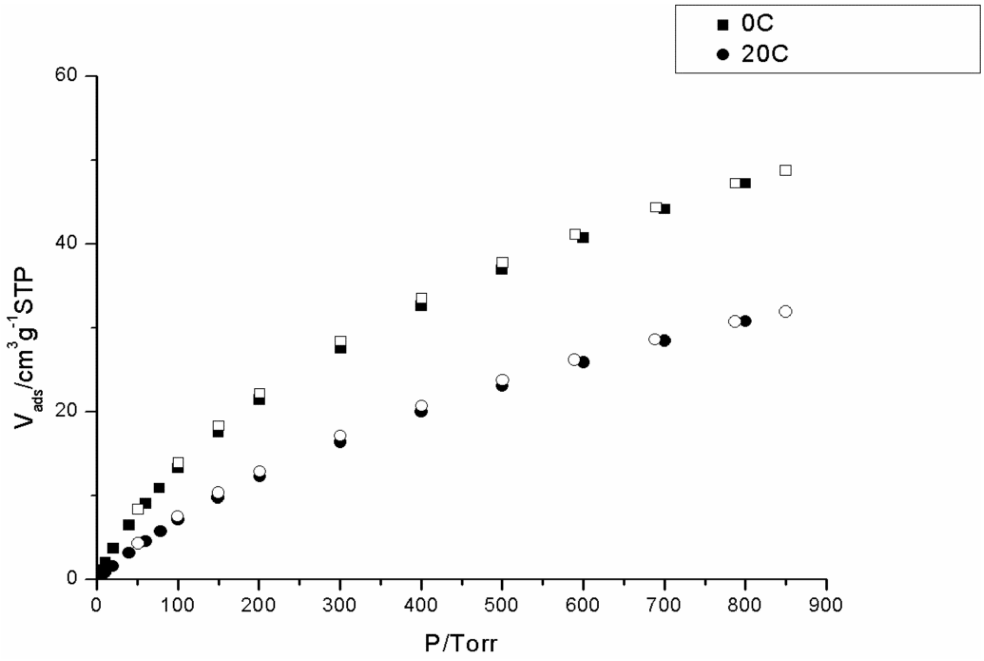

Figure S43. CO_2 sorption isotherms for **BP1**

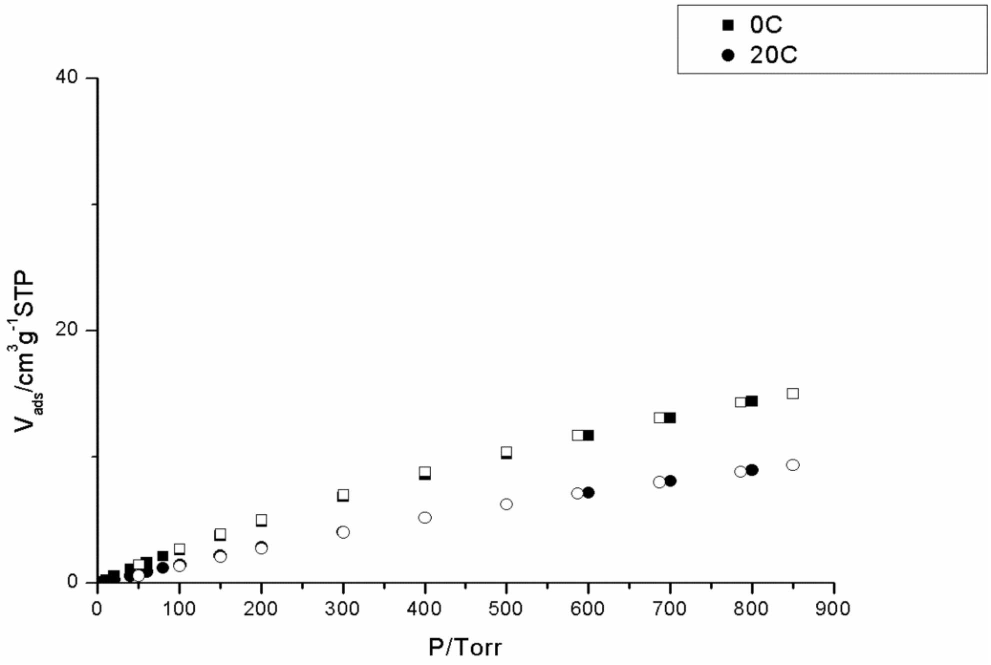

Figure S44. CH_4 sorption isotherms for **BP1**

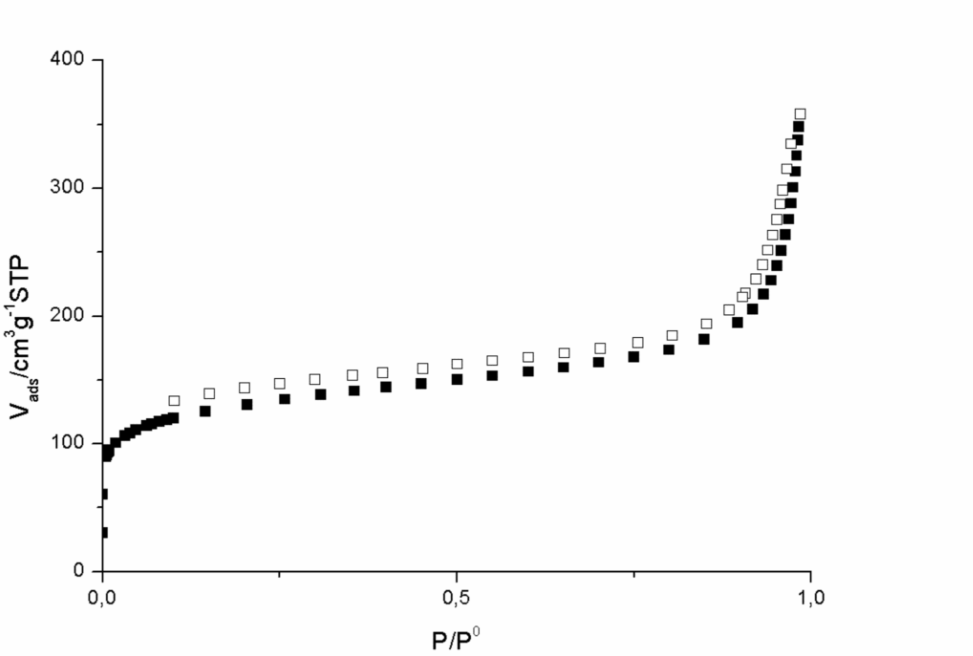

Figure S45. N_2@77K sorption isotherm for **BP2**

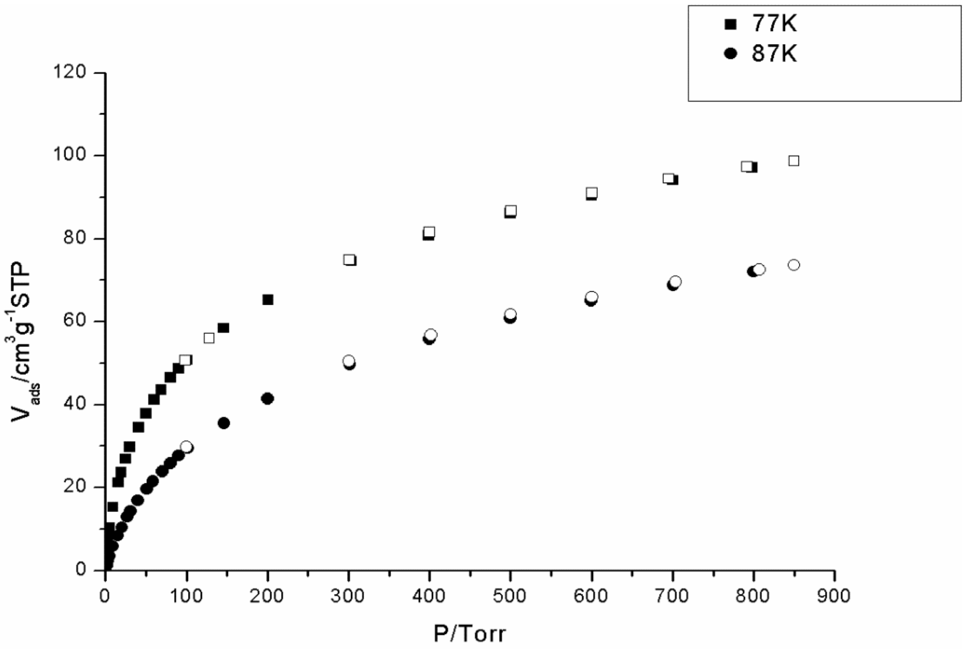

Figure S46. H_2 sorption isotherms for **BP2**

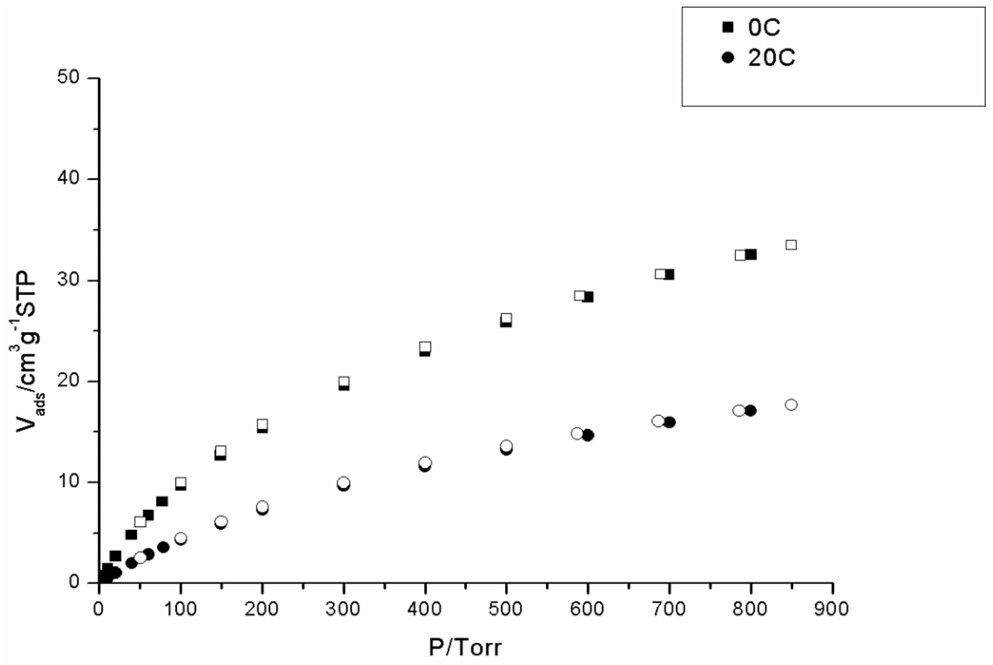

Figure S47. CO_2 sorption isotherms for **BP2**

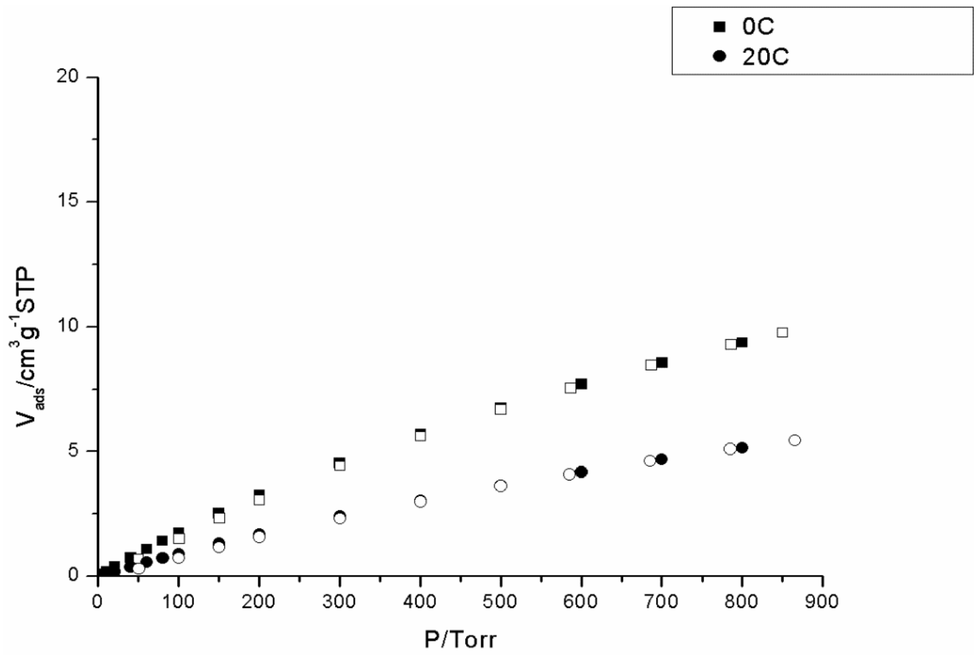

Figure S48. CH_4 sorption isotherms for **BP2**

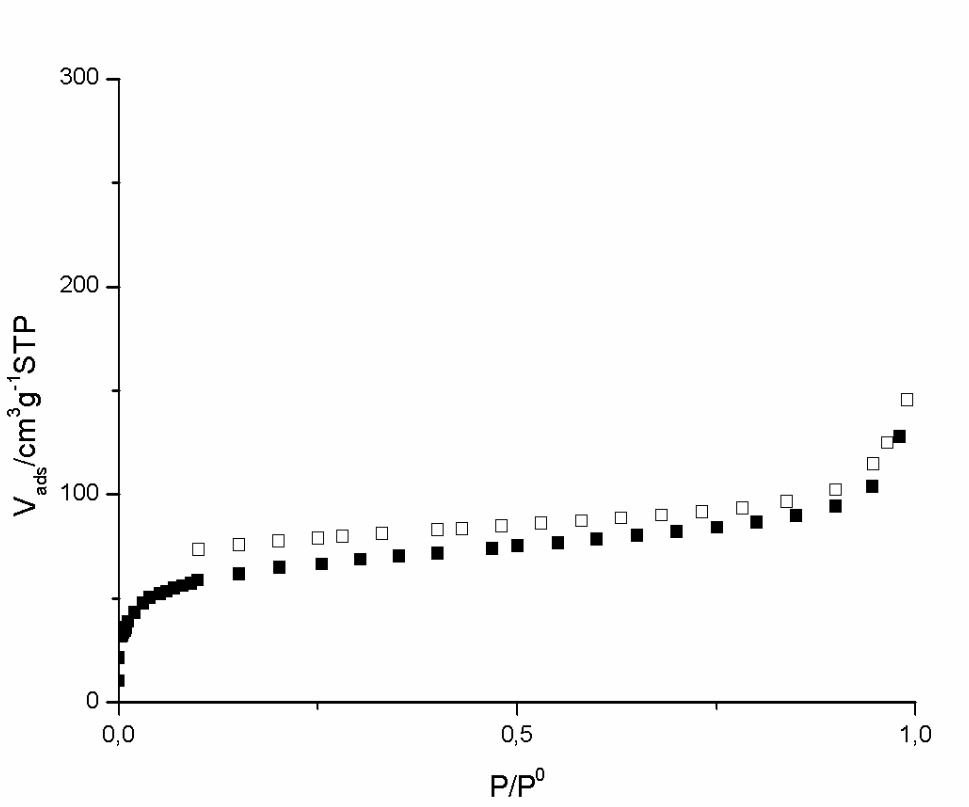

Figure S49. N_2@77K sorption isotherm for **BPO1**

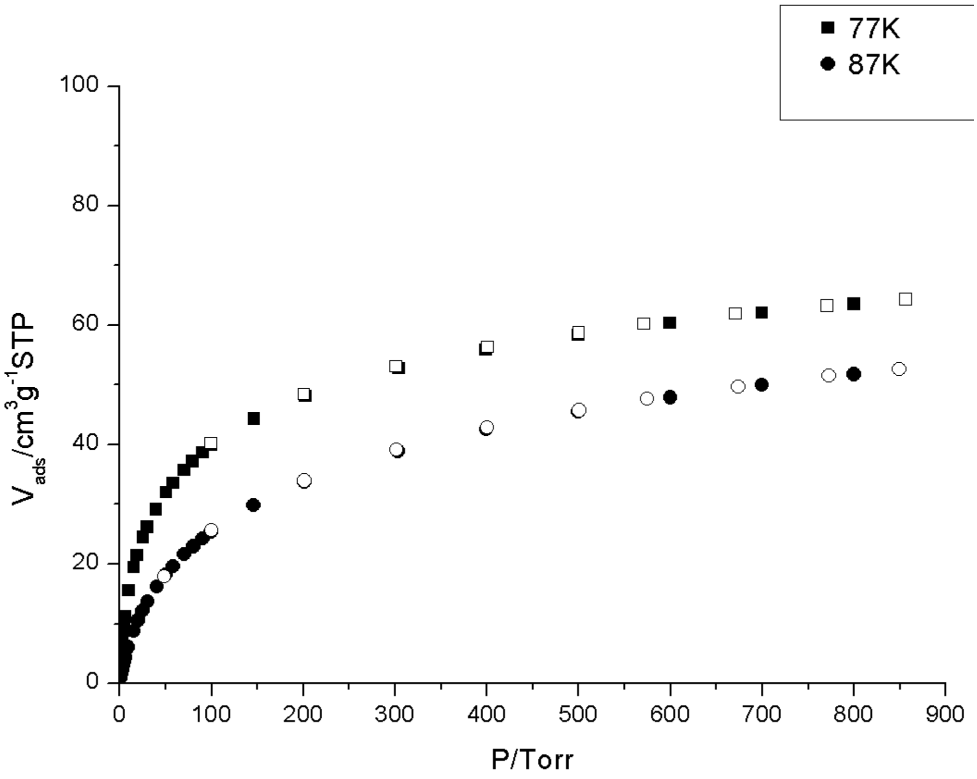

Figure S50. H_2 sorption isotherms for **BPO1**

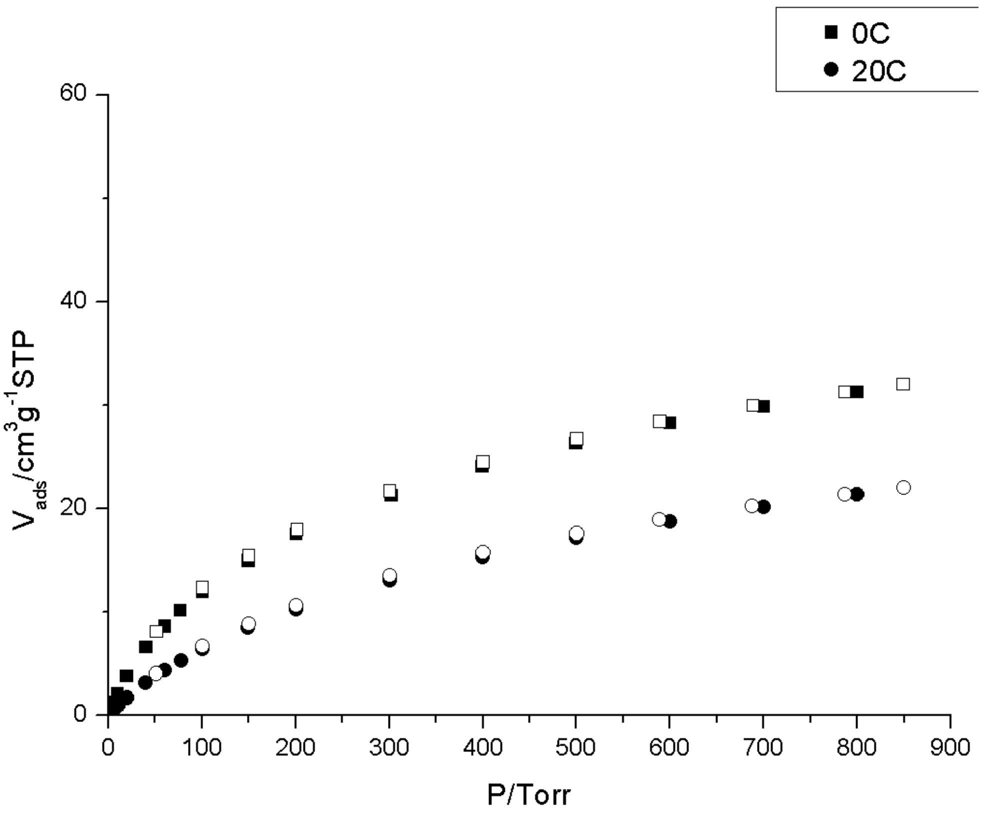

Figure S51. CO_2 sorption isotherms for **BPO1**

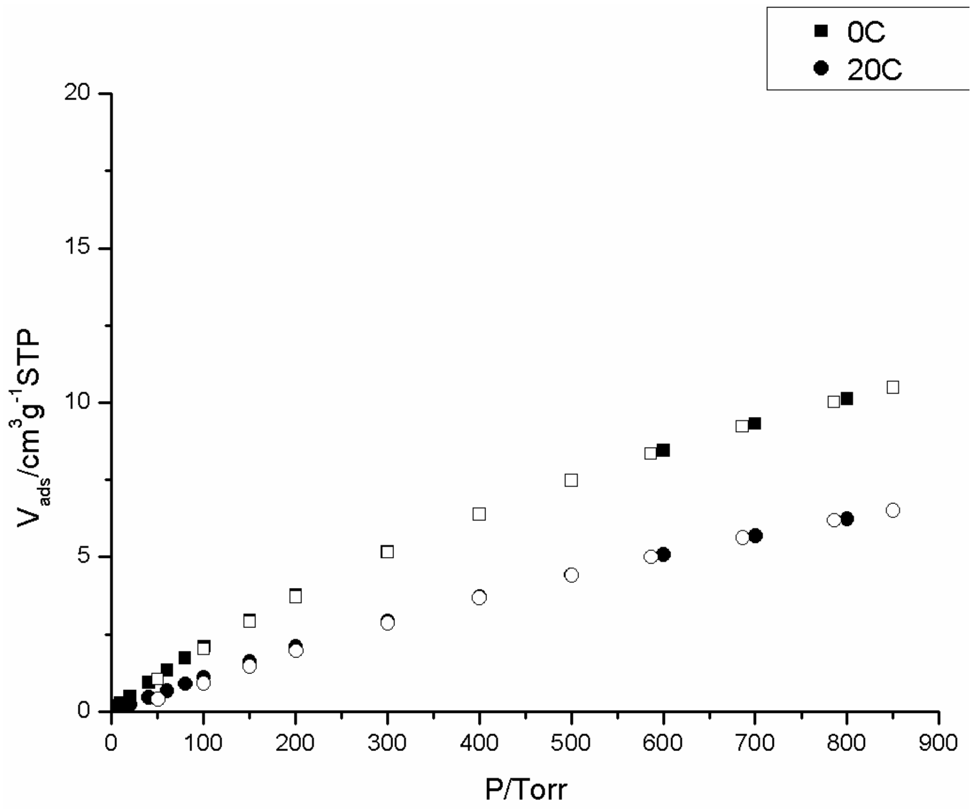

Figure S52. CH_4 sorption isotherms for **BPO1**

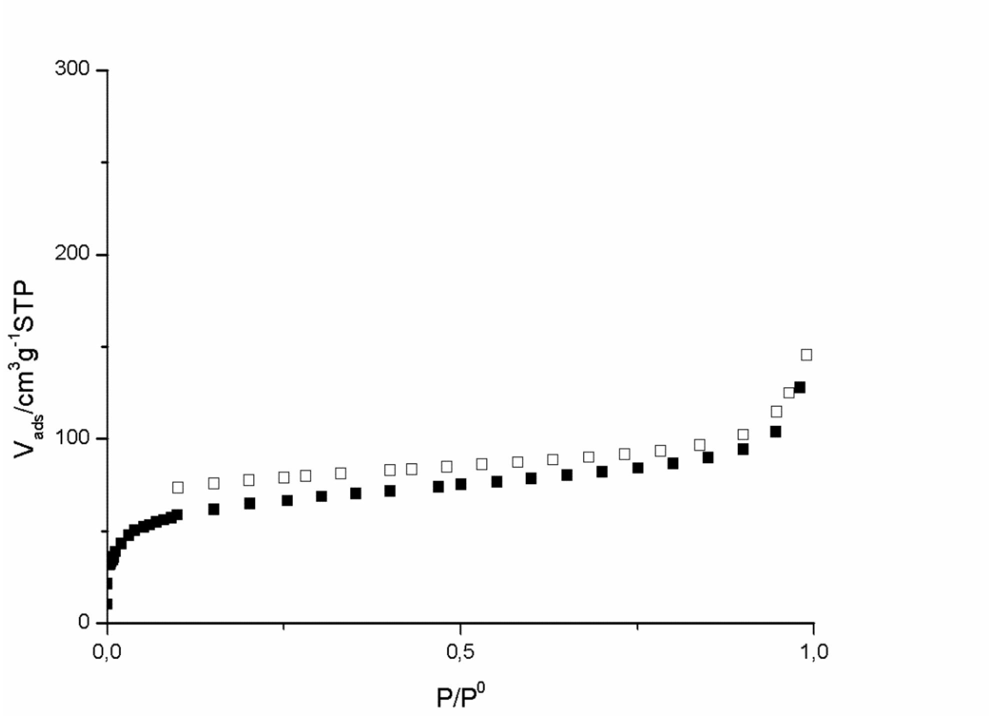

Figure S53. N_2@77K sorption isotherm for **BPO2**

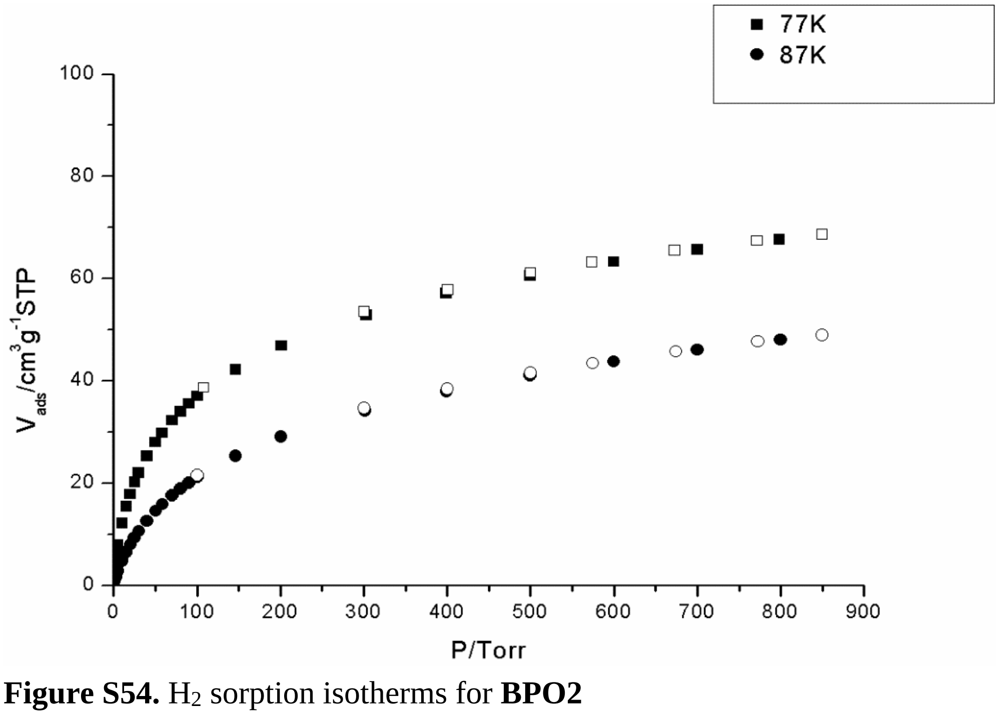

Figure S54. H_2 sorption isotherms for **BPO2**

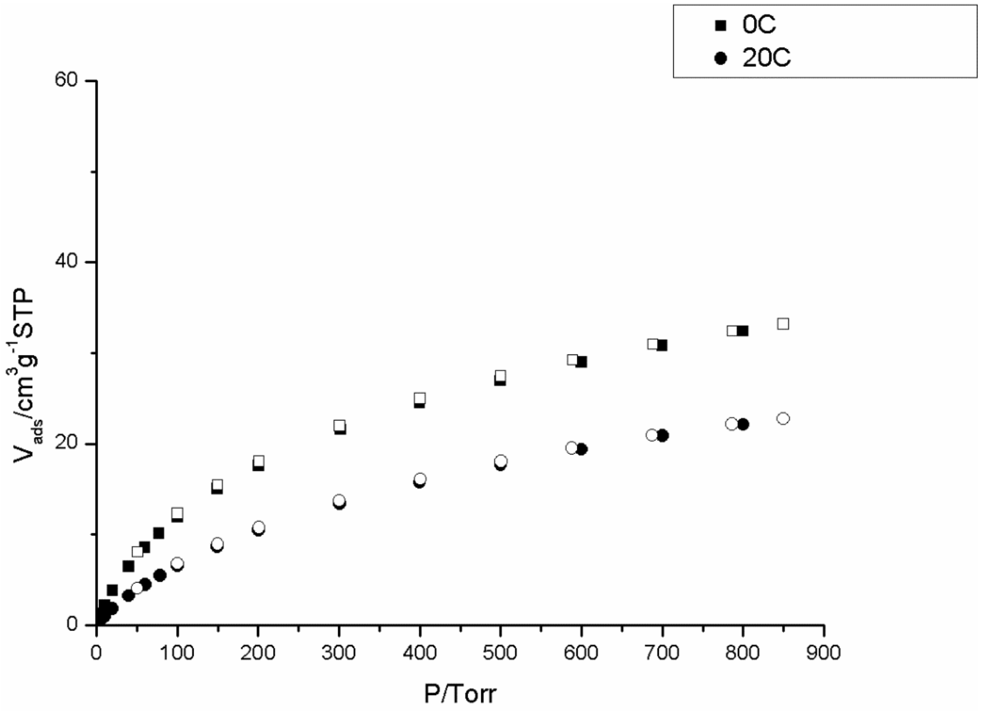

Figure S55. CO_2 sorption isotherms for **BPO2**

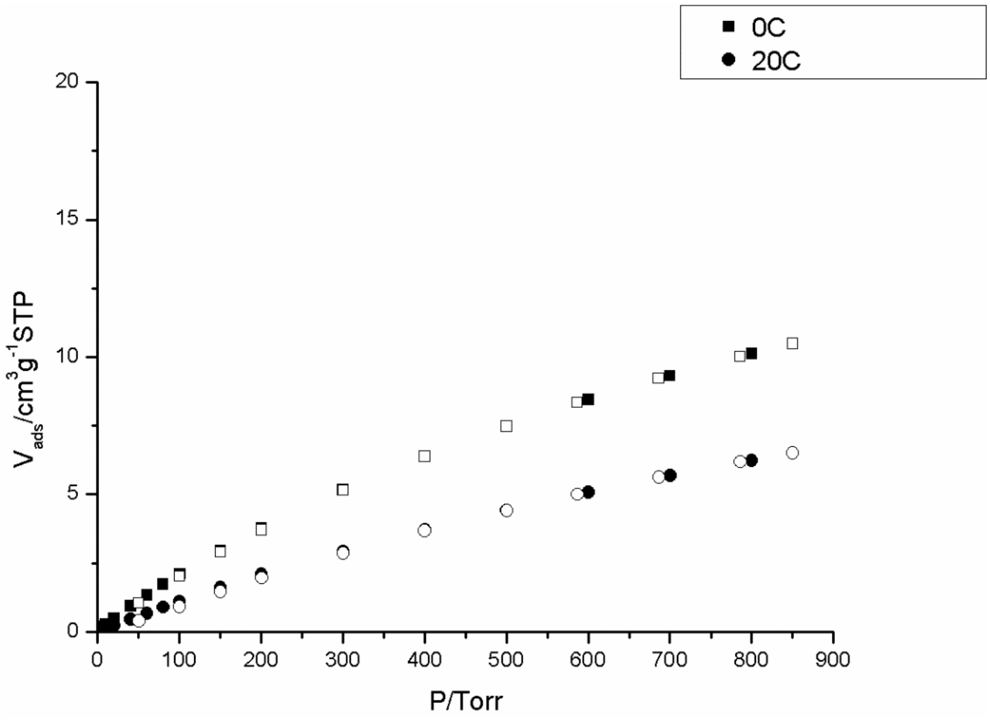

Figure S56. CH_4 sorption isotherms for **BPO2**

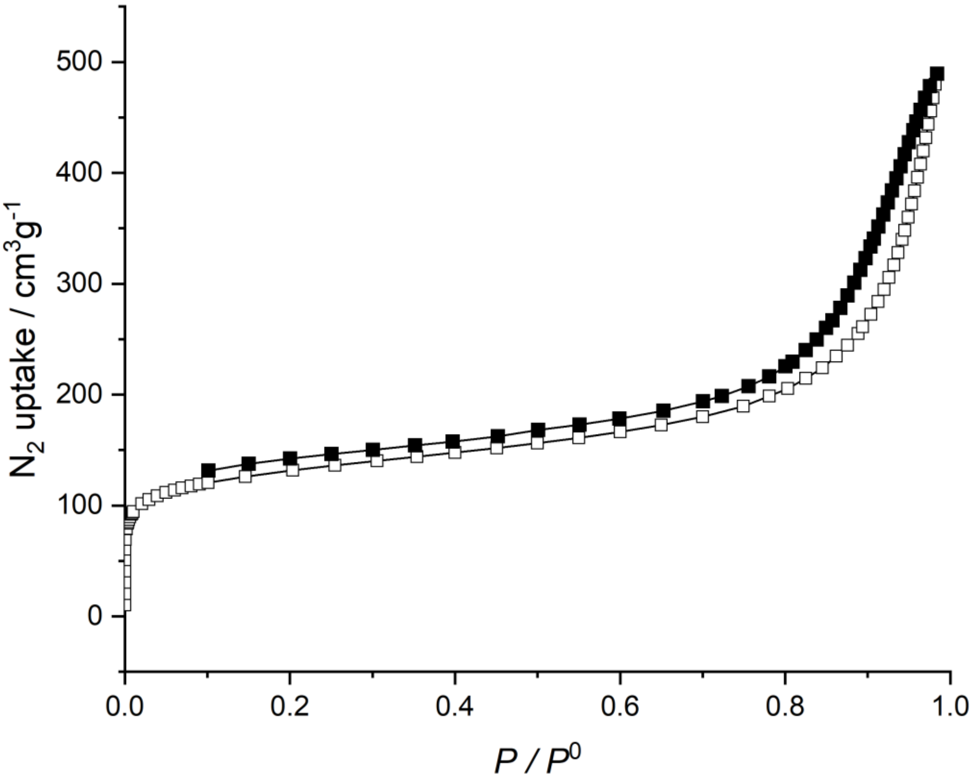

Figure S57. N_2@77K sorption isotherm for **BP1-Pd(BU)**

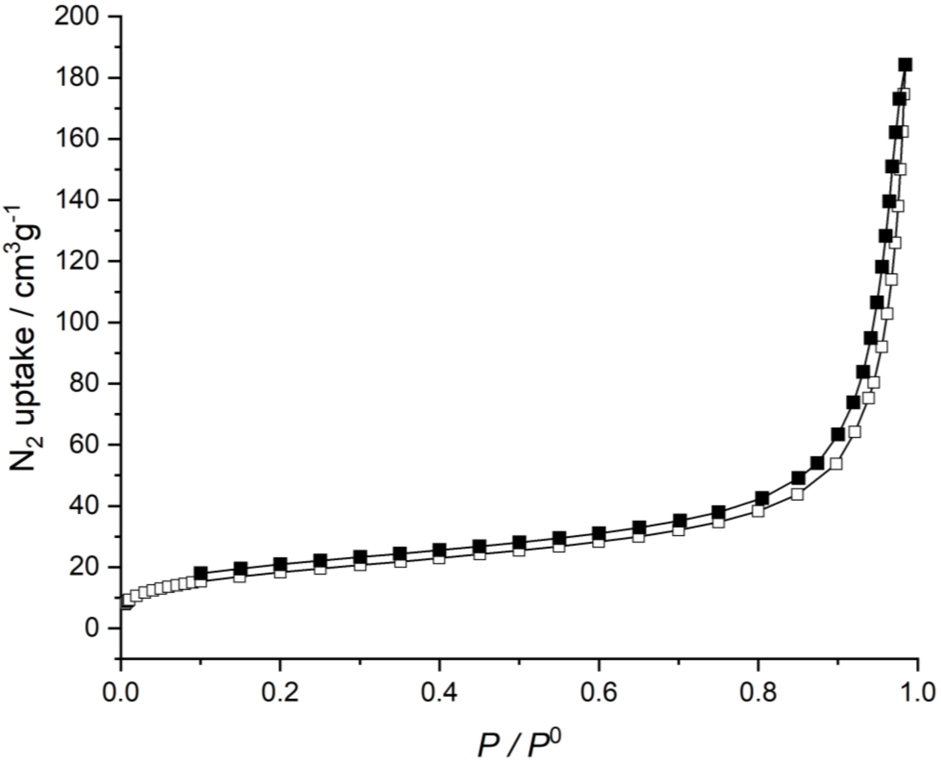

Figure S58. N_2@77K sorption isotherm for **BP2-Pd(BU)**

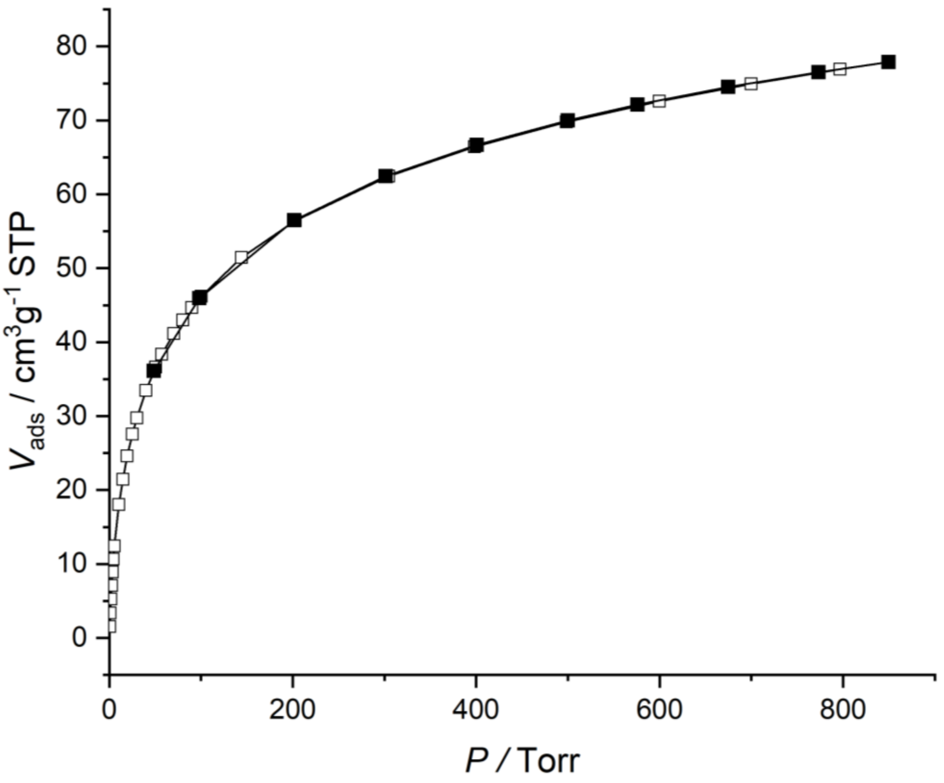

Figure S59. H_2 sorption isotherms for **BP2-Pd(BU)**

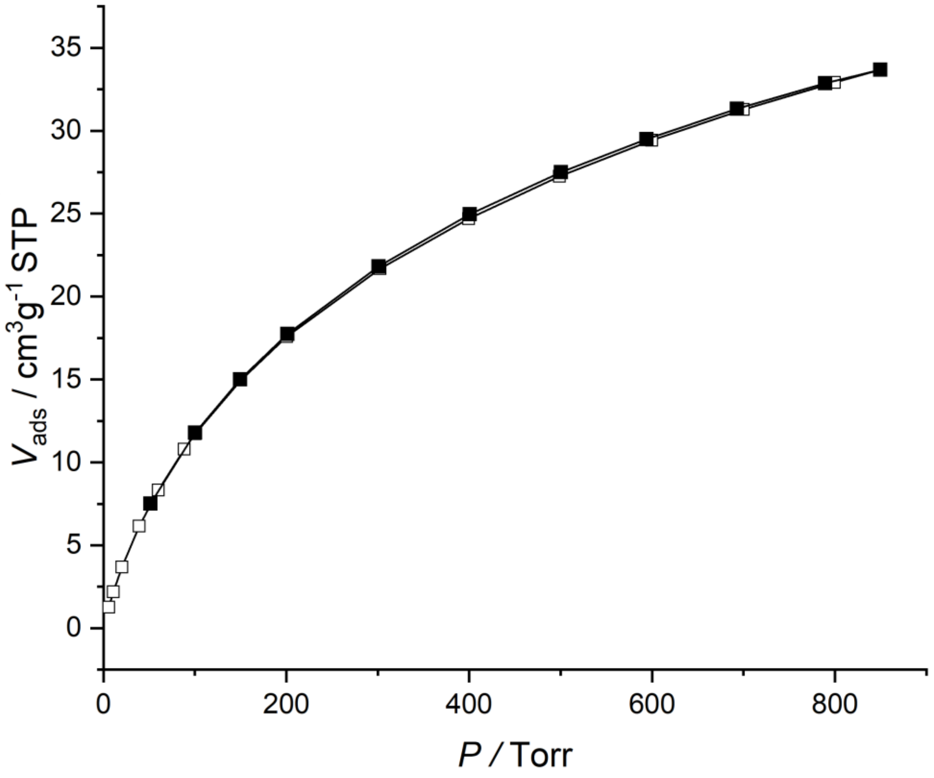

Figure S60. CO_2 sorption isotherms for **BP2-Pd(BU)**

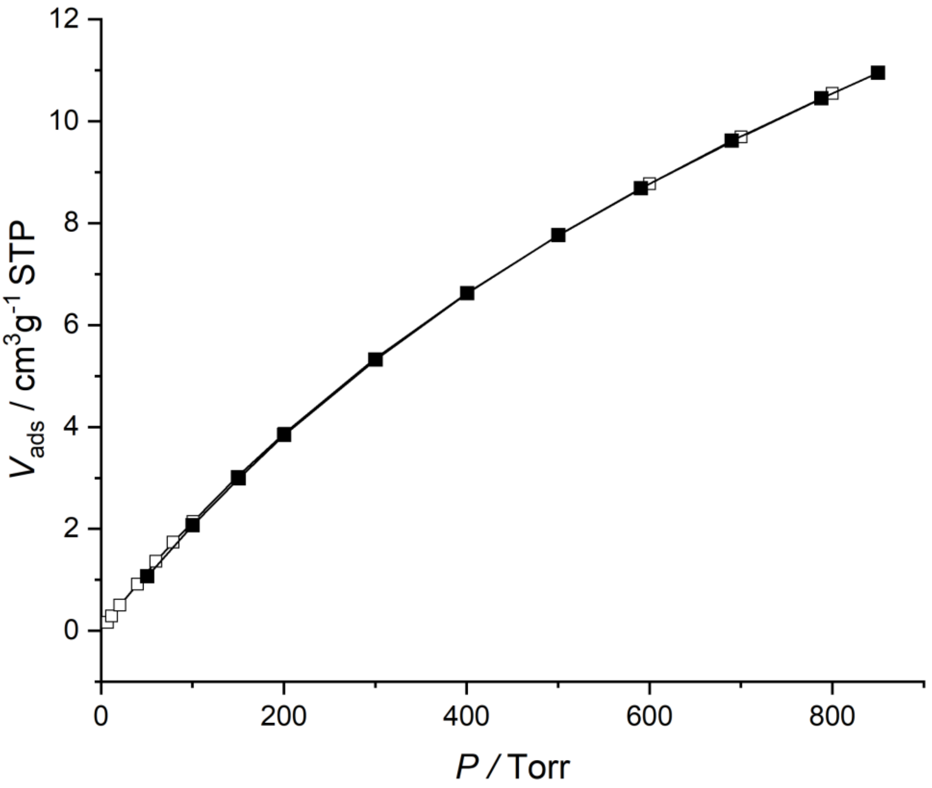

Figure S61. CH_4 sorption isotherms for **BP2-Pd(BU)**

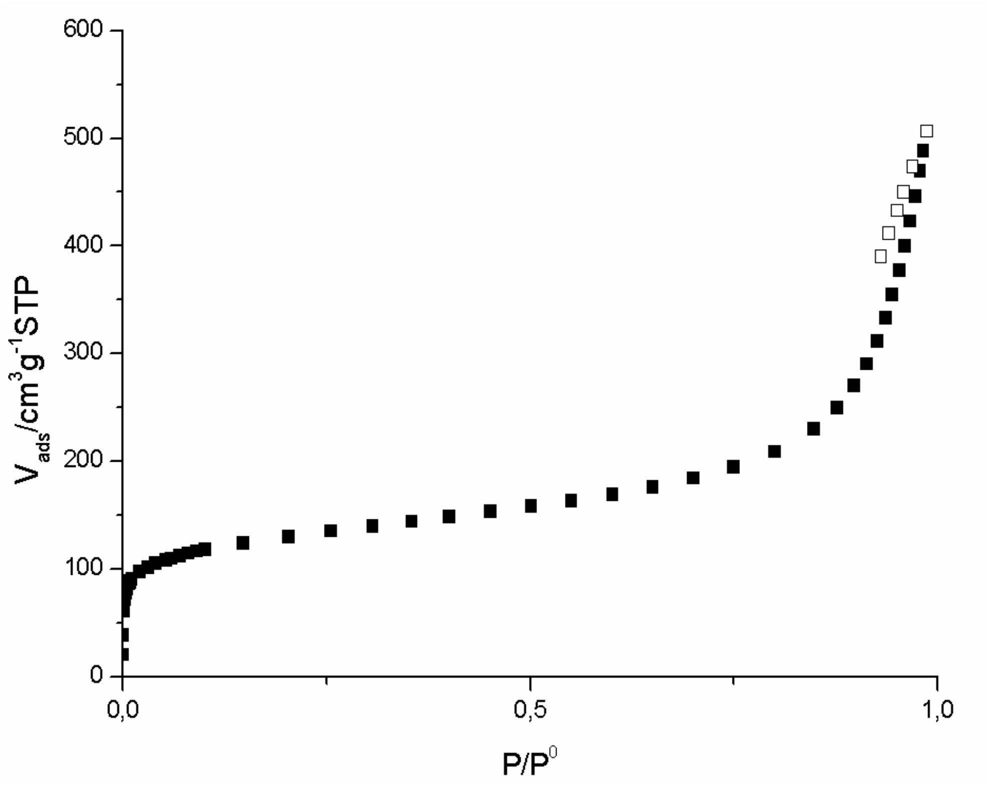

Figure S62. N_2@77K sorption isotherm for **BP1-Pd(PS)**

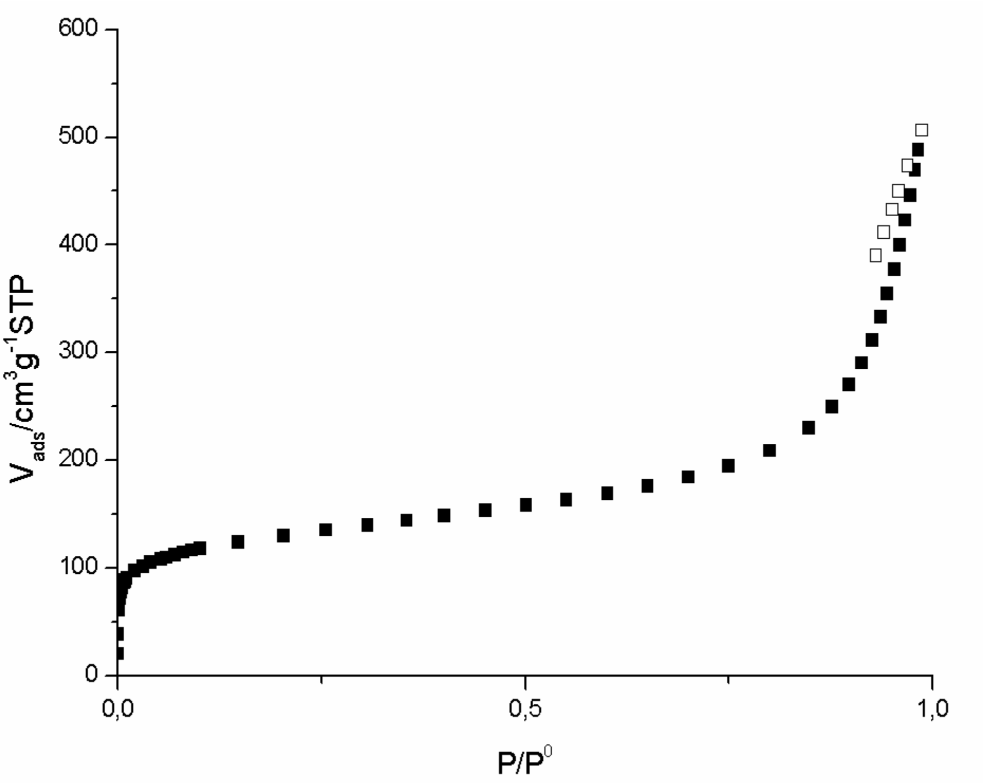

Figure S63. H_2 sorption isotherms for **BP1-Pd(PS)**

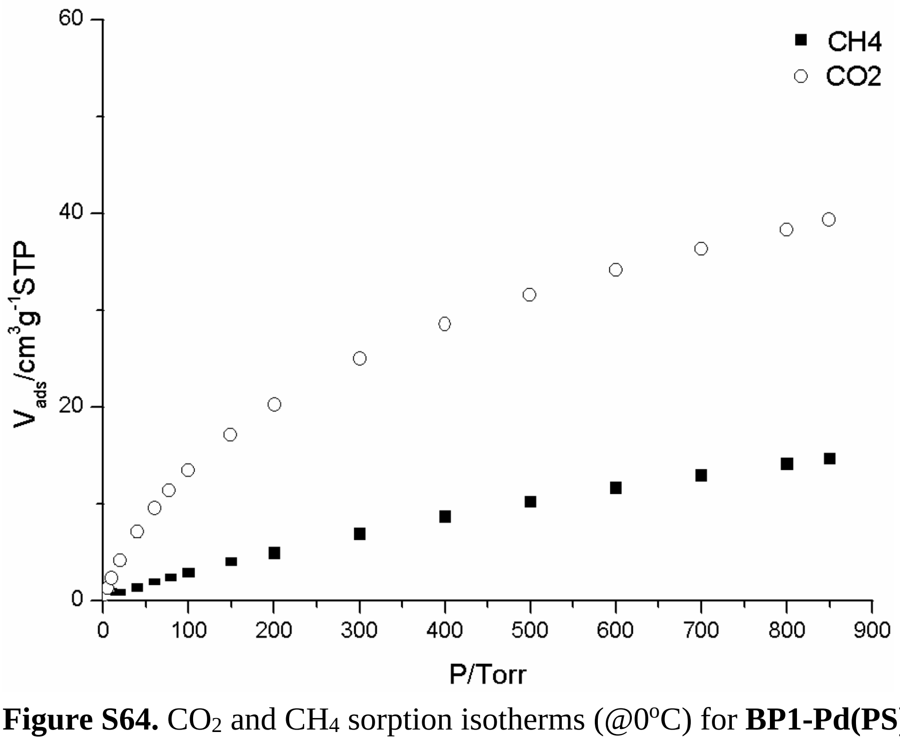

Figure S64. CO_2 and CH_4 sorption isotherms (@0°C) for **BP1-Pd(PS)**

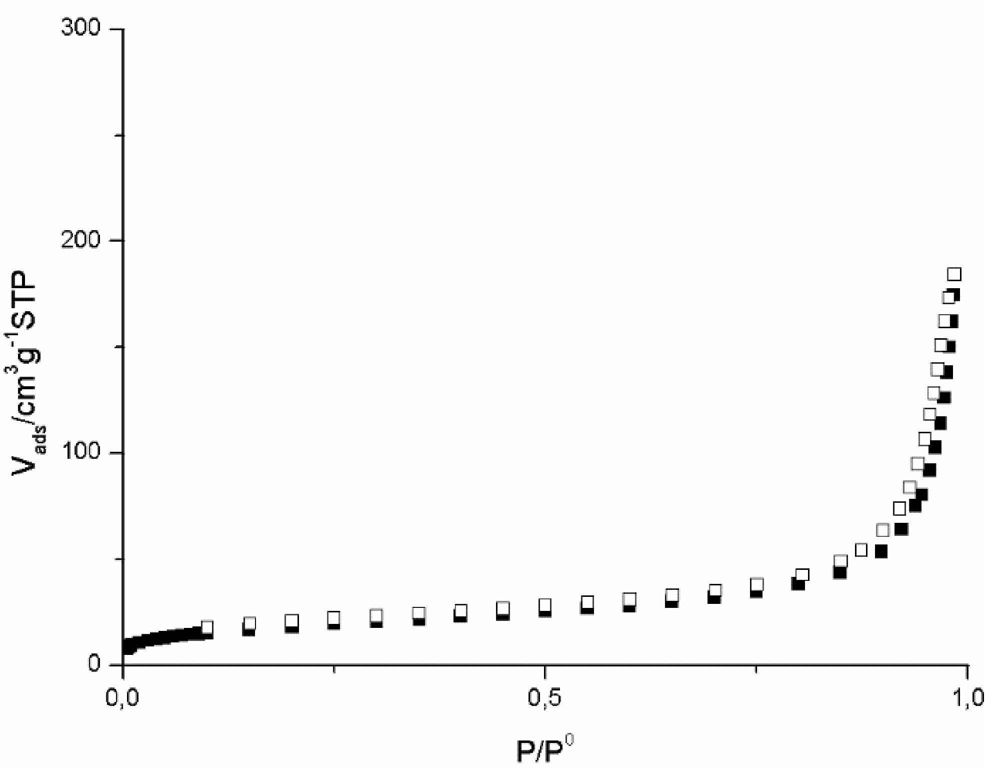

Figure S65. N_2@77K sorption isotherm for **BP2-Pd(PS)**

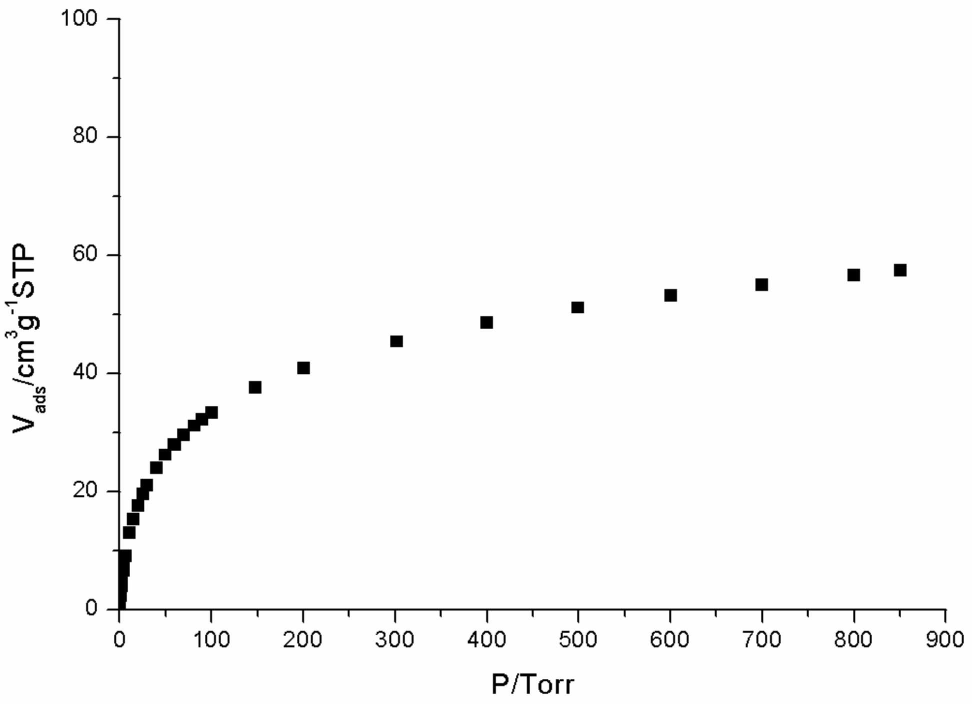

Figure S66. H_2 sorption isotherms for **BP2-Pd(PS)**

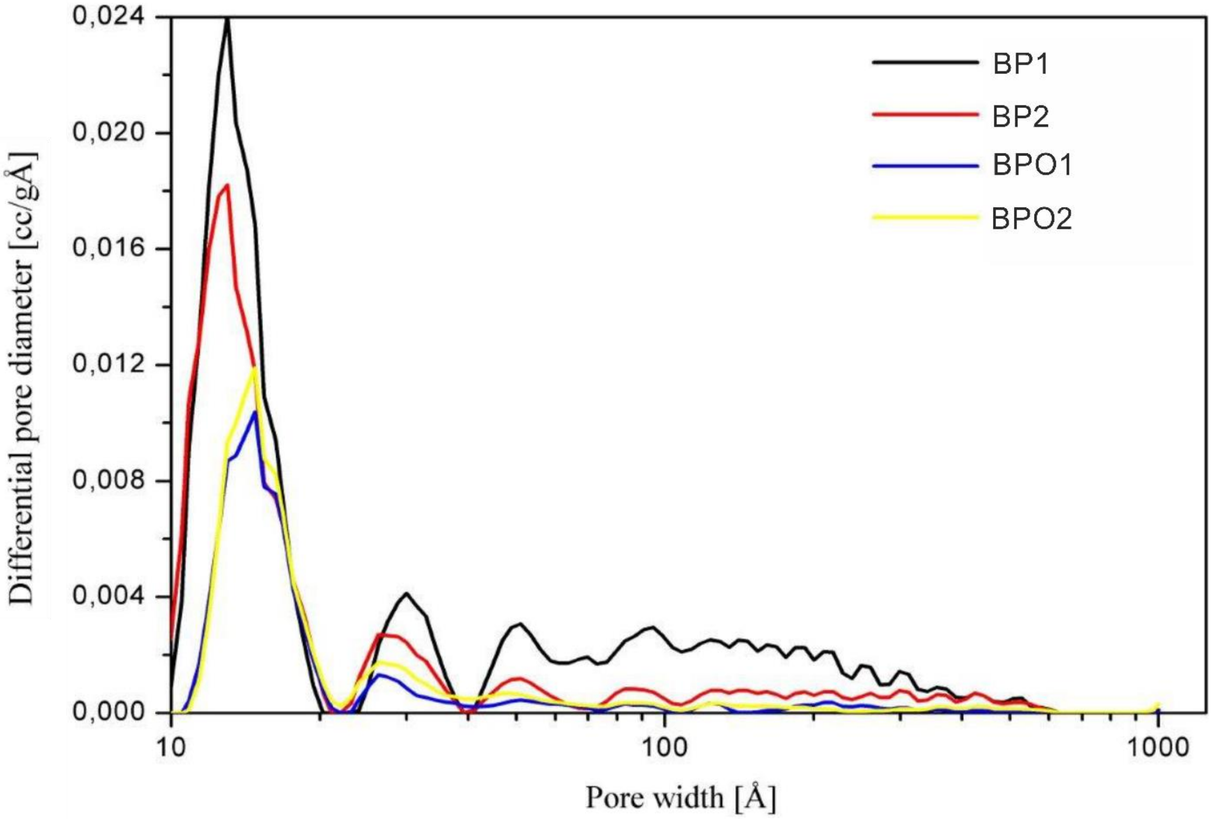

Figure S67. Comparison of pore size distribution in **BP1-2** and **BPO1-2** based on N_2@77K sorption measurements.

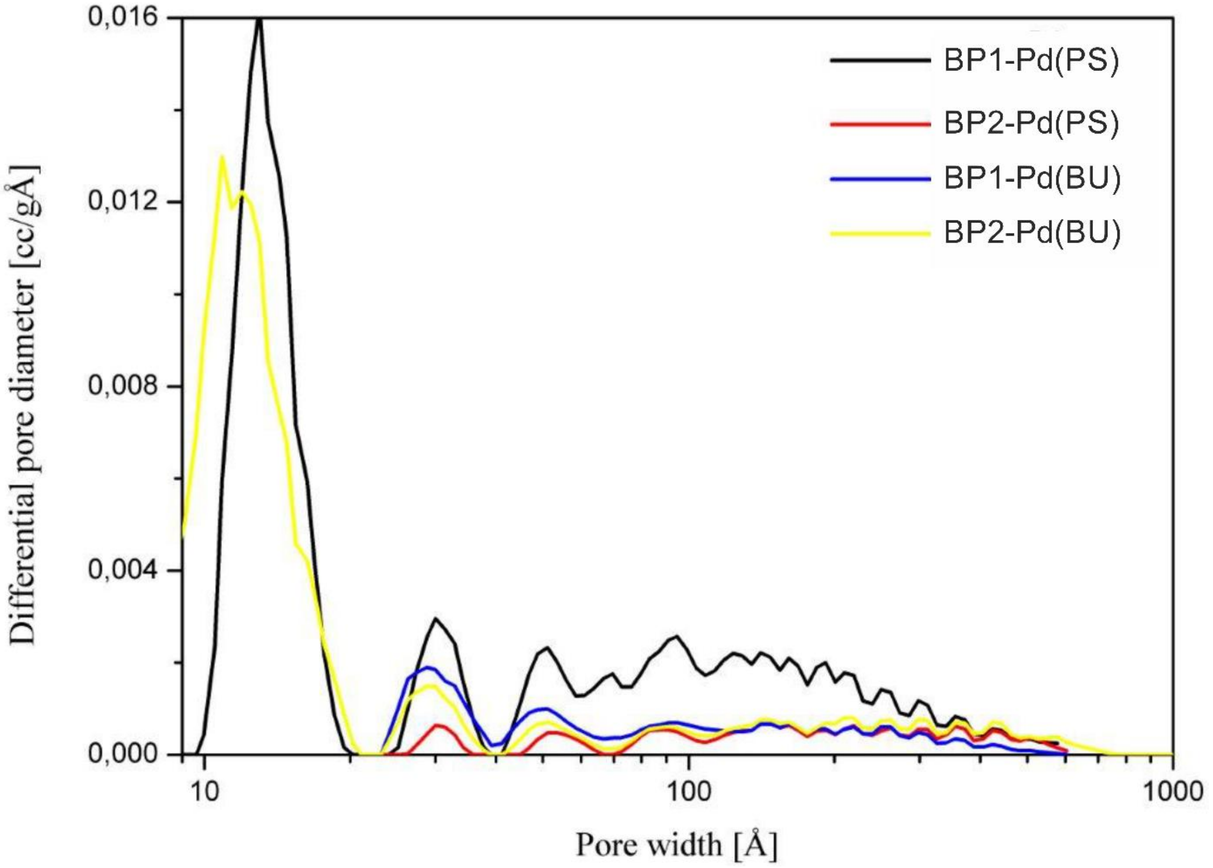

Figure S68. Comparison of pore size distribution in Pd-functionalized COFs based on N_2@77K sorption measurements.

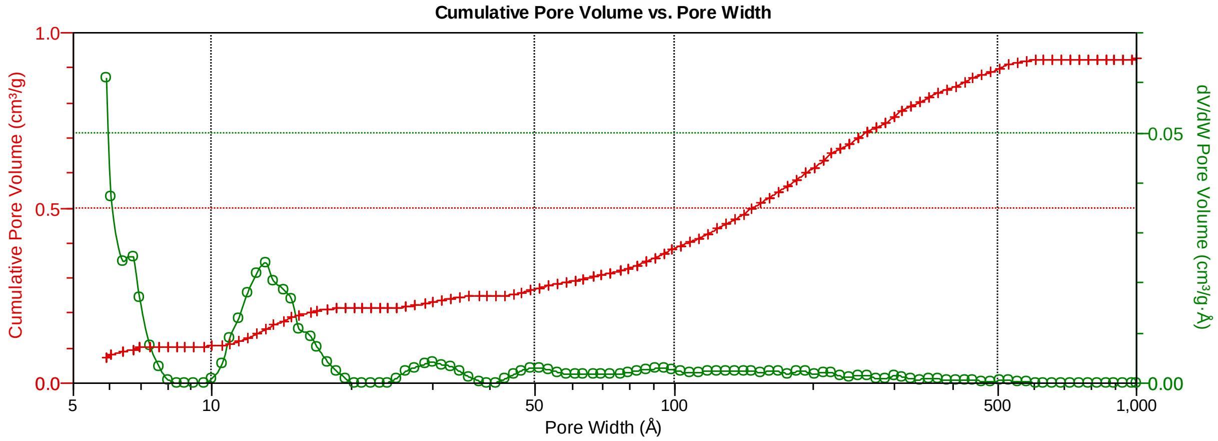

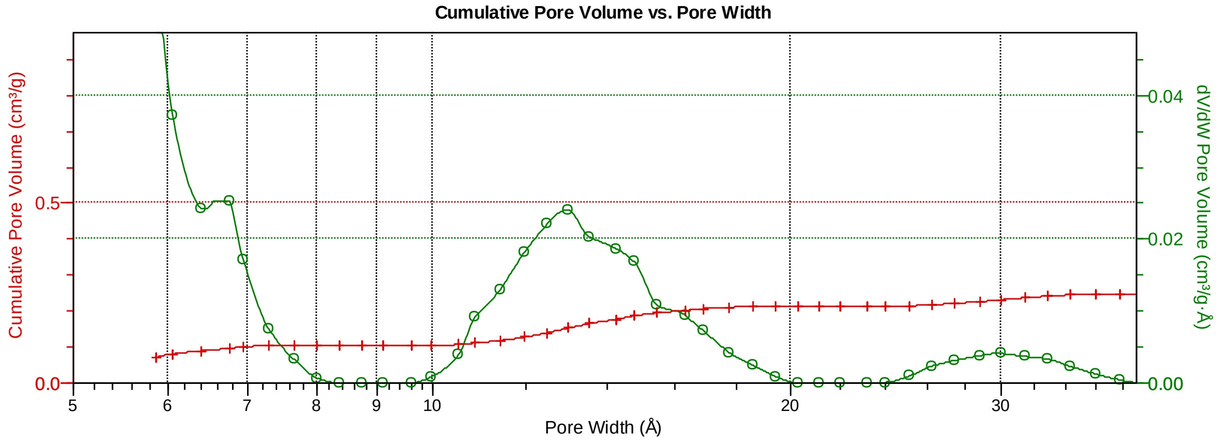

Figure S69. Pore size distribution in **BP1** based on N_2@77K sorption measurement (λ = 0.1000).

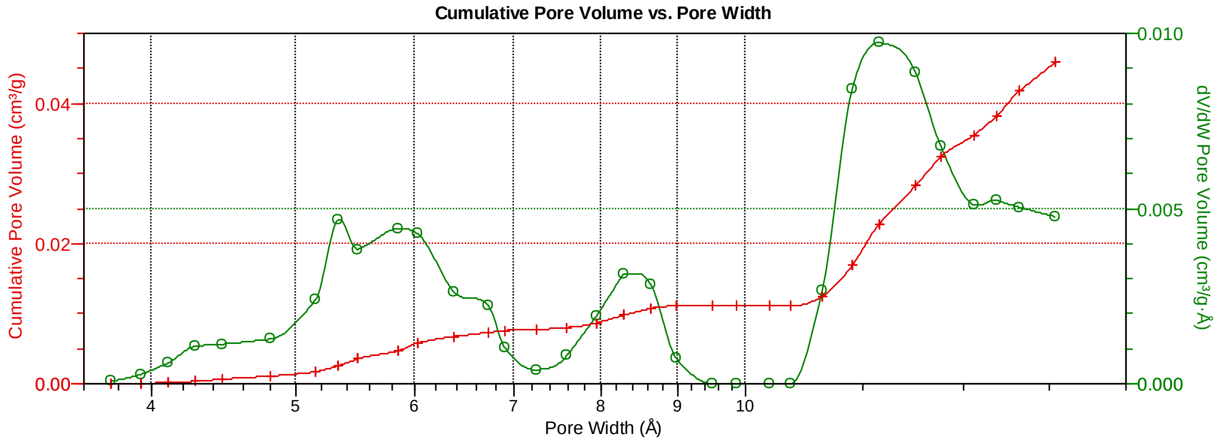

Figure S70. Pore size distribution in **BP1** based on CO_2@0°C sorption measurement (λ = 0.0316).

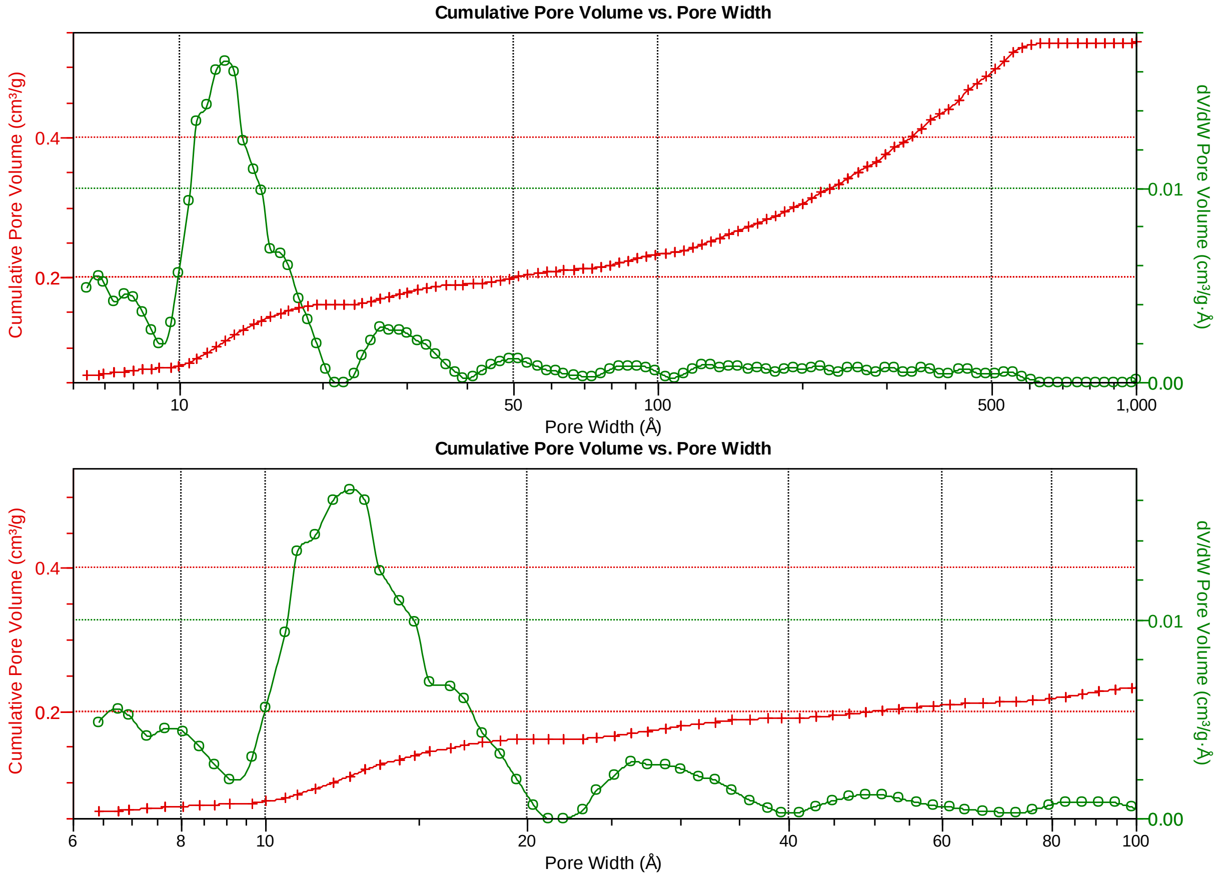

Figure S71. Pore size distribution in **BP2** based on N_2@77K sorption measurement (λ = 0.1000).

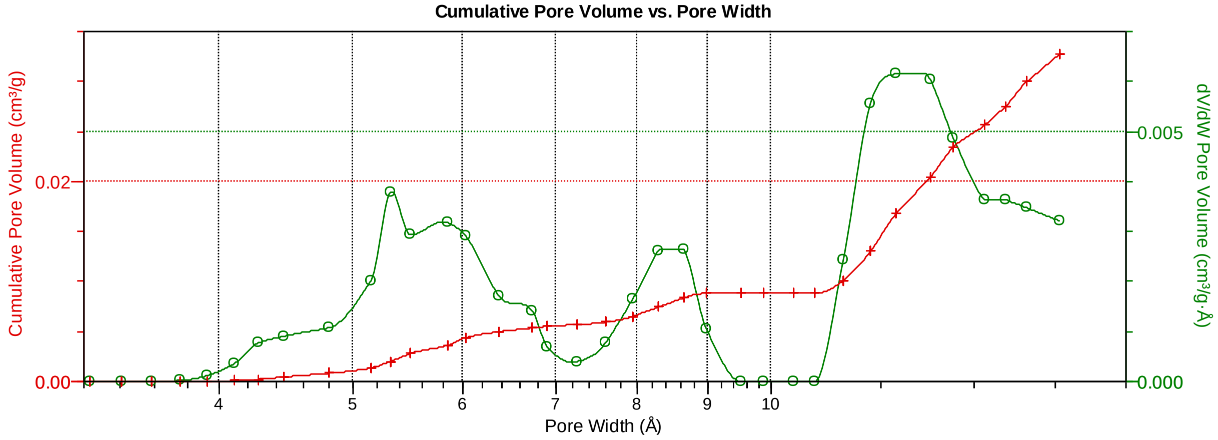

Figure S72. Pore size distribution in **BP2** based on CO_2@0°C sorption measurement (λ = 0.0316).

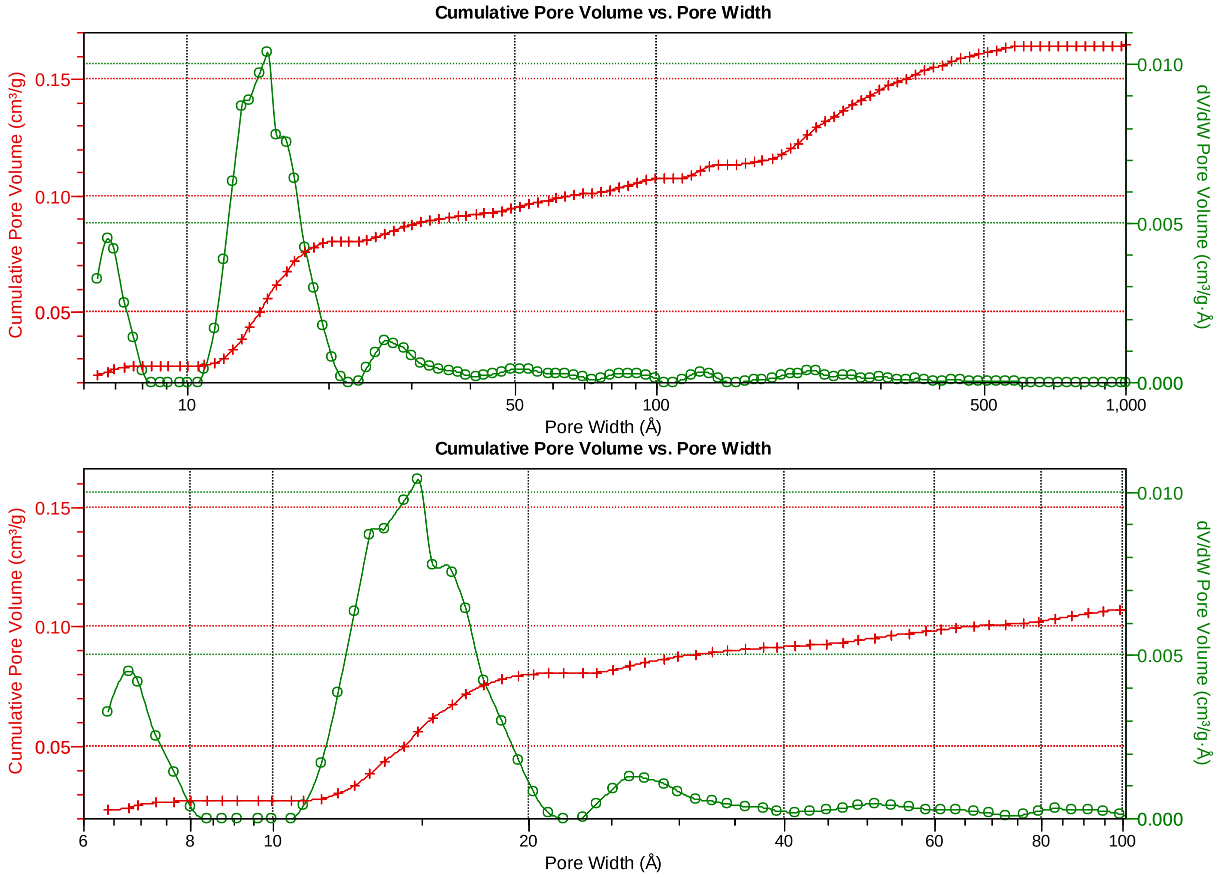

Figure S73. Pore size distribution in **BPO1** based on N_2@77K sorption measurement (λ = 0.1000).

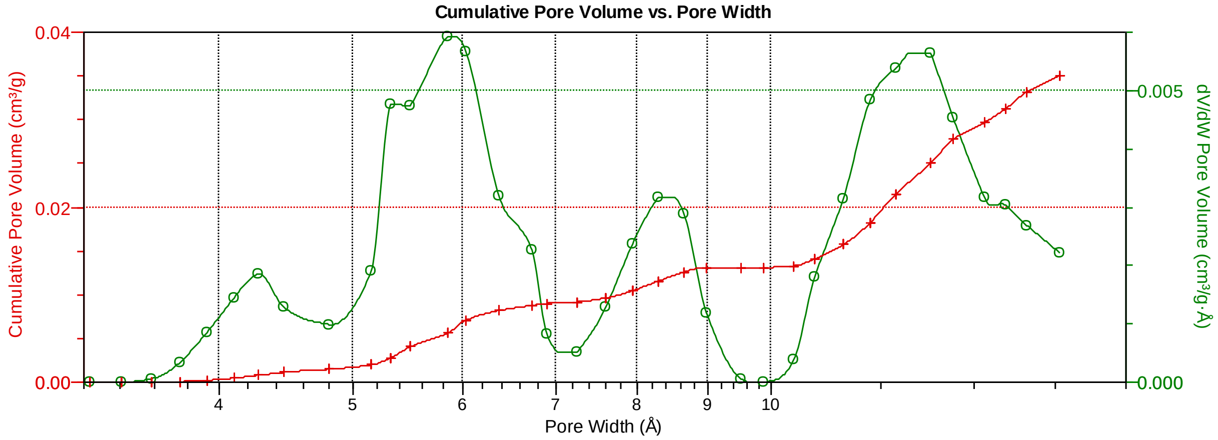

Figure S74. Pore size distribution in **BPO1** based on CO_2@0°C sorption measurement (λ = 0.0316).

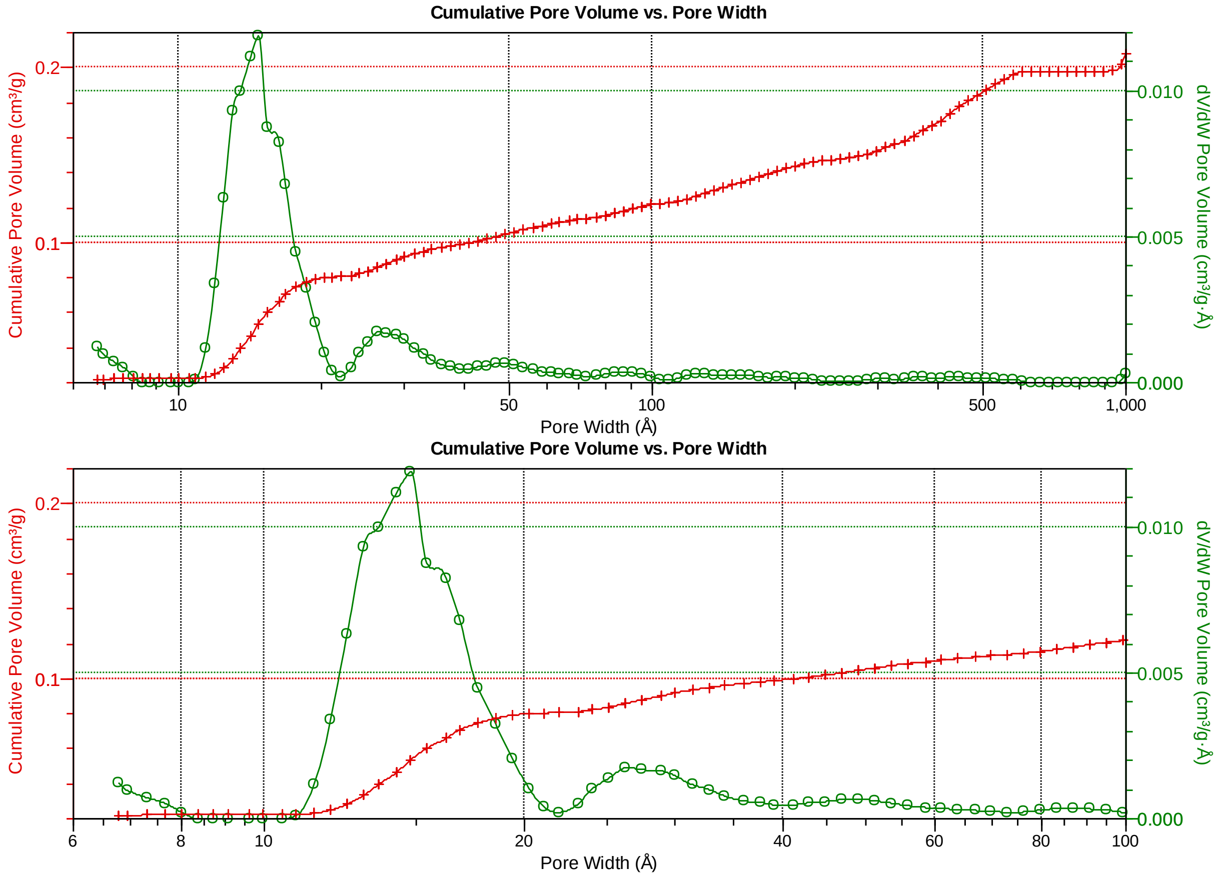

Figure S75. Pore size distribution in **BPO2** based on N_2@77K sorption measurement (λ = 0.1000).

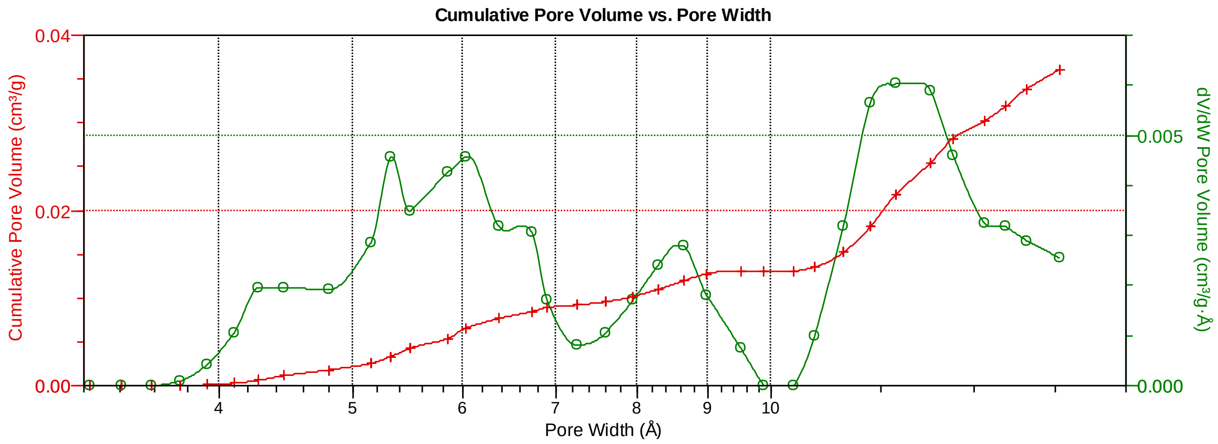

Figure S76. Pore size distribution in **BPO2** based on CO_2@0°C sorption measurement (λ = 0.0316).

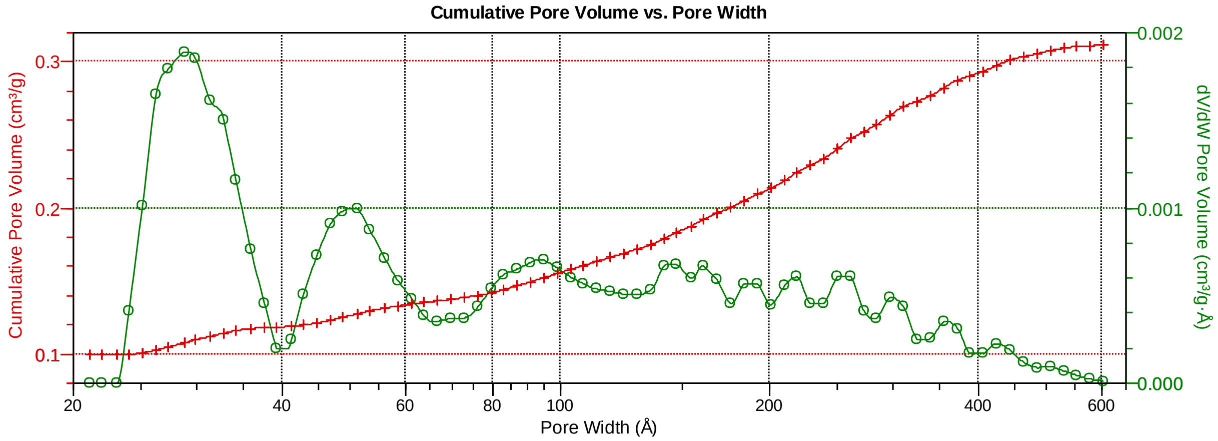

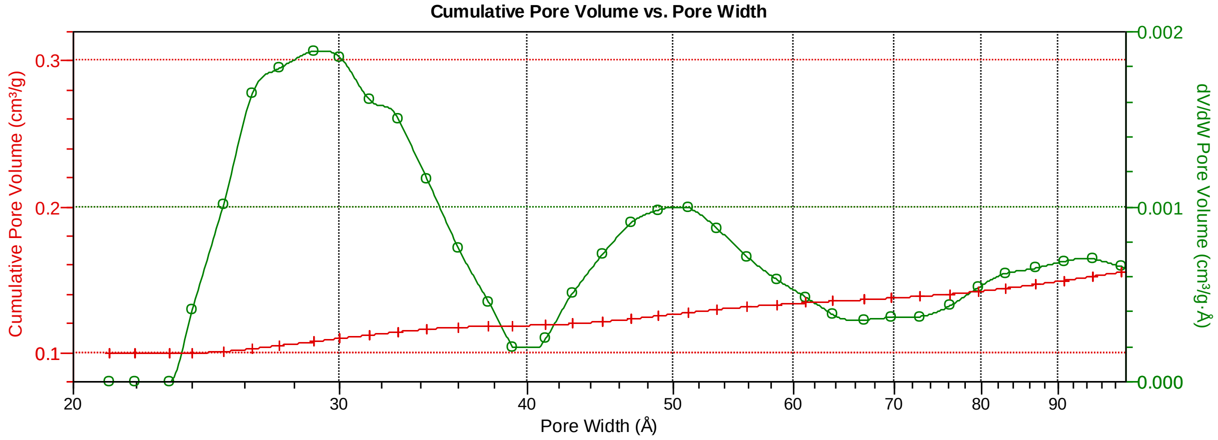

Figure S77. Pore size distribution in **BP1-Pd(BU)** based on N_2@77K sorption measurement (λ = 0.1000).

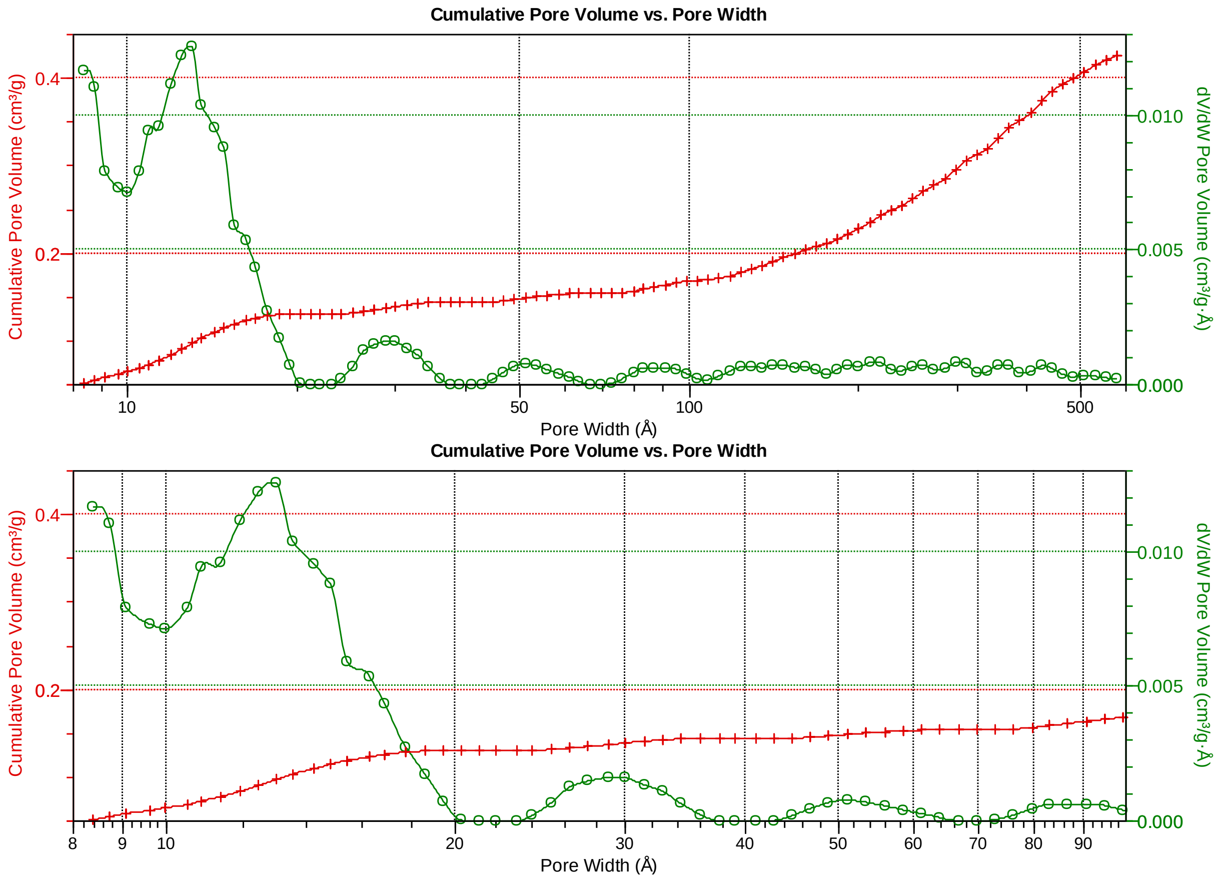

Figure S78. Pore size distribution in **BP2-Pd(BU)** based on N_2@77K sorption measurement (λ = 0.1000).

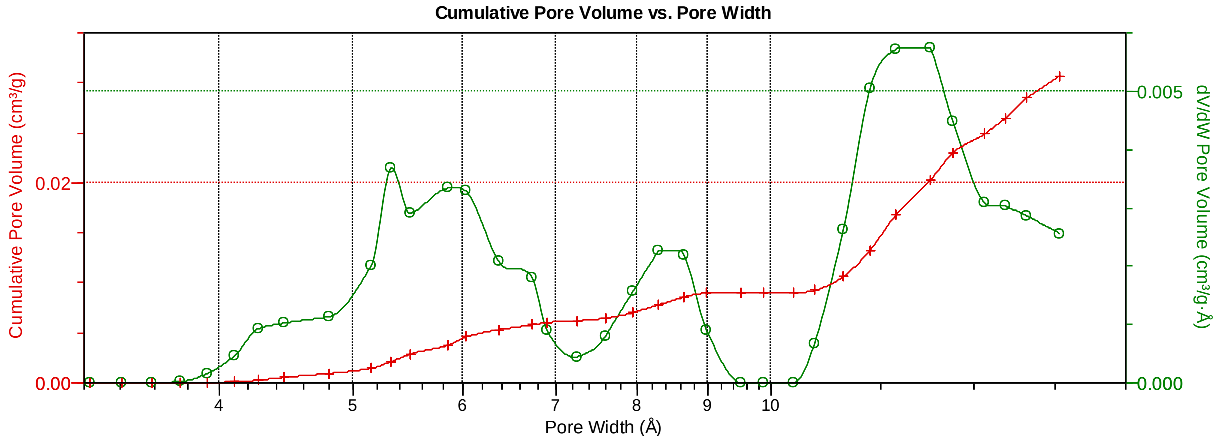

Figure S79. Pore size distribution in **BP2-Pd(BU)** based on CO_2@0°C sorption measurement (λ = 0.0316).

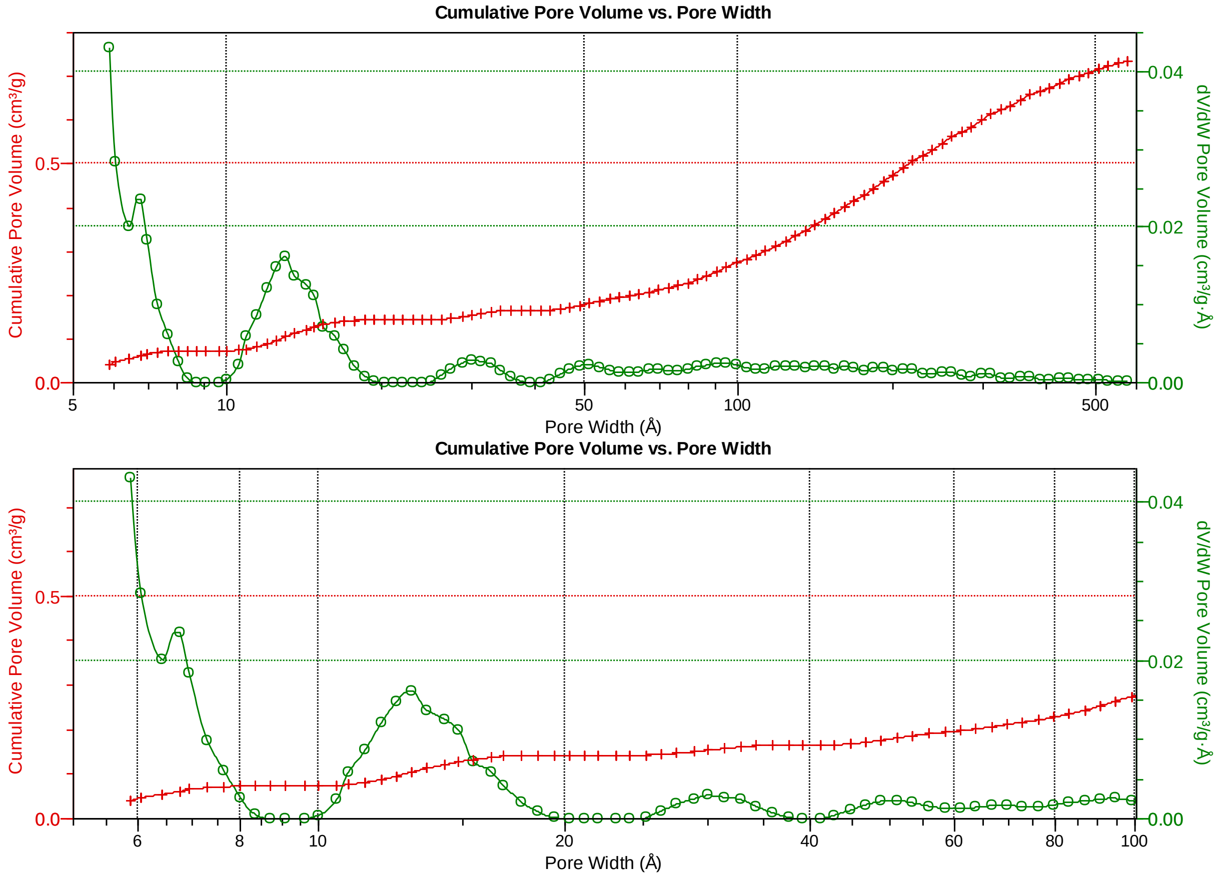

Figure S80. Pore size distribution in **BP1-Pd(PS)** based on N_2@77K sorption measurement (λ = 0.1000).

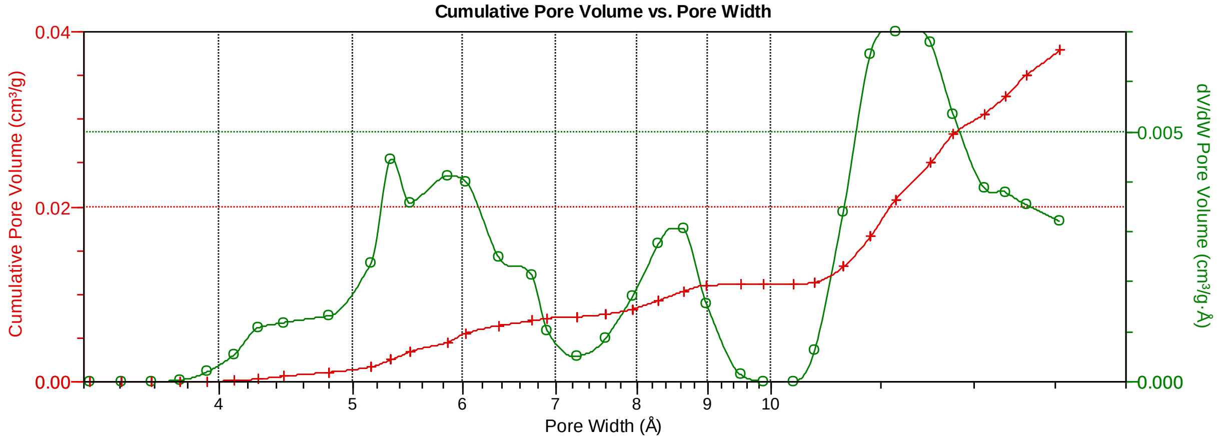

Figure S81. Pore size distribution in **BP1-Pd(PS)** based on CO_2@0°C sorption measurement (λ = 0.0316).

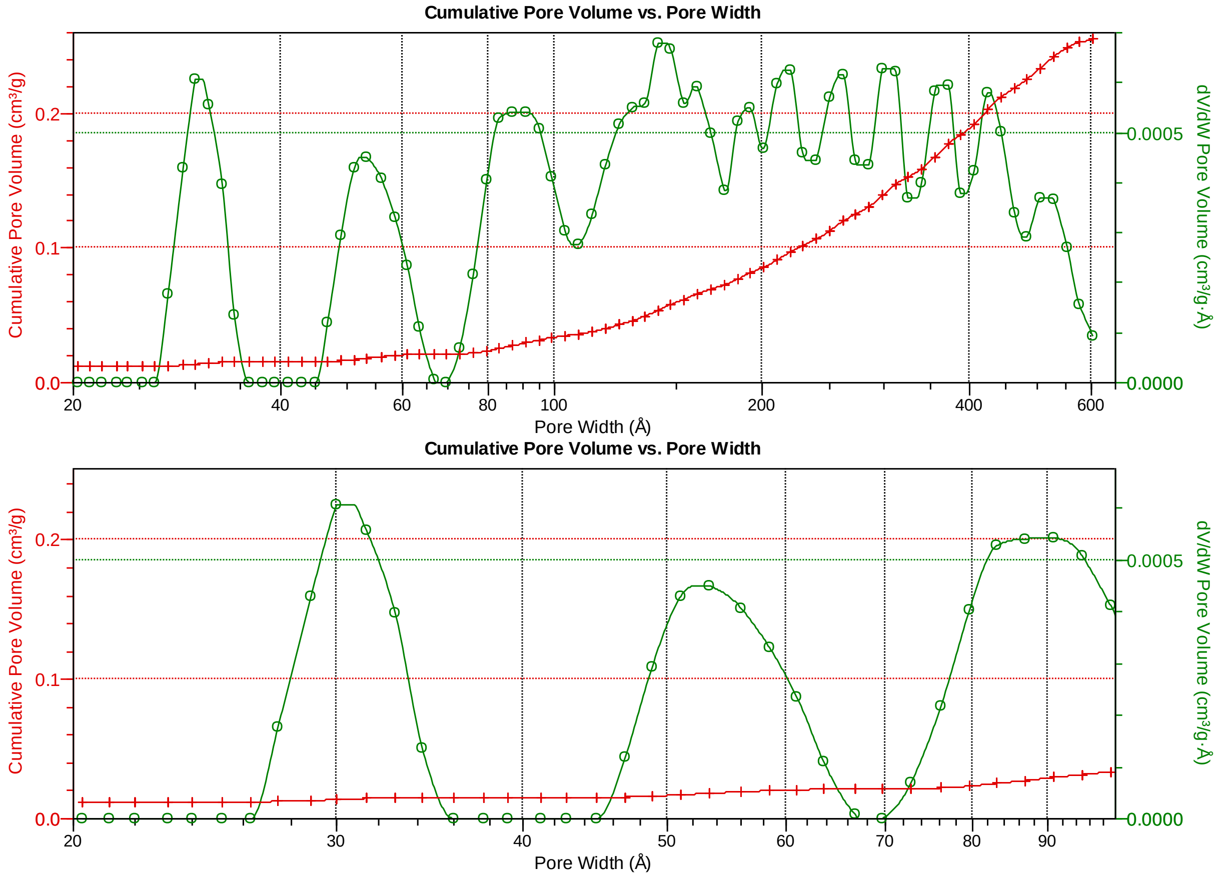

Figure S82. Pore size distribution in **BP2-Pd(PS)** based on N_2@77K sorption measurement (λ = 0.1000).

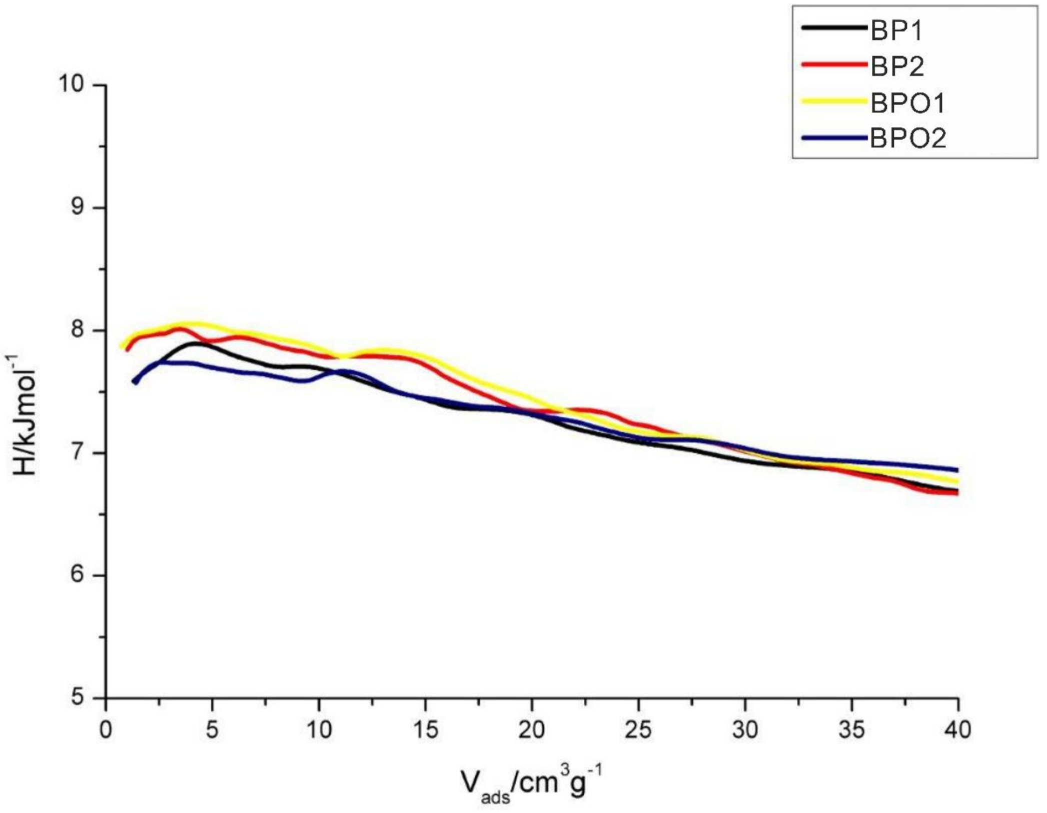

Figure S83. Heat of adsorption for H_2.

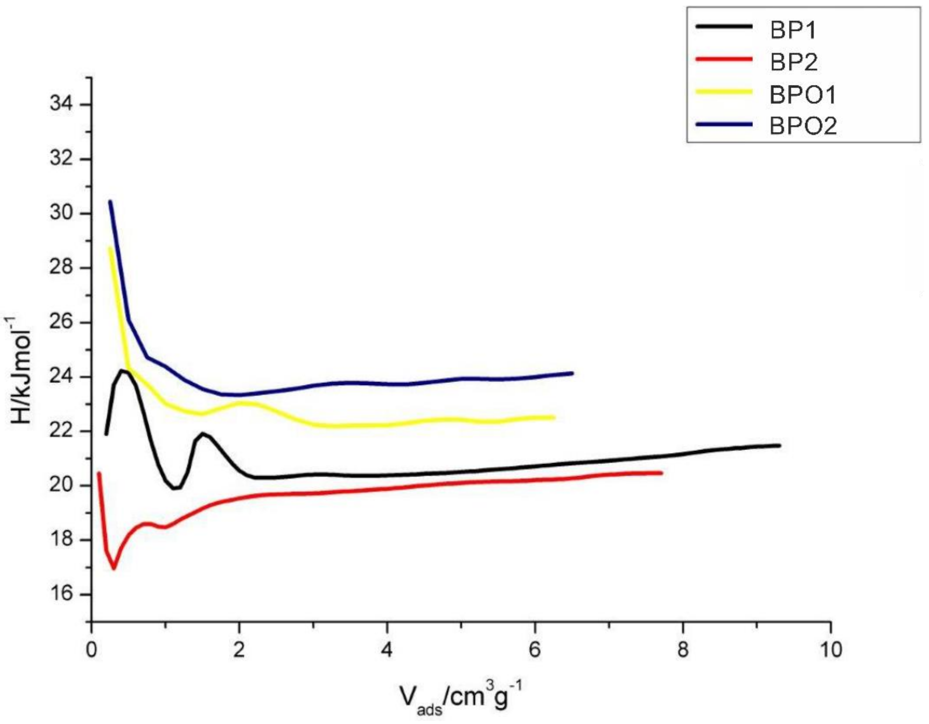

Figure S84. Heat of adsorption for CH_4.

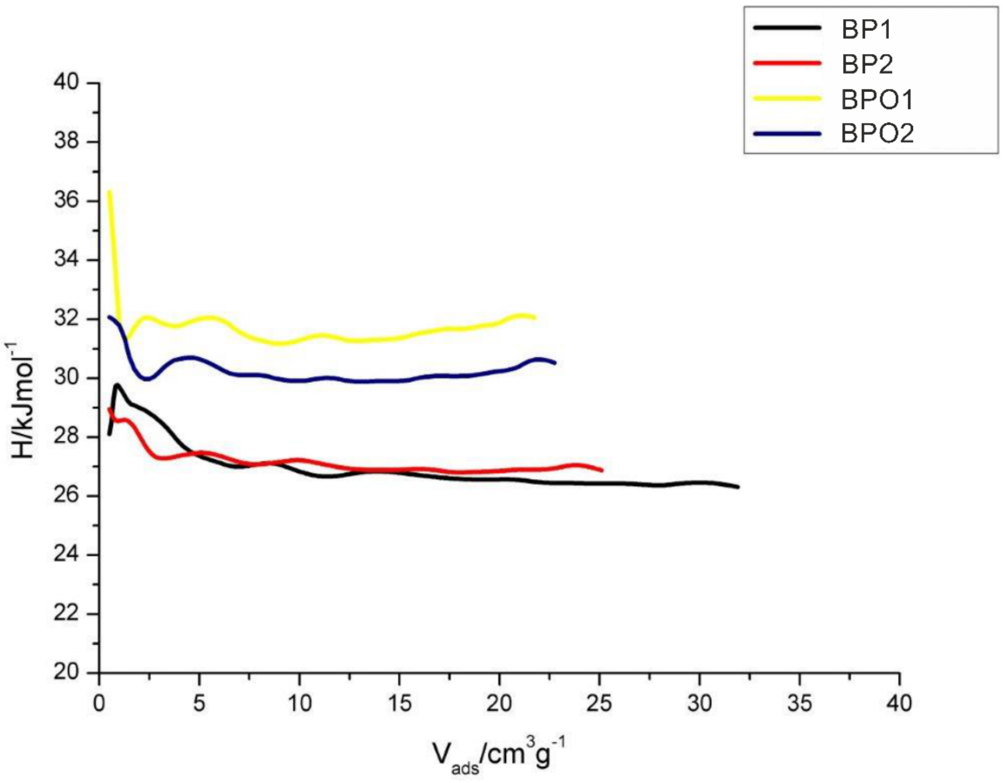

Figure S85. Heat of adsorption for CO_2.

10. Computational studies

The structure **BP1** was initially proposed based on COF-105 structure,[11] where central carbon atom was replaced with phosphorous atom, it was disconnected with one of the four aromatic arm and the symmetry of entire system was reduced to P1. Then interpenetrating network was inserted by centre of symmetry. Such double–catenated network was subjected to initial optimization using semi-empirical PM6 method[12] implemented in *MOPAC* software.[13] Then DFT(B3LYP[14]/TZVP[15]) quantum-chemical optimization including atomic positions and unit cell parameters was performed using *Crystal09* software in order to obtain more precise structural model of **BP1**.[16,17]

In order to calculate the properties of host-guest interactions between framework and tested gases the two molecular fragments were selected comprising unit of HHTP and boronated triphenylphosphine (**BP1'**) or its oxide (**BPO1'**) and then guest molecule (N_2, H_2, CO_2, CH_4) was inserted in-between basic and acidic centres. The obtained adducts were subjected to quantum chemical calculations with M062X[18] method and cc-pVTZ[19] basis set using *Gaussian16* software.[20] Both N_2 adducts were unstable, whereas the remaining systems with except of **BP1**-CO_2 were identified as local minima. The latter system was identified as global minimum. The host-guest interaction energies were calculated using counterpoise approach.

Hirshfeld charges are defined relative to the deformation density - the difference between the molecular and unrelaxed atomic charge densities. The ammounts of charge transfer between donor-guest and acceptor was calculated from the difference between adduct and free molecules. The topology of electron density was analyzed using *AimAll* package[21] in the framework of QTAIM approach.[22]

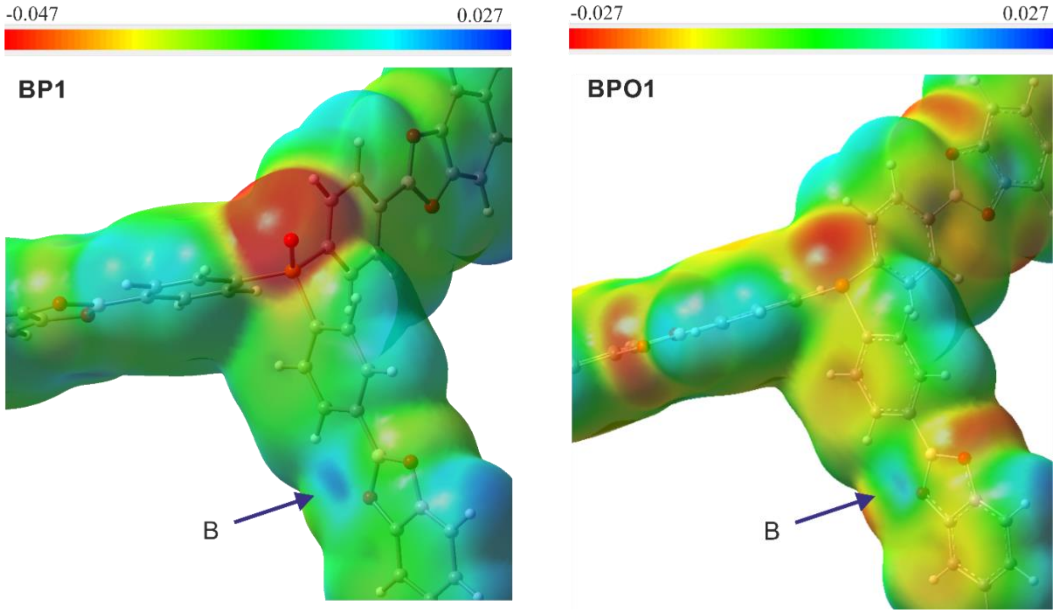

Figure S86. Electrostatic potential map plotted on electron density isosurface (ρ = 0.003 a.u) for **BP1** and **BPO1** showing the strong negative regions around phosphorous/oxygen centres and weak electropositive region above boronate ester plane indicated by blue arrow.

Table S1. Topological analysis of electron density at BCP of guest molecules. ρ and $\nabla^2\rho$ are electron density and its Laplacian at BCP (according to the figure below).

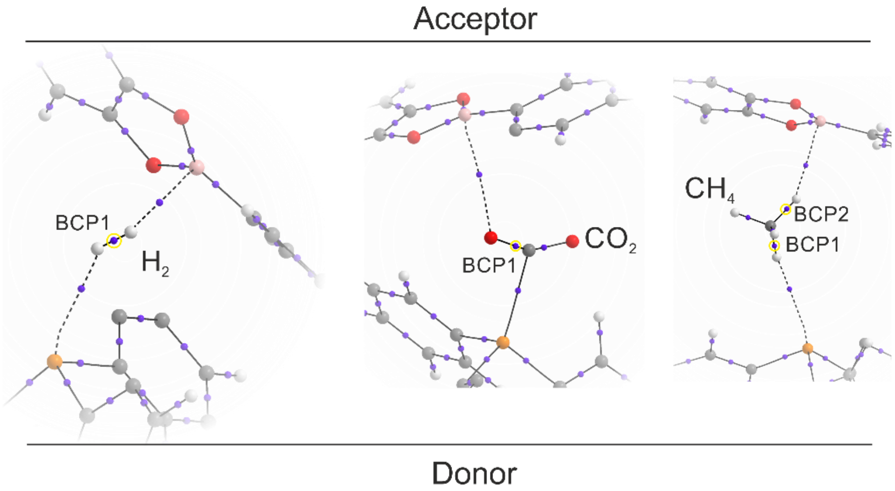

	H_2 (BCP1)		CO_2 (BCP1)		CH_4 (BCP1)		CH_4 (BCP2)	
	ρ / eÅ^{-3}	$\nabla^2\rho$ / eÅ^{-5}	ρ / eÅ^{-3}	$\nabla^2\rho$ / eÅ^{-5}	ρ / eÅ^{-3}	$\nabla^2\rho$ / eÅ^{-5}	ρ / eÅ^{-3}	$\nabla^2\rho$ / eÅ^{-5}
Free	1.842	–30.129	3.158	5.209	1.924	–26.671	1.924	–26.671
**BP1'**	1.808	–29.397	3.038	–6.939	1.876	–23.756	1.903	–24.482
**BPO1'**	1.677	–25.685	3.084	–2.902	1.871	–23.616	1.882	–23.896

Table S2. Hirshfeld atomic charge differences with respect to the free host and guest molecules.

Host	Guest	ΔQ (P/O) / e	ΔQ (B) / e	ΔQ (X) / e	ΔQ (Y) / e
**BP1'**	H_2	+0.030	–0.005	–0.018	–0.009
	CO_2	+0.097	–0.025	–0.115	–0.047
	CH_4	+0.021	–0.023	–0.014	–0.008
**BPO1'**	H_2	+0.038	–0.008	–0.041	–0.005
	CO_2	+0.020	–0.014	–0.013	–0.002
	CH_4	+0.032	–0.020	–0.015	–0.008

Table S3. AIM atomic charge differences with respect to the free host and guest molecules.

Host	Guest	ΔQ (P/O) / e	ΔQ (B) / e	ΔQ (X) / e	ΔQ (Y) / e
**BP1'**	H_2	+0.010	–0.002	–0.048	+0.034
	CO_2	+0.244	–0.058	–0.330	+0.023
	CH_4	+0.064	–0.067	–0.031	+0.005
**BPO1'**	H_2	+0.013	–0.004	–0.113	+0.116
	CO_2	+0.084	–0.047	–0.114	+0.037
	CH_4	+0.080	–0.065	–0.055	+0.012

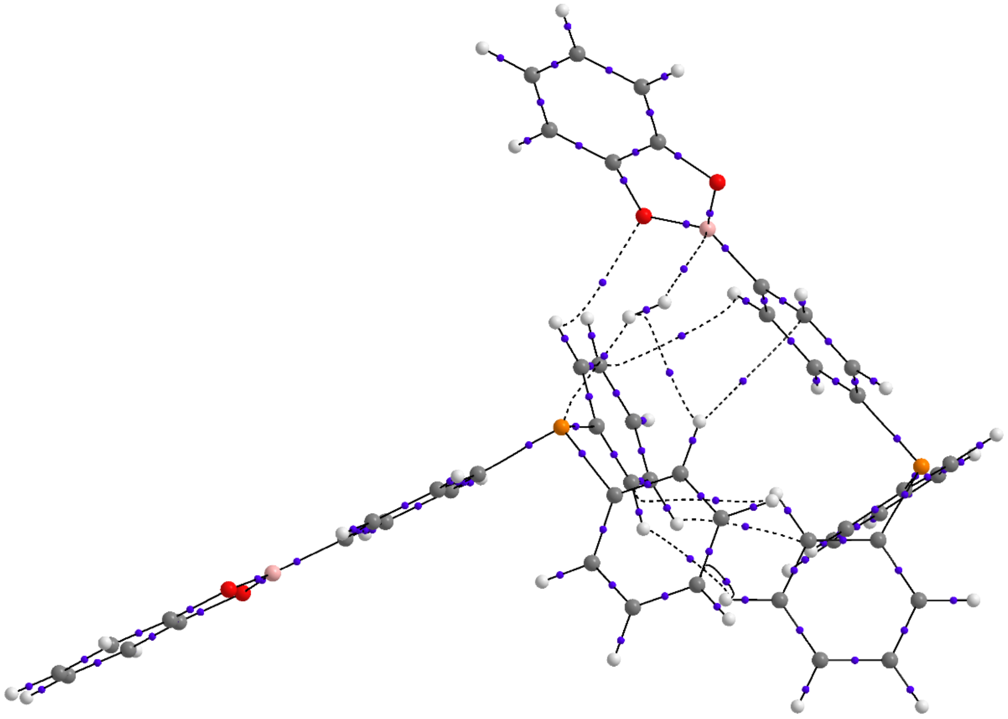

Figure S87. Molecular graph of **BP1'-H$_2$** showing bond paths (black dashed line) and bond critical points (small blue spheres).

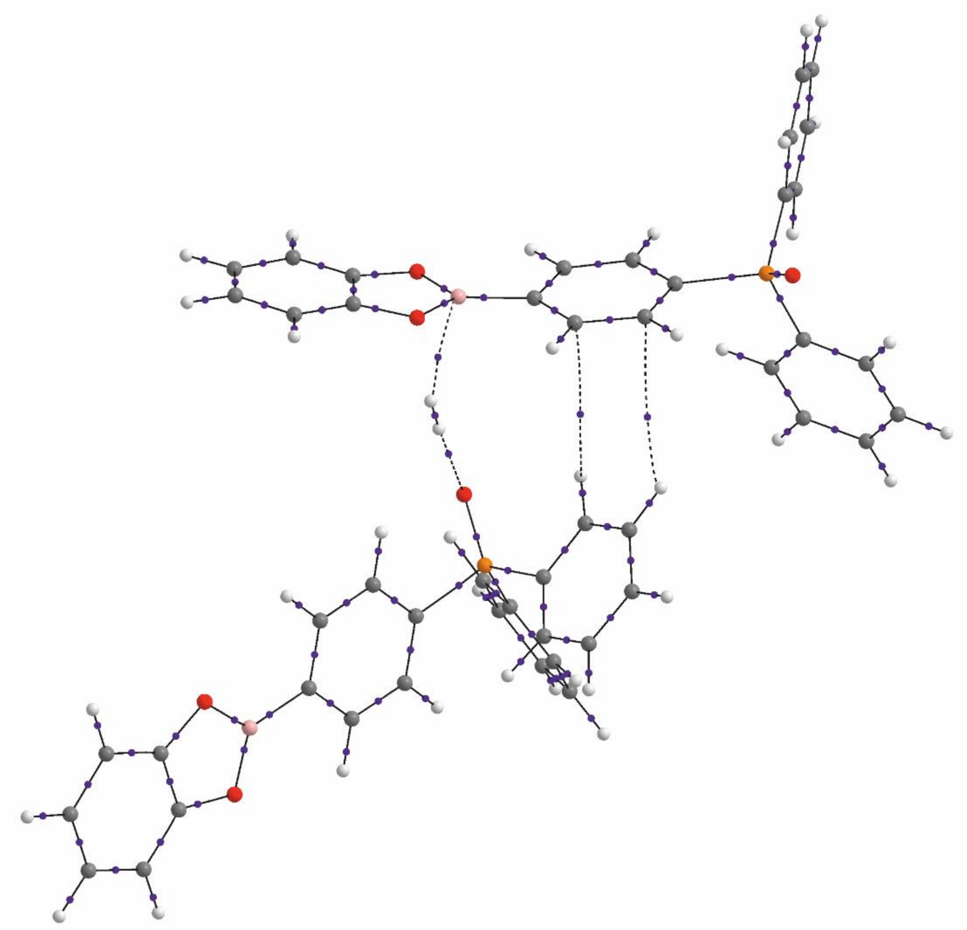

Figure S88. Molecular graph of **BPO1'-H$_2$** showing bond paths (black dashed line) and bond critical points (small blue spheres).

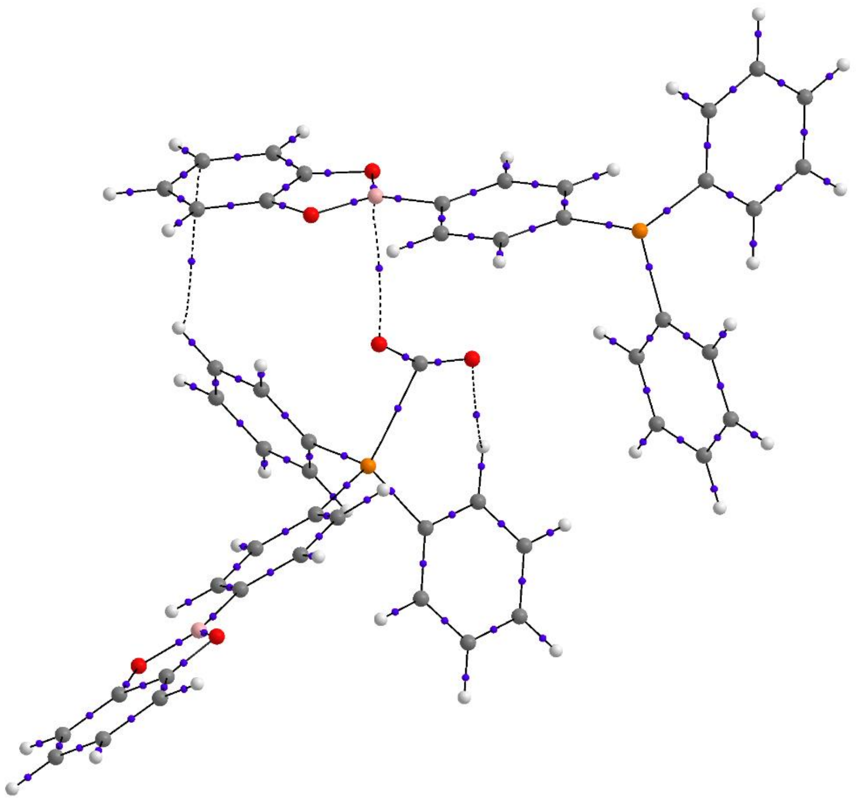

Figure S89. Molecular graph of **BP1'-CO$_2$** showing bond paths (black dashed line) and bond critical points (small blue spheres).

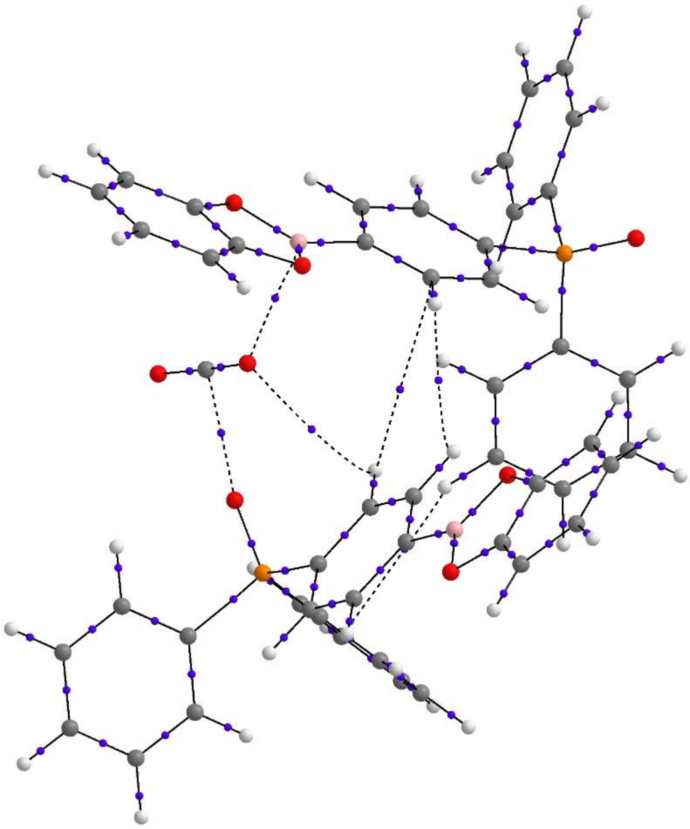

Figure S90. Molecular graph of **BPO1'-CO$_2$** showing bond paths (black dashed line) and bond critical points (small blue spheres).

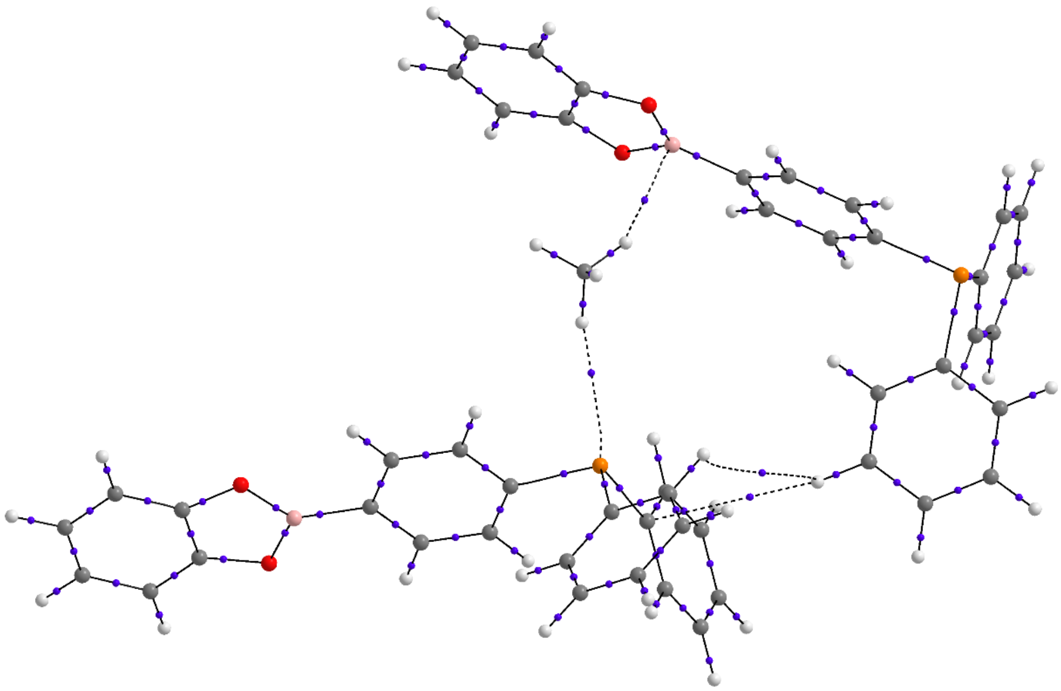

Figure S91. Molecular graph of **BP1'-CH$_4$** showing bond paths (black dashed line) and bond critical points (small blue spheres).

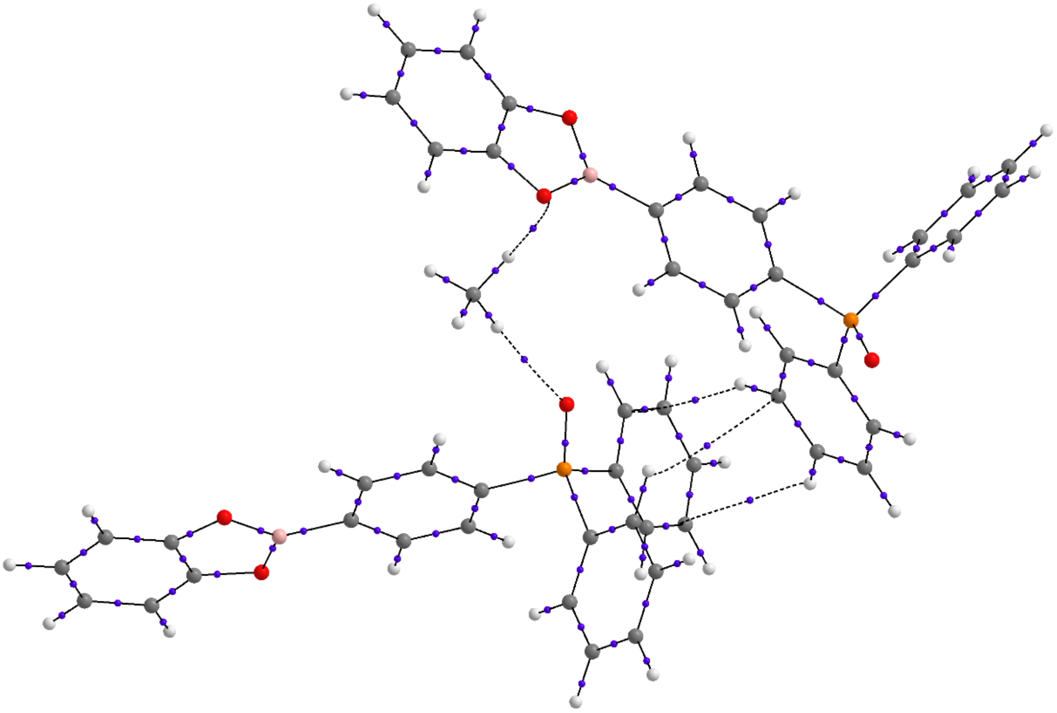

Figure S92. Molecular graph of **BPO1'-CH$_4$** showing bond paths (black dashed line) and bond critical points (small blue spheres).

Table S4. Atomic coordinates, Hirshfeld (Q^H) and AIM (Q^B) atomic charges for **BP1'**-H_2 adduct.

Atom	*x*	*y*	*z*	Q^H / e	Q^B / e	Atom	*x*	*y*	*z*	Q^H / e	Q^B / e
P	5.6885	-1.8397	0.2158	0.1054	1.5951	C	-0.5513	-0.3925	2.2774	-0.0452	1.6141
C	4.3943	-2.8225	1.0804	-0.0491	-0.5754	C	-2.7302	0.3898	0.604	-0.0394	-0.5747
C	3.0601	-2.4442	1.2053	-0.0388	-0.0239	C	-0.1049	0.467	-0.4391	-0.0455	-0.5756
C	4.7914	-4.0628	1.5816	-0.0396	-0.0132	C	-1.3515	-1.4707	2.6462	-0.0385	-0.5817
C	2.1438	-3.2941	1.8136	-0.0400	-0.0200	C	0.6618	-0.2012	2.9463	-0.0328	-0.0165
H	2.7304	-1.4799	0.8385	0.0288	0.0424	C	-3.7269	0.4948	1.5799	-0.0372	-0.0179
C	3.8746	-4.9155	2.1754	-0.0407	-0.0121	C	-3.0888	-0.0178	-0.6788	-0.0406	-0.0159
H	5.831	-4.3589	1.5071	0.0396	0.0189	C	0.3402	-0.8188	-0.7298	-0.0307	-0.0078
C	2.5465	-4.5306	2.2942	-0.0399	-0.0160	C	0.0445	1.4647	-1.4014	-0.0298	-0.0106
H	1.1138	-2.9821	1.9315	0.0288	0.0332	C	-0.9692	-2.3064	3.6904	-0.0372	-0.0098
H	4.198	-5.8747	2.5564	0.0433	0.0157	H	-2.281	-1.66	2.1262	0.0359	-0.0193
C	4.8464	-0.2136	0.0357	-0.0395	-0.5745	C	1.0477	-1.0458	3.9736	-0.0323	0.0381
C	4.6721	0.5482	1.194	-0.0368	-0.0134	H	1.311	0.6162	2.6551	0.0385	-0.0040
C	4.377	0.2935	-1.1733	-0.0383	-0.0067	C	-5.0396	0.1766	1.287	-0.0268	0.0301
C	4.0263	1.7706	1.1445	-0.0269	-0.0203	H	-3.4653	0.8293	2.5771	0.0422	-0.0155
H	5.0436	0.1733	2.141	0.0416	0.0196	C	-4.4101	-0.326	-0.9725	-0.0256	0.0209
C	3.7371	1.5252	-1.2204	-0.0248	-0.0090	H	-2.3379	-0.1011	-1.4527	0.0342	-0.0158
H	4.4989	-0.2753	-2.0855	0.0331	0.0363	C	0.9107	-1.1023	-1.9626	-0.0359	0.0390
C	3.5551	2.2879	-0.068	-0.0737	-0.6799	H	0.2255	-1.6063	0.0024	0.0355	-0.0107
H	3.8952	2.3447	2.0541	0.0433	0.0259	C	0.6026	1.1755	-2.6385	-0.0336	0.0307
H	3.3749	1.9003	-2.169	0.0391	0.0250	H	-0.2753	2.4753	-1.1788	0.0378	-0.0110
C	5.5551	-2.5194	-1.4878	-0.0475	-0.5687	C	0.2249	-2.0915	4.3601	-0.0343	0.0370
C	4.3784	-3.0428	-2.0213	-0.0334	-0.0117	H	-1.6105	-3.1298	3.9755	0.0441	-0.0120
C	6.6804	-2.4269	-2.3035	-0.0354	-0.0132	H	1.9937	-0.8887	4.4732	0.0464	0.0179
C	4.3249	-3.4474	-3.3465	-0.0396	-0.0160	C	-5.404	-0.2407	0.0011	-0.0786	0.0237
H	3.5069	-3.149	-1.3882	0.0350	0.0361	H	-5.798	0.2573	2.0556	0.0451	-0.6891
C	6.6266	-2.8245	-3.6334	-0.0395	-0.0123	H	-4.6744	-0.6422	-1.974	0.0448	0.0317
H	7.6045	-2.0394	-1.8923	0.0408	0.0243	C	1.0349	-0.1127	-2.9194	-0.0335	0.0310
C	5.4475	-3.3314	-4.1572	-0.0369	-0.0139	H	1.2538	-2.1058	-2.1756	0.0416	-0.0124
H	3.4078	-3.8589	-3.7473	0.0430	0.0150	H	0.7152	1.9624	-3.3724	0.0450	0.0177
H	5.4044	-3.6468	-5.1909	0.0444	0.0177	H	0.521	-2.7431	5.1706	0.0452	0.0221
C	2.1688	5.6483	0.5474	0.0607	0.4884	B	-6.8681	-0.5825	-0.3334	0.2322	0.0195
C	1.4287	5.2849	-0.5684	0.0600	0.4767	H	1.48	-0.3411	-3.879	0.0445	2.2338
C	2.013	6.874	1.1519	-0.0459	0.0332	O	-7.3082	-1.0324	-1.5692	-0.1673	0.0186
C	0.4764	6.1112	-1.1168	-0.0479	0.0314	O	-7.9236	-0.485	0.561	-0.1676	-1.3735
C	1.048	7.7212	0.6078	-0.0466	0.0016	C	-8.6602	-1.2147	-1.4331	0.0599	-1.3735
H	2.6081	7.1551	2.0083	0.0542	0.0451	C	-9.0346	-0.8816	-0.1379	0.0599	0.4813
C	0.2941	7.3472	-0.4983	-0.0469	0.0023	C	-9.5721	-1.6481	-2.3666	-0.0474	0.4811
H	-0.0921	5.8148	-1.9862	0.0528	0.0416	C	-10.3394	-0.9656	0.2877	-0.0474	0.0325
H	0.8856	8.6911	1.0562	0.0437	0.0206	C	-10.8989	-1.738	-1.9465	-0.0475	0.0326
H	-0.4456	8.0298	-0.8918	0.0438	0.0207	H	-9.2679	-1.9032	-3.3711	0.0538	0.0008
O	1.8322	4.0422	-0.9841	-0.1583	-1.3810	C	-11.2737	-1.4048	-0.6501	-0.0475	0.0446
O	3.0486	4.643	0.8496	-0.1660	-1.3738	H	-10.6173	-0.7034	1.298	0.0539	0.0007
B	2.8107	3.6429	-0.0875	0.2280	2.2332	H	-11.651	-2.0745	-2.646	0.0434	0.0447
H	1.8300	-5.1867	2.7701	0.0421	0.0150	H	-12.3117	-1.4875	-0.3605	0.0435	0.0202
H	7.5074	-2.745	-4.2559	0.0446	0.0188	H	1.4013	2.7947	1.4808	-0.0181	0.0203
P	-1.0317	0.9216	1.0758	0.1380	1.5951	H	0.6563	2.7136	1.5257	-0.0093	-0.0483

Table S5. Atomic coordinates, Hirshfeld (Q^H) and AIM (Q^B) atomic charges for **BPO1'**-H_2 adduct.

Atom	*x*	*y*	*z*	Q^H / e	Q^B / e	Atom	*x*	*y*	*z*	Q^H / e	Q^B / e
P	-6.2856	0.0008	-1.3475	0.3745	3.2012	C	3.2445	-1.2025	0.5675	-0.0448	-0.6599
C	-6.0525	-1.7558	-0.9406	-0.0520	-0.6743	C	1.5157	-3.0144	2.0087	-0.0488	-0.6795
C	-5.3379	-2.207	0.1665	-0.0331	-0.2675	C	1.7917	-2.4156	-1.7029	-0.0320	-0.0158
C	-6.6117	-2.6758	-1.8243	-0.0227	0.0016	C	-0.3499	-1.4776	-1.0857	-0.0234	0.0034
C	-5.2082	-3.5699	0.4067	-0.0375	-0.0050	C	3.5499	0.0903	0.1456	-0.0226	-0.0020
H	-4.8865	-1.4958	0.8485	0.0394	0.0254	C	4.2535	-2.1551	0.6738	-0.0368	-0.0220
C	-6.4763	-4.0362	-1.5923	-0.0338	-0.0076	C	1.602	-4.3129	1.5146	-0.0317	-0.0158
H	-7.1391	-2.3111	-2.6964	0.0507	0.0670	C	1.4425	-2.7935	3.3811	-0.0202	0.0043
C	-5.7818	-4.4842	-0.4644	-0.0309	-0.0119	C	1.3469	-2.6381	-2.9963	-0.0377	-0.0006
H	-4.6451	-3.9155	1.2628	0.0469	0.0241	H	2.8235	-2.629	-1.4525	0.0359	0.0378
H	-6.91	-4.7458	-2.2836	0.0474	0.0249	C	-0.794	-1.6661	-2.3826	-0.0301	-0.0110
C	-4.8103	0.927	-0.7983	-0.0434	-0.6594	H	-0.962	-0.9636	-0.3543	0.0502	0.0640
C	-3.8207	1.2326	-1.7432	-0.0178	-0.0135	C	4.8552	0.4191	-0.1773	-0.0201	-0.0090
C	-4.6111	1.3647	0.4993	-0.0366	-0.0155	H	2.7634	0.8323	0.0901	0.0506	0.0590
C	-2.7067	1.9989	-1.3882	-0.0178	0.0176	C	5.5585	-1.8188	0.3466	-0.0259	-0.0154
H	-3.9612	0.9174	-2.7692	0.0484	0.0630	H	4.0248	-3.1542	1.0221	0.0355	0.0286
C	-3.4852	2.1102	0.7948	-0.0178	-0.0259	C	1.6312	-5.388	2.3911	-0.0374	-0.0059
H	-5.3533	1.1639	1.2619	0.0388	0.0231	H	1.6342	-4.4868	0.4461	0.0405	0.0287
C	-2.5407	2.4647	-0.175	-0.0716	-0.6813	C	1.4699	-3.8684	4.2567	-0.0326	-0.0049
H	-1.9555	2.2324	-2.132	0.0499	0.0334	H	1.3482	-1.7789	3.746	0.0525	0.0699
H	-3.3287	2.4573	1.8081	0.0438	0.0345	C	0.0565	-2.2821	-3.3473	-0.0257	-0.0163
C	-7.6926	0.5655	-0.3489	-0.0523	-0.6776	H	2.0172	-3.0609	-3.7321	0.0476	0.0238
C	-8.0402	0.0028	0.8752	-0.0335	-0.0153	H	-1.7812	-1.3346	-2.6599	0.0460	0.0337
C	-8.4331	1.6275	-0.8618	-0.0211	0.0018	C	5.8761	-0.531	-0.086	-0.0718	-0.6797
C	-9.1137	0.5116	1.5925	-0.0376	-0.0055	H	5.0935	1.4241	-0.5012	0.0485	0.0382
H	-7.4837	-0.8405	1.2648	0.0373	0.0288	H	6.3441	-2.5586	0.4332	0.0452	0.0328
C	-9.5068	2.1328	-0.1452	-0.0329	-0.0044	C	1.5677	-5.1679	3.7601	-0.0297	-0.0113
H	-8.1681	2.0349	-1.8291	0.0520	0.0644	H	1.6956	-6.3965	2.006	0.0465	0.0228
C	-9.8447	1.5764	1.0853	-0.0298	-0.0104	H	1.4101	-3.6965	5.3226	0.0485	0.0267
H	-9.384	0.0715	2.5426	0.0459	0.0214	H	-0.2953	-2.448	-4.3563	0.0487	0.0292
H	-10.6834	1.9692	1.6441	0.0469	0.0222	B	7.3325	-0.1613	-0.4423	0.2342	2.2340
C	0.2674	4.8072	0.2492	0.0557	0.5207	H	1.5871	-6.007	4.4424	0.0473	0.0234
C	-0.2313	4.7144	1.5584	0.0590	0.4302	O	8.4032	-1.0363	-0.3645	-0.1672	-1.3736
C	1.3433	5.6104	-0.0377	-0.0515	0.0207	O	7.7404	1.0887	-0.875	-0.1656	-1.3725
C	0.3341	5.4325	2.5844	-0.0450	0.0258	C	9.4986	-0.3085	-0.7568	0.0601	0.4773
C	1.8795	6.3158	1.0399	-0.0518	0.0179	C	9.0945	0.9834	-1.0703	0.0605	0.4790
H	1.7371	5.6841	-1.0408	0.0517	0.0416	C	10.8096	-0.7089	-0.8603	-0.0471	0.0339
C	1.4188	6.2444	2.2474	-0.0564	0.0279	C	9.9822	1.9411	-1.4998	-0.0461	0.0340
H	-0.045	5.3614	3.5932	0.0521	0.0405	C	11.721	0.2522	-1.2962	-0.0468	0.0020
H	2.7271	6.9657	0.8747	0.0404	-0.0002	H	11.1091	-1.7175	-0.6163	0.0541	0.0451
H	1.8979	6.8276	3.0211	0.0417	0.0043	C	11.3187	1.5451	-1.6098	-0.0464	0.0002
O	-1.2827	3.8101	1.6518	-0.1521	-1.3775	H	9.6554	2.943	-1.7362	0.0549	0.0472
O	-0.4388	4.0339	-0.6252	-0.1901	-1.3196	H	12.7637	-0.0159	-1.3913	0.0440	0.0213
B	-1.4503	3.3623	0.3595	0.2554	2.2251	H	12.0545	2.2627	-1.944	0.0440	0.0218
H	-5.6752	-5.5444	-0.2788	0.0464	0.0219	O	0.8369	-0.3994	1.6075	-0.4163	-1.5443
H	-10.0831	2.9554	-0.546	0.0479	0.0251	H	0.2901	2.0487	0.4706	-0.3747	-1.5467
O	-6.4905	0.2093	-2.8087	0.3821	3.2248	H	0.4805	1.3565	0.7907	-0.0408	-0.1136
P	1.4995	-1.5478	0.9412	0.3745	-0.6957	C	1.5157	-3.0144	2.0087	-0.0048	0.1157
C	0.9392	-1.8686	-0.746	-0.0520	3.2012	C	1.7917	-2.4156	-1.7029	-0.0448	-0.6599

Table S6. Atomic coordinates, Hirshfeld (Q^H) and AIM (Q^B) atomic charges for **BP1'**-CO_2 adduct.

Atom	*x*	*y*	*z*	Q^H / e	Q^B / e	Atom	*x*	*y*	*z*	Q^H / e	Q^B / e
P	-2.5814	1.7833	-0.9193	0.1040	1.4489	C	-0.7153	-6.8688	3.3124	0.0438	-0.5429
C	-2.0655	1.7416	0.8415	-0.0480	-0.5262	C	1.5487	-7.8062	1.7836	0.2048	-0.5493
C	-1.3217	0.7149	1.4192	-0.0358	-0.1211	C	1.6757	-6.0209	5.1738	-0.0481	-0.0122
C	-2.4758	2.8088	1.6425	-0.0372	-0.0155	C	3.1065	-5.1478	3.4424	-0.0384	-0.0110
C	-0.9874	0.7615	2.7662	-0.0391	-0.0180	C	-1.7442	-5.9452	3.4972	-0.0487	-0.0039
H	-1.0004	-0.1271	0.8188	0.0382	0.0264	C	-0.8874	-8.1777	3.764	-0.0356	-0.0119
C	-2.1288	2.8629	2.984	-0.0409	-0.0182	C	2.6975	-8.4101	2.2907	-0.0284	-0.0142
H	-3.0761	3.6012	1.2093	0.0407	0.0253	C	0.9338	-8.3508	0.6537	-0.0274	-0.0129
C	-1.3842	1.8362	3.5495	-0.0393	-0.0191	C	2.5814	-5.5911	6.1336	-0.0312	-0.0141
H	-0.4113	-0.0457	3.2015	0.0449	0.0234	H	0.7669	-6.5268	5.4749	-0.0366	0.0384
H	-2.4491	3.7016	3.5901	0.0435	0.0205	C	4.0146	-4.7333	4.4045	-0.0309	-0.0133
C	-2.2282	0.0621	-1.4495	-0.0415	-0.5231	H	3.2938	-4.9408	2.3962	-0.0373	0.0579
C	-3.223	-0.8849	-1.195	-0.0384	-0.0167	C	-2.9111	-6.3164	4.1433	0.0375	-0.0093
C	-1.0523	-0.3491	-2.0745	-0.0379	-0.0171	H	-1.6346	-4.9356	3.1197	-0.0345	0.0410
C	-3.035	-2.2133	-1.5304	-0.0249	-0.0124	C	-2.0597	-8.5464	4.4027	0.0451	-0.0122
H	-4.1513	-0.5715	-0.7301	0.0405	0.0240	H	-0.1045	-8.9092	3.6095	-0.0208	0.0411
C	-0.8721	-1.6785	-2.4239	-0.0232	-0.0118	C	3.215	-9.5467	1.6848	0.0466	-0.0141
H	-0.2747	0.373	-2.2901	0.0364	0.0375	H	3.1846	-7.9948	3.1634	-0.0235	0.0396
C	-1.8559	-2.6335	-2.1545	-0.0736	-0.6787	C	1.448	-9.4916	0.0586	0.0411	-0.0105
H	-3.8123	-2.9371	-1.3168	0.0453	0.0354	H	0.0506	-7.8808	0.2357	-0.0375	0.0514
H	0.0526	-1.9837	-2.8995	0.0460	0.0372	C	3.7531	-4.9533	5.7509	0.0370	-0.0150
C	-1.2233	2.7267	-1.7172	-0.0482	-0.5231	H	2.372	-5.7595	7.1828	-0.0340	0.0259
C	-0.0479	3.1072	-1.074	-0.0381	-0.0159	H	4.9223	-4.2261	4.1022	0.0449	0.0285
C	-1.4144	3.1082	-3.0468	-0.0370	-0.0153	C	-3.0896	-7.6231	4.6059	-0.0335	-0.6750
C	0.9174	3.8447	-1.7477	-0.0399	-0.0174	H	-3.702	-5.5886	4.2793	0.0463	0.0432
H	0.1169	2.8267	-0.0414	0.0358	0.0377	H	-2.1818	-9.5658	4.7494	0.0480	0.0399
C	-0.4448	3.831	-3.7237	-0.0401	-0.0170	C	2.5903	-10.0914	0.5722	-0.0693	-0.0151
H	-2.3337	2.8368	-3.5543	0.0412	0.0251	H	4.1077	-10.0091	2.088	0.0492	0.0249
C	0.7247	4.2039	-3.0734	-0.0395	-0.0188	H	0.956	-9.8998	-0.8148	0.0472	0.0318
H	1.8257	4.1341	-1.2332	0.0435	0.0205	H	4.4596	-4.6213	6.5016	-0.0338	0.0256
H	1.4793	4.7772	-3.5976	0.0433	0.0200	B	-4.3951	-8.0397	5.3107	0.0459	2.1703
C	-2.0113	-6.2477	-2.8065	0.0625	0.5044	H	2.997	-10.9781	0.1016	0.0459	0.0248
C	-0.7332	-5.9547	-3.2563	0.0616	0.5015	O	-4.6508	-9.312	5.7971	0.0465	-1.3191
C	-2.5299	-7.5209	-2.8587	-0.0479	-0.0029	O	-5.4667	-7.1923	5.5411	0.2351	-1.3185
C	0.0883	-6.9157	-3.7981	-0.0478	-0.0017	C	-5.9065	-9.2459	6.3382	0.0462	0.4889
C	-1.7072	-8.5063	-3.4037	-0.0513	-0.0165	C	-6.402	-7.9588	6.1824	-0.1658	0.4895
H	-3.5263	-7.7397	-2.4994	0.0533	0.0439	C	-6.6341	-10.2385	6.9532	-0.1648	0.0005
C	-0.4297	-8.209	-3.8678	-0.0509	-0.0166	C	-7.6507	-7.598	6.6333	0.0596	0.0010
H	1.0808	-6.6745	-4.1534	0.0536	0.0448	C	-7.9012	-9.8852	7.4148	0.0600	-0.0100
H	-2.0753	-9.522	-3.4723	0.0408	0.0190	H	-6.2393	-11.2386	7.0697	-0.0468	0.0467
H	0.1738	-8.9963	-4.302	0.0406	0.0183	C	-8.398	-8.5952	7.2584	-0.0461	-0.0097
O	-0.4949	-4.6214	-3.0813	-0.1648	-1.3181	H	-8.0281	-6.5925	6.5065	-0.0465	0.0481
O	-2.5962	-5.1013	-2.353	-0.1638	-1.3186	H	-8.511	-10.6328	7.9054	0.0545	0.0255
B	-1.6573	-4.1035	-2.5382	0.2268	2.1809	H	-9.3868	-8.3586	7.6294	-0.0462	0.0259
H	-1.1195	1.8703	4.5992	0.1040	0.0197	O	-0.5388	-5.0406	0.2595	0.0552	-1.1637
H	-0.6063	4.113	-4.757	-0.0480	0.0211	C	0.4963	-4.718	0.7193	0.0443	2.0443
P	0.8069	-6.2978	2.4894	-0.0358	1.6186	O	1.461	-4.0395	0.7636	0.0445	-1.1530
C	1.9371	-5.8102	3.8219	0.0432	-0.5635						

Table S7. Atomic coordinates, Hirshfeld (Q^H) and AIM (Q^B) atomic charges for **BPO1'**-CO_2 adduct.

Atom	*x*	*y*	*z*	Q^H / e	Q^B / e	Atom	*x*	*y*	*z*	Q^H / e	Q^B / e
P	-2.472	0.6981	0.5187	0.3703	2.9245	C	-1.0326	-6.6374	3.7553	-0.0519	-0.5987
C	-1.5075	0.5772	2.0471	-0.0528	-0.5918	C	1.6121	-7.8451	4.0199	-0.0450	-0.5963
C	-0.4636	-0.3307	2.2139	-0.0323	-0.0204	C	0.5119	-4.7821	5.8697	-0.0523	-0.6022
C	-1.8519	1.4357	3.0881	-0.0224	-0.0057	C	2.3514	-4.3016	4.3859	-0.0303	-0.0174
C	0.239	-0.3728	3.4105	-0.0385	-0.0153	C	-1.9069	-5.707	3.1946	-0.0205	-0.0057
H	-0.2097	-1.0187	1.4161	0.0399	0.0345	C	-1.5378	-7.75	4.4222	-0.0186	0.0019
C	-1.1478	1.3917	4.2815	-0.0361	-0.0136	C	2.005	-8.091	5.334	-0.0333	-0.0172
H	-2.6879	2.111	2.9546	0.0510	0.0652	C	1.8946	-8.7794	3.0266	-0.0319	-0.0173
C	-0.1019	0.4914	4.4413	-0.0331	-0.0147	C	0.9432	-3.7812	6.7308	-0.0197	-0.0024
H	1.0407	-1.0891	3.5421	0.0405	0.0306	H	-0.3851	-5.3439	6.1022	-0.0350	-0.0129
H	-1.4195	2.0561	5.0924	0.0458	0.0244	C	2.7787	-3.304	5.2481	0.0408	0.0357
C	-2.4127	-0.95	-0.224	-0.0470	-0.5919	H	2.8753	-4.5004	3.4596	-0.0328	-0.0153
C	-3.2276	-1.9313	0.3426	-0.0233	-0.0079	C	-3.2732	-5.892	3.3075	0.0525	0.0643
C	-1.5897	-1.2735	-1.2978	-0.0337	-0.0189	H	-1.5113	-4.8606	2.6472	-0.0178	-0.0055
C	-3.1917	-3.2246	-0.1459	-0.0225	-0.0103	C	-2.9091	-7.9279	4.5341	0.0493	0.0609
H	-3.8892	-1.6684	1.159	0.0507	0.0504	H	-0.8614	-8.4836	4.8436	-0.0244	-0.0117
C	-1.5591	-2.5734	-1.7839	-0.0269	-0.0147	C	2.6707	-9.2667	5.6536	0.0383	0.0373
H	-0.9642	-0.5133	-1.75	0.0377	0.0362	H	1.8067	-7.3582	6.107	-0.0365	-0.0138
C	-2.3498	-3.5677	-1.2092	-0.0673	-0.6529	C	2.561	-9.9514	3.3491	0.0387	0.0334
H	-3.8199	-3.9859	0.3008	0.0456	0.0358	H	1.6098	-8.5692	2.004	-0.0322	-0.0104
H	-0.9113	-2.8196	-2.6167	0.0456	0.0370	C	2.0781	-3.0469	6.4204	0.0520	0.0621
C	-1.5788	1.8075	-0.5975	-0.0514	-0.5932	H	0.3903	-3.5708	7.6378	-0.0289	-0.0143
C	-0.2204	2.0852	-0.4757	-0.0331	-0.0171	H	3.6613	-2.7248	5.0064	0.0481	0.0287
C	-2.3203	2.4128	-1.6108	-0.0212	-0.0054	C	-3.7948	-7.0012	3.9822	0.0480	0.0278
C	0.3961	2.954	-1.3665	-0.0384	-0.0152	H	-3.9492	-5.1719	2.8627	-0.0666	-0.6701
H	0.3563	1.6323	0.3219	0.0378	0.0342	H	-3.2982	-8.7979	5.0493	0.0483	0.0417
C	-1.7024	3.2802	-2.4977	-0.0347	-0.0126	C	2.9455	-10.1964	4.6612	0.0470	0.0395
H	-3.3837	2.218	-1.6754	0.0519	0.0617	H	2.9827	-9.4522	6.6739	-0.0287	-0.0122
C	-0.3441	3.5474	-2.3785	-0.0318	-0.0143	H	2.7843	-10.6737	2.5741	0.0471	0.0271
H	1.4525	3.1703	-1.2667	0.0455	0.0240	H	2.4149	-2.2653	7.0906	0.0491	0.0303
H	0.1366	4.2251	-3.0735	0.0461	0.0242	B	-5.3207	-7.2018	4.1037	0.0473	0.0258
C	-2.9451	-7.0584	-2.1671	0.0609	0.5038	H	3.4672	-11.1125	4.9096	0.2354	2.1708
C	-1.9618	-6.7006	-3.0807	0.0584	0.4940	O	-5.9197	-8.2716	4.7465	0.0481	0.0276
C	-3.5291	-8.3046	-2.1808	-0.0482	-0.0014	O	-6.2655	-6.3345	3.5847	-0.1661	-1.3190
C	-1.515	-7.5693	-4.0495	-0.0494	-0.0023	C	-7.2673	-8.0587	4.6192	-0.1637	-1.3176
C	-3.0881	-9.1923	-3.1611	-0.0497	-0.0122	C	-7.477	-6.8825	3.9137	0.0595	0.4864
H	-4.2928	-8.5749	-1.4639	0.0530	0.0437	C	-8.3096	-8.8291	5.0806	0.0605	0.4878
C	-2.1027	-8.8334	-4.0748	-0.0502	-0.0122	C	-8.74	-6.4151	3.6321	-0.0471	0.0005
H	-0.7465	-7.2795	-4.7536	0.0527	0.0434	C	-9.5944	-8.3659	4.8023	-0.0449	0.0020
H	-3.5221	-10.1827	-3.2081	0.0418	0.0209	H	-8.1361	-9.745	5.6289	-0.0465	-0.0099
H	-1.7826	-9.5484	-4.8216	0.0418	0.0211	C	-9.8046	-7.1868	4.0945	0.0542	0.0462
O	-1.5692	-5.4156	-2.8336	-0.1711	-1.3243	H	-8.8928	-5.4979	3.08	-0.0457	-0.0092
O	-3.1867	-6.0072	-1.3332	-0.1658	-1.3192	H	-10.4467	-8.9384	5.1444	0.0564	0.0504
B	-2.2898	-5.0223	-1.7163	0.2204	2.1851	H	-10.8172	-6.8599	3.8968	0.0442	0.0253
H	0.4424	0.4571	5.3772	0.0430	0.0205	O	1.0592	-5.9405	2.136	0.0448	0.0264
H	-2.2816	3.7531	-3.281	0.0470	0.0265	O	-0.5201	-5.7458	-0.0649	-0.1583	-1.1497
O	-3.8579	1.1731	0.776	0.3794	2.9271	C	0.6162	-5.6855	-0.2468	0.3324	2.2598
P	0.7361	-6.3427	3.5369	0.3703	2.9245	O	1.7261	-5.7785	-0.5053	-0.1750	-1.1293

Table S8. Atomic coordinates, Hirshfeld (Q^H) and AIM (Q^B) atomic charges for **BP1'**-CH_4 adduct.

Atom	*x*	*y*	*z*	Q^H / e	Q^B / e	Atom	*x*	*y*	*z*	Q^H / e	Q^B / e
P	-3.2363	0.9936	-1.0622	0.1045	1.4244	C	2.439	-2.1217	4.7275	-0.0483	-0.5127
C	-1.9959	1.7006	0.1014	-0.0479	-0.5171	C	4.0856	-3.8532	3.1697	-0.0390	-0.5128
C	-0.9846	0.9566	0.7081	-0.0392	-0.0160	C	3.4704	-1.223	2.2169	-0.0481	-0.5159
C	-2.1154	3.053	0.4301	-0.0379	-0.0159	C	3.3577	-2.3125	5.7563	-0.0372	-0.0170
C	-0.1108	1.5521	1.6095	-0.0387	-0.0172	C	1.2363	-1.4732	5.0135	-0.0371	-0.0151
H	-0.8672	-0.0915	0.4725	0.0341	0.0368	C	3.7982	-5.1202	3.6874	-0.0368	-0.0156
C	-1.2385	3.6498	1.3206	-0.0407	-0.0176	C	5.3891	-3.5762	2.7659	-0.0403	-0.0168
H	-2.9082	3.6432	-0.0166	0.0409	0.0238	C	4.0929	-0.1967	2.925	-0.0365	-0.0172
C	-0.2326	2.8986	1.9155	-0.0388	-0.0187	C	3.4305	-1.1475	0.8241	-0.0352	-0.0171
H	0.6738	0.9604	2.0658	0.0404	0.0301	C	3.085	-1.852	7.0381	-0.0393	-0.0176
H	-1.3434	4.7013	1.5582	0.0436	0.0206	H	4.2919	-2.8242	5.5603	0.0367	0.0368
C	-2.8512	-0.8095	-1.0173	-0.0395	-0.5075	C	0.9678	-1.0042	6.29	-0.0387	-0.0165
C	-3.5913	-1.6125	-0.1522	-0.0365	-0.0136	H	0.5009	-1.3405	4.2273	0.0410	0.0269
C	-1.8563	-1.4033	-1.7979	-0.0355	-0.0155	C	4.793	-6.0707	3.8234	-0.0256	-0.0131
C	-3.3444	-2.9727	-0.0608	-0.0256	-0.0131	H	2.7837	-5.3613	3.9848	0.0432	0.0292
H	-4.372	-1.1644	0.4523	0.0415	0.0281	C	6.3824	-4.5363	2.8915	-0.0255	-0.0142
C	-1.6045	-2.7606	-1.7005	-0.0258	-0.0153	H	5.6338	-2.6056	2.3537	0.0354	0.0386
H	-1.2689	-0.795	-2.4726	0.0375	0.0375	C	4.6718	0.872	2.2538	-0.0389	-0.0185
C	-2.3449	-3.5694	-0.8308	-0.0743	-0.6823	H	4.1228	-0.2283	4.0069	0.0363	0.0380
H	-3.9303	-3.5815	0.6176	0.0456	0.0364	C	4.0182	-0.0857	0.1518	-0.0378	-0.0163
H	-0.8174	-3.206	-2.2974	0.0447	0.0352	H	2.932	-1.9301	0.2628	0.0419	0.0282
C	-2.5796	1.4732	-2.7106	-0.0485	-0.5199	C	1.8933	-1.1949	7.308	-0.0377	-0.0175
C	-1.3621	2.1141	-2.9193	-0.0394	-0.0182	H	3.8093	-2.0096	7.8282	0.0443	0.0224
C	-3.3954	1.2029	-3.8109	-0.0373	-0.0142	H	0.0292	-0.5032	6.4935	0.0455	0.0246
C	-0.9627	2.4659	-4.2029	-0.0404	-0.0180	C	6.1068	-5.7969	3.4254	-0.0779	-0.6879
H	-0.7187	2.3392	-2.0776	0.0358	0.0372	H	4.5509	-7.0439	4.234	0.0455	0.0365
C	-2.9907	1.5405	-5.0922	-0.0393	-0.0161	H	7.3914	-4.3016	2.5727	0.0448	0.0352
H	-4.3558	0.7217	-3.6606	0.0414	0.0254	C	4.6399	0.9284	0.8672	-0.0363	-0.0173
C	-1.7718	2.1772	-5.2914	-0.0397	-0.0186	H	5.1498	1.6639	2.818	0.0443	0.0222
H	-0.012	2.9641	-4.3496	0.0432	0.0199	H	3.9813	-0.0451	-0.93	0.0455	0.0243
H	-1.4584	2.4521	-6.2909	0.0436	0.0206	H	1.6823	-0.8401	8.3092	0.0445	0.0225
C	-2.2129	-7.1922	-0.1909	0.0600	0.4918	B	7.2127	-6.8558	3.5647	0.2329	2.1690
C	-1.0802	-7.0211	-0.9728	0.0603	0.4913	H	5.091	1.7641	0.3464	0.0450	0.0231
C	-2.5724	-8.4195	0.3158	-0.0473	0.0003	O	8.536	-6.6662	3.1905	-0.1664	-1.3190
C	-0.2447	-8.0678	-1.2871	-0.0466	0.0006	O	7.0215	-8.1291	4.0828	-0.1663	-1.3189
C	-1.7326	-9.4882	0.0072	-0.0477	-0.0113	C	9.1674	-7.8438	3.4836	0.0594	0.4915
H	-3.4581	-8.5422	0.9242	0.0541	0.0458	C	8.2482	-8.7317	4.0252	0.0594	0.4915
C	-0.5948	-9.3162	-0.7756	-0.0474	-0.0112	C	10.4893	-8.1811	3.3055	-0.0477	-0.0005
H	0.6387	-7.9231	-1.8939	0.0545	0.0463	C	8.6041	-10.0019	4.416	-0.0476	-0.0006
H	-1.9722	-10.4738	0.3846	0.0433	0.0233	C	10.8625	-9.466	3.698	-0.0479	-0.0108
H	0.0329	-10.1704	-0.9934	0.0435	0.0236	H	11.1981	-7.4821	2.8826	0.0538	0.0455
O	-0.9785	-5.7033	-1.3307	-0.1655	-1.3202	C	9.9413	-10.3557	4.2406	-0.0479	-0.0108
O	-2.8424	-5.9848	-0.045	-0.1661	-1.3205	H	7.8802	-10.6864	4.8371	0.0539	0.0457
B	-2.0638	-5.0788	-0.743	0.2329	2.1707	H	11.8923	-9.7761	3.5769	0.0434	0.0238
H	0.4509	3.3629	2.6158	0.0439	0.0214	H	10.2678	-11.345	4.5339	0.0434	0.0238
H	-3.6324	1.3173	-5.9357	0.0443	0.0219	H	-0.7794	-4.8179	1.2987	-0.0483	-0.0069
P	2.6678	-2.6909	2.9906	0.1117	1.4363						

Table S9. Atomic coordinates, Hirshfeld (Q^H) and AIM (Q^B) atomic charges for **BPO1'**-CH_4 adduct.

Atom	*x*	*y*	*z*	Q^H / e	Q^B / e	Atom	*x*	*y*	*z*	Q^H / e	Q^B / e
P	-2.664	0.2184	-0.4898	0.3730	+2.8988	C	4.3477	-1.8374	2.0789	-0.0497	-0.5831
C	-1.0181	0.6144	0.164	-0.0534	-0.5878	C	2.931	-2.6004	5.3791	-0.0348	-0.0205
C	0.133	-0.1009	-0.1553	-0.0309	-0.0199	C	1.088	-2.2095	3.8763	-0.0201	-0.0032
C	-0.9367	1.6966	1.0396	-0.0249	-0.0082	C	3.9994	-5.8014	3.2792	-0.0216	-0.0043
C	1.3573	0.2719	0.3831	-0.0351	-0.0144	C	5.7499	-4.2057	3.7179	-0.0346	-0.0194
H	0.077	-0.9667	-0.8042	0.0397	+0.0378	C	4.7909	-0.8105	2.9083	-0.0312	-0.0189
C	0.2869	2.0671	1.5746	-0.0366	-0.0148	C	4.6858	-1.825	0.7263	-0.0206	-0.0108
H	-1.8422	2.2275	1.308	0.0496	+0.0577	C	2.1666	-2.1301	6.4368	-0.0389	-0.0147
C	1.4346	1.3574	1.2439	-0.0316	-0.0148	H	3.9362	-2.9609	5.5633	0.0371	+0.0279
H	2.2467	-0.2967	0.1425	0.0387	+0.0376	C	0.3261	-1.7415	4.9366	-0.0312	-0.0091
H	0.3447	2.9062	2.2572	0.0453	+0.0231	H	0.6734	-2.2634	2.8772	0.0497	+0.0707
C	-2.5993	-1.5333	-0.9672	-0.0435	-0.5787	C	4.8106	-6.8173	3.7543	-0.0206	-0.0089
C	-3.0766	-2.4556	-0.0374	-0.0223	-0.0054	H	3.009	-6.0185	2.8981	0.0528	+0.0637
C	-2.1021	-1.9806	-2.1884	-0.0330	-0.0183	C	6.5574	-5.227	4.1936	-0.0250	-0.0120
C	-3.0346	-3.8103	-0.3204	-0.0194	-0.0068	H	6.1305	-3.192	3.6837	0.0375	+0.0319
H	-3.4896	-2.0949	0.8968	0.0505	+0.0630	C	5.5777	0.2107	2.3933	-0.0366	-0.0169
C	-2.0596	-3.3374	-2.4649	-0.0234	-0.0102	H	4.5082	-0.7949	3.9541	0.0388	+0.0348
H	-1.758	-1.2704	-2.9305	0.0378	+0.0307	C	5.4708	-0.8028	0.2144	-0.0329	-0.0144
C	-2.5202	-4.2733	-1.535	-0.0678	-0.6736	H	4.312	-2.613	0.0837	0.0517	+0.0642
H	-3.4028	-4.5224	0.4086	0.0481	+0.0405	C	0.8657	-1.698	6.2149	-0.0300	-0.0129
H	-1.6667	-3.6787	-3.4151	0.0461	+0.0376	H	2.583	-2.1105	7.4363	0.0453	+0.0242
C	-2.8342	1.1584	-2.0326	-0.0523	-0.5839	H	-0.6915	-1.4146	4.7638	0.0504	+0.0346
C	-1.7554	1.527	-2.8322	-0.0333	-0.0190	C	6.1003	-6.5469	4.2232	-0.0695	-0.6752
C	-4.1262	1.5209	-2.4076	-0.0223	-0.0056	H	4.4445	-7.8371	3.7585	0.0484	+0.0421
C	-1.9692	2.2343	-4.0069	-0.0387	-0.0148	H	7.5589	-5.0011	4.5403	0.0462	+0.0381
H	-0.7446	1.2797	-2.5303	0.0382	+0.0318	C	5.9198	0.2135	1.0479	-0.0300	-0.0147
C	-4.3365	2.2297	-3.5807	-0.0348	-0.0122	H	5.9172	1.009	3.0417	0.0464	+0.0259
H	-4.9545	1.2553	-1.762	0.0508	+0.0643	H	5.7289	-0.7964	-0.8374	0.0481	+0.0288
C	-3.2593	2.5826	-4.3828	-0.0320	-0.0139	H	0.2683	-1.3359	7.0426	0.0472	+0.0265
H	-1.127	2.522	-4.624	0.0450	+0.0232	B	7.0019	-7.6801	4.7513	0.2351	+2.1704
H	-3.4245	3.1379	-5.298	0.0460	+0.0239	H	6.5307	1.0129	0.6469	0.0476	+0.0274
C	-2.6836	-7.9508	-1.6744	0.0606	+0.4939	O	8.2894	-7.4979	5.2308	-0.1660	-1.3192
C	-2.0719	-7.6699	-2.8882	0.0603	+0.4909	O	6.6316	-9.0137	4.8101	-0.1647	-1.3183
C	-2.9566	-9.2393	-1.2764	-0.0468	+0.0012	C	8.7203	-8.7461	5.5919	0.0596	+0.4888
C	-1.7028	-8.6627	-3.7663	-0.0472	+0.0003	C	7.7133	-9.6664	5.3366	0.0601	+0.4900
C	-2.5872	-10.2545	-2.1577	-0.0476	-0.0102	C	9.9318	-9.1196	6.1264	-0.0470	+0.0002
H	-3.4319	-9.4478	-0.3276	0.0543	+0.0462	C	7.8661	-11.0077	5.6025	-0.0463	+0.0008
C	-1.9745	-9.9728	-3.3744	-0.0477	-0.0106	C	10.0989	-10.4762	6.4005	-0.0467	-0.0098
H	-1.2252	-8.4332	-4.7092	0.0539	+0.0452	H	10.7106	-8.3949	6.3216	0.0543	+0.0463
H	-2.7818	-11.2843	-1.8872	0.0433	+0.0233	C	9.0895	-11.3988	6.1445	-0.0465	-0.0097
H	-1.7015	-10.7876	-4.0325	0.0432	+0.0231	H	7.0753	-11.7171	5.3996	0.0550	+0.047
O	-1.9268	-6.315	-3.0146	-0.1652	-1.3197	H	11.0355	-10.8177	6.8217	0.0441	+0.0252
O	-2.9344	-6.7786	-1.0159	-0.1642	-1.3197	H	9.256	-12.444	6.3704	0.0443	+0.0255
B	-2.4602	-5.7803	-1.8519	0.2374	+2.1706	O	2.4249	-3.7339	1.5704	-0.4162	-0.0098
H	2.3898	1.6414	1.6685	0.0416	+0.0261	H	0.0103	-6.3491	-0.3772	-0.4124	-0.0200
H	-5.3421	2.5122	-3.8667	0.0468	+0.0261	C	0.6652	-6.7007	0.4186	-0.0497	+0.0479
O	-3.7577	0.5083	0.4758	0.3740	+2.8996	H	1.2894	-5.8727	0.7522	-0.0348	-0.0082
P	3.3169	-3.2206	2.6449	-0.0537	-0.5912	H	0.0587	-7.0625	1.2477	-0.0201	-0.0086
C	2.3974	-2.6334	4.0924	0.3730	-0.5777	H	1.2869	-7.5118	0.0423	-0.0216	-1.4624

11.References for Supporting Information

[1] Y. Chung, B. F. Duerr, T. A. McKelvey, P. Nanjappan, A. W. Czarnik, *J. Org. Chem.* **1989**, *54*, 1018–1032.

[2] Y. V. Shklyaev, M. A. Yeltsov, Y. S. Rozhkova, A. G. Tolstikov, V. M. Dembitsky, *Heteroatom Chemistry* **2004**, *15*, 486–493.

[3] T. S. Balaban, A. Eichhöfer, M. J. Krische, J.-M. Lehn, *Helvetica Chimica Acta* **2006**, *89*, 333–351.

[4] J. N. Smith, J. M. Hook, N. T. Lucas, *J. Am. Chem. Soc.* **2018**, *140*, 1131–1141.

[5] C. Liu, Y. Li, Y. Li, C. Yang, H. Wu, J. Qin, Y. Cao, *Chem. Mater.* **2013**, *25*, 3320–3327.

[6] G. Ashiotis, A. Deschildre, Z. Nawaz, J. P. Wright, D. Karkoulis, F. E. Picca, J. Kieffer, *J Appl Cryst* **2015**, *48*, 510–519.

[7] *CrysAlis Pro Software*, Oxford Diffraction Ltd., Oxford, U.K., **2010**.

[8] G. M. Sheldrick, *Acta Cryst A* **2008**, *64*, 112–122.

[9] G. M. Sheldrick, *Acta Cryst C* **2015**, *71*, 3–8.

[10] C. Andersson, R. Larsson, *Journal of Catalysis* **1983**, *81*, 194–203.

[11] H. M. El-Kaderi, J. R. Hunt, J. L. Mendoza-Cortés, A. P. Côté, R. E. Taylor, M. O'Keeffe, O. M. Yaghi, *Science* **2007**, *316*, 268–272.

[12] J. Stewart, *Journal of molecular modeling* **2008**, *13*, 1173–213.

[13] J. J. P. Stewart, *MOPAC2016*, Stewart Computational Chemistry, Colorado Springs, CO, USA, HTTP://OpenMOPAC.Net, **n.d.**

[14] S. Grimme, *J Comput Chem* **2004**, *25*, 1463–1473.

[15] S. Grimme, *J Comput Chem* **2006**, *27*, 1787–1799.

[16] R. Dovesi, R. Orlando, B. Civalleri, C. Roetti, V. R. Saunders, C. M. Zicovich-Wilson, *Zeitschrift für Kristallographie - Crystalline Materials* **2009**, *220*, 571–573.

[17] R. Dovesi, R. Orlando, B. Civalleri, C. Roetti, V. R. Saunders, and C. M. Zicovich-Wilson,, Z. Kristallogr., **2005**, *220*, 571.

[18] Y. Zhao, D. G. Truhlar, *Theor Chem Account* **2008**, *120*, 215–241.

[19] T. H. Dunning, *J. Chem. Phys.* **1989**, *90*, 1007–1023.

[20] M. J. Frisch, G. W. Trucks, H. B. Schlegel, G. E. Scuseria, M. A. Robb, J. R. Cheeseman, G. Scalmani, V. Barone, G. A. Petersson, H. Nakatsuji, X. Li, M. Caricato, A. V. Marenich, J. Bloino, B. G. Janesko, R. Gomperts, B. Mennucci, H. P. Hratchian, J. V. Ortiz, A. F. Izmaylov, J. L. Sonnenberg, D. Williams-Young, F. Ding, F. Lipparini, F. Egidi, J. Goings, B. Peng, A. Petrone, T. Henderson, D. Ranasinghe, V. G. Zakrzewski, J. Gao, N. Rega, G. Zheng, W. Liang, M. Hada, M. Ehara, K. Toyota, R. Fukuda, J. Hasegawa, M. Ishida, T. Nakajima, Y. Honda, O. Kitao, H. Nakai, T. Vreven, K. Throssell, J. A. Montgomery, Jr., J. E. Peralta, F. Ogliaro, M. J. Bearpark, J. J. Heyd, E. N. Brothers, K. N. Kudin, V. N. Staroverov, T. A. Keith, R. Kobayashi, J. Normand, K. Raghavachari, A. P. Rendell, J. C. Burant, S. S. Iyengar, J. Tomasi, M. Cossi, J. M. Millam, M. Klene, C. Adamo, R. Cammi, J. W. Ochterski, R. L. Martin, K. Morokuma, O. Farkas, J. B. Foresman, and D. J. Fox, *Gaussian 16, Revision C.01*, Gaussian, Inc., Wallingford CT, **2016**.

[21] Todd A. Keith, *AIMAll (Version 19.10.12)*, TK Gristmill Software, Overland Park KS, USA, **2019**.

[22] R. F. W. Bader, *Atoms in Molecules: A Quantum Theory*, Oxford University Press, Oxford, New York, **1994**.
